# The propagation of perturbations in rewired bacterial gene networks

**DOI:** 10.1038/ncomms10105

**Published:** 2015-12-16

**Authors:** Rebecca Baumstark, Sonja Hänzelmann, Saburo Tsuru, Yolanda Schaerli, Mirko Francesconi, Francesco M. Mancuso, Robert Castelo, Mark Isalan

**Affiliations:** 1 EMBL/CRG Systems Biology Research Unit, Centre for Genomic Regulation (CRG), Dr Aiguader 88, 08003 Barcelona, Spain; 2 Universitat Pompeu Fabra (UPF), Dr Aiguader 88, 08003 Barcelona, Spain; 3 Research Program on Biomedical Informatics (GRIB), Hospital del Mar Medical Research Institute (IMIM), Dr Aiguader 88, 08003 Barcelona, Spain; 4 Department of Experimental and Health Sciences, Universitat Pompeu Fabra, Dr Aiguader 88, 08003 Barcelona, Spain; 5 Department of Bioinformatic Engineering, Graduate School of Information Science and Technology, Osaka University, 1-5 Yamadaoka, Suita, Osaka 565-0871, Japan; 6 Genomics Cancer Group, Vall d 'Hebron Institute of Oncology (VHIO), Carrer Natzaret 15-17, 08035 Barcelona, Spain; 7 Department of Life Sciences, Imperial College London, London SW7 2AZ, UK

## Abstract

What happens to gene expression when you add new links to a gene regulatory network? To answer this question, we profile 85 network rewirings in *E. coli.* Here we report that concerted patterns of differential expression propagate from reconnected hub genes. The rewirings link promoter regions to different transcription factor and σ-factor genes, resulting in perturbations that span four orders of magnitude, changing up to ∼70% of the transcriptome. Importantly, factor connectivity and promoter activity both associate with perturbation size. Perturbations from related rewirings have more similar transcription profiles and a statistical analysis reveals ∼20 underlying states of the system, associating particular gene groups with rewiring constructs. We examine two large clusters (ribosomal and flagellar genes) in detail. These represent alternative global outcomes from different rewirings because of antagonism between these major cell states. This data set of systematically related perturbations enables reverse engineering and discovery of underlying network interactions.

## Abstract (web summary)

Expression of transcription factors to alter gene regulation can cause substantial changes to expression across a genome. Here the authors ‘rewire’ *E. coli* and analyse the global transcriptome alterations to identify novel network interactions.

## Graphical abstract

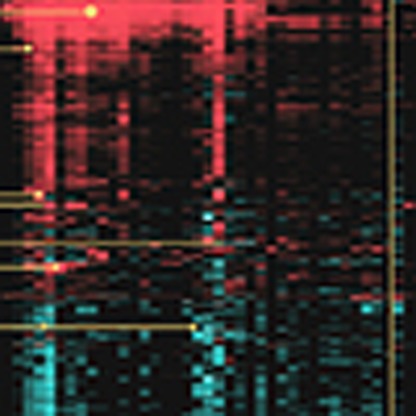

One aim of systems biology is to have a predictive understanding of biological systems, such that the effects of altering one or more components could be inferred a priori. *Escherichia coli* is one of the best studied organisms, and detailed regulatory network databases, such as RegulonDB^1^ and EcoCyc^2^, ultimately promise a complete description of all the connections between genes in the system^3^,^4^. Moreover, detailed metabolic network models have also been developed^5^,^6^, some incorporating transcriptional regulation^7^. Therefore it is reasonable to ask to what extent these network descriptions give us any predictive power over the effect of perturbations at one or more nodes of the network.

Although most large-scale perturbation studies have relied on environmental alterations, gene knockouts and—to a lesser extent—gene overexpression^4^,^8^, we previously described a complementary way of tinkering with *E. coli* transcription networks, namely by rewiring or adding new network connections^9^. By introducing fusions between promoter regulatory regions and open reading frames of transcription factors or σ-factors (TF ORFs), it is possible to connect all the inputs to a regulatory region to the downstream targets of the TF (Fig. 1). The result is that parallel network cascades can be linked with new cross-talks (Fig. 1b), and that new feedbacks can also be introduced (Fig. 1c). These new ‘links’ are added on top of the existing network and can result in large, complicated rewirings.

In our previous work^9^, we built ∼600 rewired networks and showed that the vast majority results in viable cells, even when reconnecting ‘hub genes’ that control hundreds or even thousands of other genes. This demonstrated that *E. coli* is highly tolerant of new regulatory connectivity that could result from evolutionary gene duplication, drift and deletion^10^,^11^. However, it was unclear how the resulting transcriptome perturbations spread across the whole network. From the only two examples assayed for differential expression using microarrays, rpoS–ompR had 10 out of ∼4,000 genes changed, whereas malT–fliA had 975 (using <5% false discovery rate^12^). There was evidence that FliA was upregulating flagellar machinery, as expected, but rpoS–ompR was difficult to explain in terms of annotated network interactions. Therefore, the two cases were quite different and it was unknown how the other rewired networks would behave.

In this study, we carry out gene expression profiling on 85 rewired networks, in biological triplicates, chosen to cover a range of related perturbations. Each promoter- and -ORF is sampled in the context of several different rewirings and is chosen to span a range of GFP expression values and growth phenotypes (see, for example, Fig. 2 of the original publication^9^; a GFP reporter is added as a second cistron to each promoter–ORF transcript). This enables us to report here the first comprehensive analysis of the propagation of changes across a rewired system, under defined conditions.

## Results

### Generating the rewired trancriptome networks

**Figure 2:**
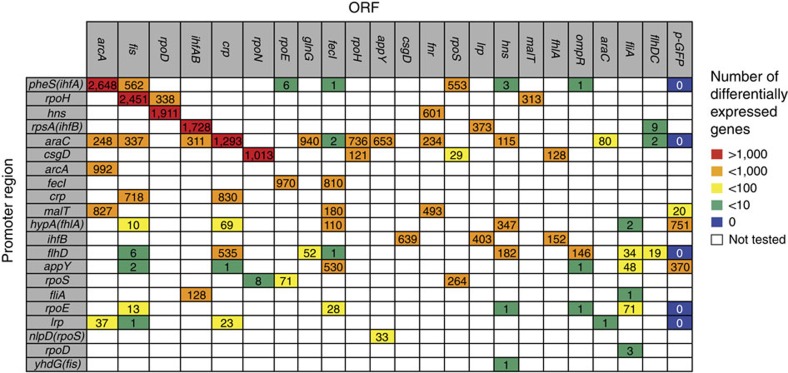
Global view of the number of differentially expressed genes (DEG) in each of the 85 rewired networks. Microarrays in triplicate repeats identified DEG (>1.2-fold change; 5% false discovery rate). Genes were limited to the 3,891 annotated MG1655 *E. coli* genes with unique Entrez Gene IDs, as identified by the R Bioconductor package ecoli2.db (ref. 54). The numbers of network perturbations are sorted top-bottom and left-right by the largest perturbations of individual promoters and ORFs. For example, pheS-arcA has 2,648 out of 3,891 genes perturbed (top left).

First, the 85 chosen gene network rewiring plasmids were transformed into *E. coli* and grown under standardized conditions (see Methods). Biological triplicates (separate colonies) of each population were subjected to RNA transcriptome analysis, using microarrays, and each promoter–ORF network was compared with a ‘wild-type’ reference standard (Co; Control). The differentially expressed genes were analysed to determine the scale and type of network perturbations. Taking a global view, the rewired networks produce perturbations spanning over four orders of magnitude, from 0 to thousands of differentially expressed genes (Fig. 2).

The log2-transformed fold gene expression data comprise a matrix of 85 rewired networks by 3,891 genes (MG1655 genes with Entrez IDs; Supplementary Data 1). This matrix provides a detailed view of rewiring perturbations, from which one can derive general patterns. As expected, the rewired ORF (-ORF) is the most differentially expressed gene in 72 out of 77 (93%) promoter–ORF combinations (the eight promoter-only control constructs are not included in this analysis because they do not express -ORFs). For two promoters, the -ORF is the second-most differentially expressed gene because the promoters contain embedded transcripts. Thus, *hycA* is most upregulated in hypA-crp and hypA-hns. Similarly, rpoS- promoters contain a highly expressed *nlpD* leader transcript. Overall, however, the greatest expression level changes originate from the -ORFs (2- to 600-fold; median: 13-fold; Supplementary Data 1).

### Transcriptome perturbations and promoter or ORF properties

Since we rewired genes with variable connectivity within the known regulatory hierarchy of *E. coli*, an open question was whether the more-connected genes would make bigger perturbations. We tested whether there was a correlation between the ORF mean transcriptome perturbations and -ORF out degree (direct connections from RegulonDB 7.2 (ref. 1). We found a significant correlation (*R*=0.61, *P*=0.002 (analysis of variance (ANOVA) F-test for linear regression; Supplementary Table 1). Overall, -ORF connectivity may explain ∼37% of the variance in mean transcription perturbation.

Promoters are also associated with rewiring perturbation sizes. There is a significant correlation between promoter activity (measured by quantitative real-time PCR (qRT–PCR) of the promoter-only constructs) and mean transcriptome perturbations per promoter (*R*=0.58, *P*=0.006 (ANOVA F-test); Supplementary Table 2). This is also true when correlating mean transcriptome perturbations to protein expression per promoter, using western blot data^9^ (*R*=0.55; *P*=0.009 (ANOVA F-test); Supplementary Table 2). Overall, promoter expression levels may explain ∼30–33% of the variance in mean transcription perturbation.

### Promoter effects without linked transcription factors

‘Promoter-only’ constructs contain GFP instead of a transcription factor -ORF and test for potential confounding effects such as high exogenous transcription, GFP-expression, or transcription factor titration by the regulatory region. Of the eight promoter-only constructs, five have zero perturbations, including some of the highest expression clones such as araC-0 (Supplementary Data 1). Thus, the majority of promoters do not alter the transcriptomes by themselves (Fig. 2; ‘p-GFP’ column). Furthermore, we see no evidence of transcription factors being systematically titrated to alter the transcriptome (using EcoCyc promoter-binding annotations).

**Figure 3:**
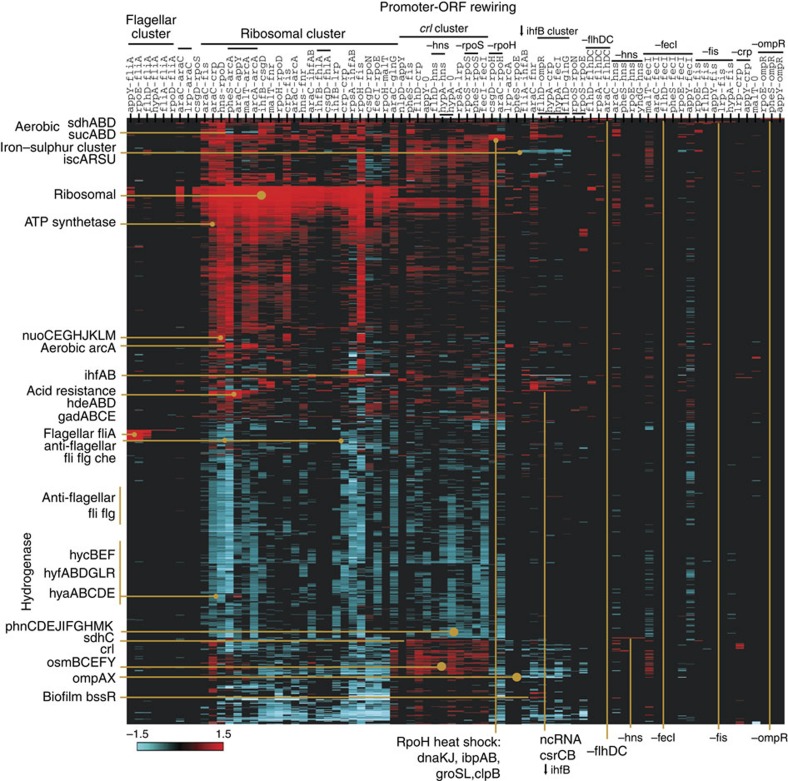
Hierarchical clustering of differential expression in rewired gene networks reveals clusters of concerted gene expression. Upregulated genes are in red; downregulated in blue. The 85 networks (columns) and 3,891 MG1655 *E. coli* genes (rows) were clustered by a correlation measure-based distance (uncentred, average distance UPMGA; EPCLUST^14^). The scale uses log2-transformed fold changes, relative to the control Co. Patterns or clusters of differential expression (for example, flagellar cluster and ribosomal cluster) are indicated, together with selected genes and operons. For display purposes, the five clones with zero changes are omitted (araC-0, flhD-0, pheS-0, rpoE-0, lrp-0); only 1,115 genes are displayed vertically, by filtering for genes which are differentially expressed in at least five networks and with an absolute sum differential expression >4. The full gene list and clustering are in Supplementary Data 1.

Two promoter-only clones make large perturbations: hypA-0 (751) and appY-0 (370) are quite similar, with 273 perturbations in common, including hydrogenase operon downregulations (*hyc, hyd, hyf* and *hya)* and osmotic signalling upregulations *(osmBCEFY)*. hypA- contains an embedded antiparallel *hycA* ORF in the promoter, which is expressed in hypA- constructs and may account for the states of the hydrogenase operons. The smaller perturbation in appY-0 is more difficult to explain, but shares the features of upregulated master regulators *rpoS, crp* and *crl.* Notably, *crl* stimulates RpoS activity during stationary phase^13^, which regulates *osm* genes. Several networks share this apparent gene expression state (Fig. 3; *crl* cluster). Because one aim in this study was to identify such common underlying states, and the key perturbations required to achieve them, we looked for such states by clustering the rewired constructs.

### Clustering is driven by promoter and ORF identity

We sorted the 85 networks by hierarchical clustering into groups with common patterns of transcriptome perturbation. Clustering allowed visualization of common reproducible states of the system: groups of whole operons and gene families which are up- or downregulated. We chose a correlation measure-based distance (Fig. 3, uncentred, average distance UPMGA; EPCLUST^14^; Supplementary Data 1). Importantly, clones were often grouped in columns according to promoter- and -ORF identity, and genes of similar pathways or function were grouped in rows. This shows that the introduced genetic perturbations (promoter–ORF fusions) drive the clustering.

Promoter- clusters are a relatively minor feature (for example, rpoS-, hypA-, araC-; Fig. 3). Some occur because of highly transcribed RNA sequences in the promoter regions, such as *nlpD* in rpoS- or *hycA* in hypA-. Alternatively, the araC- promoter is one of the strongest in our study^9^ and leads to larger perturbations (Fig. 2), which also cluster.

-ORF clusters are the most prominent feature of the correlation-based clustering (for example, -fliA, -flhDC, -fecI; Fig. 3). Nearly every -ORF has a characteristic RNA expression level (for example, -fecI is 13- to 15-fold upregulated; -arcA is 9- to 10-fold; Supplementary Data 1). As we observed previously^9^, ORFs appear to have a strong role in setting their own RNA and GFP protein expression levels, regardless of promoter identity. Therefore ORF expression control is not confined to the promoter regions.

Similar -ORF expression levels can lead to very different network perturbations. For example, pheS-fis (562 perturbations) and lrp-fis (1 perturbation), both have ∼17-fold *fis* RNA upregulation (Supplementary Data 1). Growth and expression (GFP-output) time courses reveal that these two networks have slightly different growth and protein expression dynamics (Supplementary Fig. 1), which results in very different transcriptomes. Differences can also be seen in pheS–arcA (2,649 perturbations) and arcA–arcA (992), which cannot be simply explained by a similar ∼10-fold upregulation of ArcA (Supplementary Fig. 2). Overall, it is imperative to consider each construct disrupting the network as a function of both promoter- and -ORF identity.

### Master regulators correlate with a ribosomal gene cluster

To see whether we could explain features of the transcriptome states for particular rewiring combinations, we next examined major clusters to look for common patterns of regulation.

The largest cluster in our study contains ∼125 upregulated ribosomal, ATP synthetase and tRNA genes and is associated with ∼25 rewiring constructs that make large perturbations (‘ribosomal cluster’; Fig. 3; Supplementary Data 1). These include 9 out of 13 araC- clones, four of five -arcA clones and both -rpoD clones, with perturbations ranging from 29–2,648 genes (median 646). Although the -ORFs involved are highly connected (for example, CRP, Fis, ArcA and IHFAB) and control hundreds of genes^1^, there was no immediate explanation as to why only certain rewired combinations have these effects. We, therefore, set out to find any common controlling factors between these apparently disparate rewired networks.

**Figure 4:**
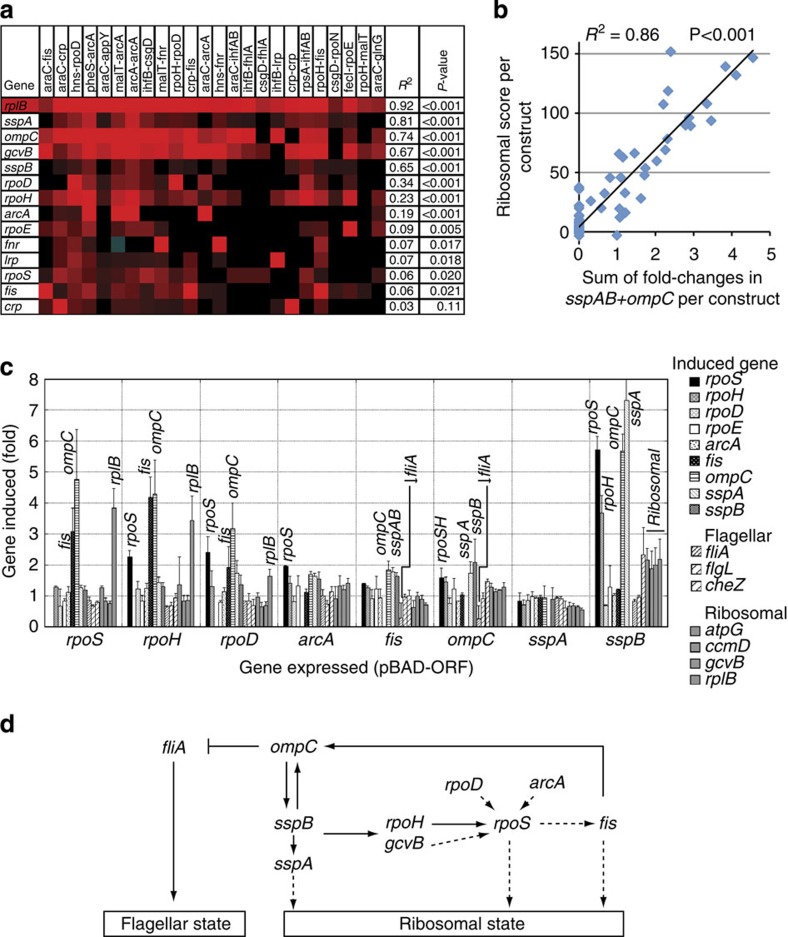
Expressing regulators that are correlated with the ribosome cluster is sufficient to induce ribosomal genes. (**a**) Expression heatmap of ribosomal cluster constructs versus the most-correlated sensor and regulator genes. The *rplB* gene is a representative ribosomal gene. Correlation coefficients (*R*^2^) are calculated by comparing the log2 fold-expression change of each gene, over each of the 85 constructs, versus the ‘ribosomal score’ of each construct (a sum of ∼125 ribosomal cluster genes, excluding regulators). (**b**) The regulators *sspAB* and *ompC* together could account for ∼86% of the variation seen in ribosome cluster genes. Other combinations of correlated factors do not associate as highly. (**c**) Eight cluster-associated regulator ORFs were expressed using the pBAD system (Invitrogen). qRT–PCR showed which of 16 genes were subsequently induced or repressed: σ-factors (*rpoSHDE*), master regulators (*arcA, fis*), periplasmic shock sensor (*ompC*), stringent starvation proteins (*sspAB*), flagellar genes (*fliA, flgL, cheZ*) and ribosomal cluster markers (*atpG, ccmD, gcvB, rplB*). (**d**) A minimum set of interactions (not necessarily direct) can be inferred from the qRT–PCR data, summarizing mutual upregulation of ribosomal state regulators. Dotted arrows indicate interactions reported in the literature.

When clustering the data (Supplementary Data 1), we found that several master regulators were consistently differentially expressed in the ribosome cluster. To assess levels of association between expression level changes of regulator genes and the ribosomal state, we used linear regression. We compared the correlation between the log2 fold-expression level of each of the 3,891 genes and the sum of log2 fold-expression of a set of ribosomal genes, across the rewired networks. To achieve this, we selected a representative set of ∼125 ribosomal cluster genes, excluding potential regulators in the set (ribosomal cluster; Supplementary Data 1). The correlation coefficients thus gave a ‘ribosomal score’ for the dose-dependent association of potential regulators with the ribosome cluster (Fig. 4a).

The four most-correlated potential regulators and sensors were the stringent starvation proteins *sspA* (*R*=0.90, *P*<0.001 (ANOVA F-test)) and *sspB* (*R*=0.80, *P*<0.001), *ompC* (porin, periplasmic sensor pathway protein^15^; *R*=0.86, *P*<0.001) and *gcvB* (regulatory RNA for acid resistance, amino acid transport and biosynthesis; *R*=0.82, *P*<0.001). Strikingly, *sspAB* and *ompC* levels could additively account for ∼86% of the variation in the ribosome cluster genes (Fig. 4b).

Other master regulators implicated in ribosomal biosynthesis also correlated with the ribosomal cluster in varying degrees (Fig. 4a). These included RpoH (σ32 for heat stress, drives *rrn* P1 ribosomal gene promoters^16^,^17^), RpoS (σ38 for stationary phase and acid resistance, implicated in ribosomal gene expression^18^,^19^), RpoD (σ70 for housekeeping and heat stress, drives many ribosomal promoters) and *fis* (Fis binding sites are found in *rrn* P1 ribosomal gene promoters^16^). To see which of these factors were necessary or sufficient to induce the ribosomal state in rewiring constructs, we induced their expression in *E. coli*.

### Expressing correlated master regulators upregulates ribosomes

Figure 4c shows that upregulation of key factors, using arabinose-inducible pBAD expression clones, could drive the cells towards a state with high ribosomal cluster gene expression. The gene that induced the strongest ribosomal response was *sspB*, which upregulated all the four ribosomal cluster genes assayed. *sspB* induced its partner *sspA*, but not vice versa. *sspA* and *sspB* are upregulated by carbon, amino acid and phosphate starvation and help ClpXP protease to degrade stalled protein synthesis^20^. *sspA* and *sspB* are sensor genes, thus linking potential metabolic perturbations to the ribosomal cluster response. *sspB* also upregulated *ompC* (and vice versa) demonstrating that the correlated genes in our regression analysis were indeed sufficient to elicit the ribosomal state.

We were unable to clone a pBAD-construct of *gcvB* (highly correlated with the ribosome cluster), which indicates that this noncoding RNA regulator may be toxic in this vector. Expression of *sspB* was, however, sufficient to upregulate *gcvB* and all the other ribosomal gene markers (Fig. 4c), indicating that *gcvB* is downstream of *sspB* in this regulatory network.

Expression of *arcA* directly upregulated ribosomal cluster master control genes (*ompC*, *sspAB*, *rpoHS*), as did expression of *rpoD* (*rpoS*, *fis*, *ompC*, *sspAB*). This is consistent with four -arcA clones and two -rpoD clones being in the ribosome cluster.

### Mutually upregulating factors induce the ribosome cluster

The results of pBAD-ORF expression are compatible with a circuit of mutual upregulation of a few master regulators that together achieve the ribosome state: *rpoH* induces *rpoS*, *fis* and *ompC; rpoS* induces *fis* and *ompC; fis* induces *ompC* and *sspAB*; *ompC* induces *sspAB* and *rpoHS*; *sspB* induces *rpoHS* and *ompC* (a minimal pruned set of inferred relationships is presented in Fig. 4d). Many of these interactions (for example, *rpoD*–*rpoS*, *arcA*–*rpoS* and *rpoS*–*fis*) are already well established^1^ and are indeed compatible with a recent effort to define a comprehensive sigma factor network in *E. coli*^21^.

As these factors are all interconnected (not necessarily directly), the ribosomal state would imply an ‘attractor basin’ within the transcriptional states caused by positive feedback between factors^22^. Thus, relatively small perturbations caused by expressing highly connected TFs (for example, CRP, Lrp, IHFAB) could begin to perturb key players in the system, such as *ompC*, *sspB*, *rpoDSH*, and start the cascade towards this upregulated state.

In certain cases, direct expression of the actual -ORF is the simplest explanation for the state seen: -arcA and -rpoD constructs upregulate the *ompC:sspAB* cascade, and also directly control ribosomal genes such as *rplB* (EcoCyc). However, in other cases, such as araC–crp or araC–ihfAB, the interaction is more indirect: factors that are uncorrelated to the ribosome cluster (for example, CRP, IHFAB), when expressed under a strong araC- promoter, are sufficient to perturb genes such as *rpoSH, ompC, sspB* and so on, to drive the response. Ultimately, the same set of identified regulatory genes is upregulated.

### Adding amino acids reproduces ribosomal state features

The ribosomal cluster response defines a large potential state of the system and so we investigated whether perturbations other than rewiring could induce it. Understanding the control of ribosomal biosynthesis is a major goal in itself (reviewed in ref. 17) and the ribosomal cluster is reminiscent of a classic ‘shift-up’ response, caused by adding amino acids after starvation. Starvation produces the stringent response metabolite ppGpp, which reduces RNA polymerase activity at P1 ribosomal promoters^23^,^24^,^25^; adding nutrients can reverse this.

**Figure 5:**
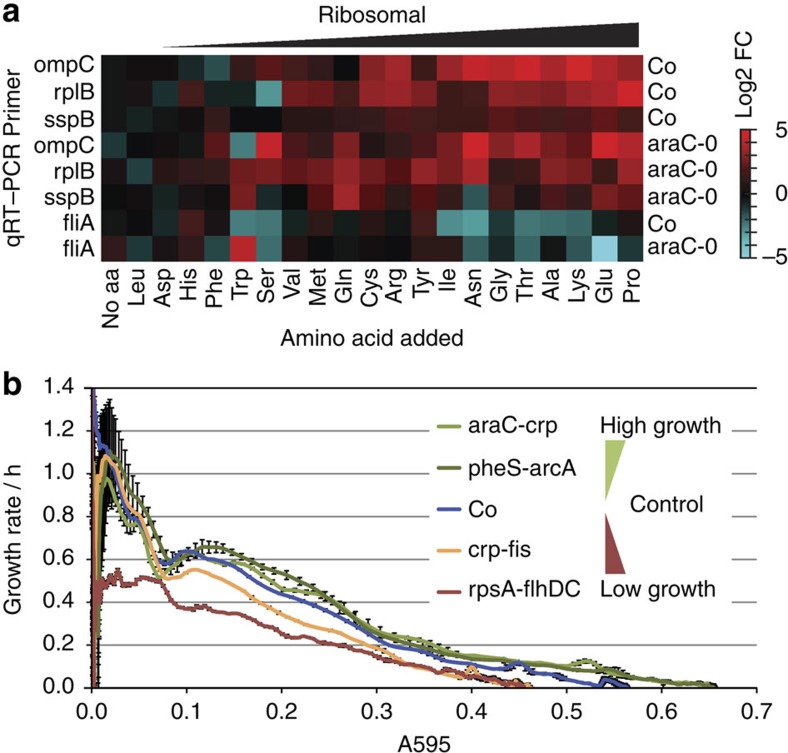
Amino acids affect ribosomal gene expression whereas growth rates do not correlate with the ribosomal state. (**a**) Adding 5 mM of individual amino acids generally increases ribosomal state marker genes and decreases *fliA*. qRT–PCR data are the means of three biological replicates (16 h growth) and are log2-transformed fold change, relative to levels in Co control in LB. (**b**) Growth rates at each *A*_595_ (absorbance at 595 nm) point in the growth curves of three ribosomal constructs and Co (Growth rate =d(ln *A*_595_)/dt; h^−1^). The ribosomal clone pheS–arcA (2,648 perturbations) actually grows faster and with a higher final optical density than Co. A slow-growing non-ribosomal rpsA–flhDC clone is included for comparison. Data are the means of three independent replicates with 1 s.d. error bars.

We tested whether simply adding additional amino acids to growth medium would induce key ribosomal factors for both control cells (Co), and for a construct with a strong promoter but low perturbation (araC-0; Fig. 5a). We found that certain amino acids (PEKATGNI) increase the ribosomal state genes in both genetic backgrounds. Adding other amino acids (FHDL) had little effect and was comparable to no addition. In conclusion, alterations in the availability of a subset of amino acids can reproduce the key features of the ribosomal state. Thus, altered amino acid availability, direct transcription perturbation via known regulators (for example, -arcA clones) and indirect perturbations (for example, araC–crp) are all ways of achieving the state.

### Higher growth rates in large-perturbation ribosomal clones

Recent work has shown that growth rates are intimately connected with gene expression changes, and faster growth is associated with greater ribosomal expression^26^,^27^. However, slow growing *E. coli* (for example, in glucose-limiting conditions) also upregulate ribosomes and break the general rule of ribosome biosynthesis being proportional to the square of growth rate^17^,^28^. Therefore, both slower and faster growth might also be expected to induce the ribosomal state, relative to the reference state (Co). In fact, we found that rewired constructs with high ribosomal gene expression do have altered growth (Fig. 5b and Supplementary Fig. 3).

Strikingly, two highly perturbed clones have higher growth rates than the control, and higher final optical densities: pheS–arcA (2,648 perturbations) and araC–crp (1,293). Notably, pheS–arcA is the most-altered clone in our study and yet—even with ∼70% of its transcriptome perturbed—the system appears to be able to reach a ‘super’ growth state, higher than wild type. These ribosomal clones may thus reflect a biological response to a more nutrient-rich state, in which the cells prepare more biosynthetic machinery and can grow faster.

Conversely, a key observation is that flagellar genes are downregulated in many ribosomal clones (Fig. 3; ‘anti-flagellar’ cluster). Expressing flagellar genes incurs a high metabolic cost and is required when the cells need motility to find new resources in nutrient-poor conditions^29^. Removing this cost may contribute to the higher overall growth of a subset of ribosomal clones. This potential link between nutrient-rich and nutrient-poor responses in ribosomal and flagellar constructs, respectively, suggested that rewirings are tapping into underlying biological responses. As the two major states in our study appeared to be linked, we set out to explore how the ribosomal state relates to the flagellar status.

### *fliA* upregulates flagellar genes whereas *ompC* represses them

The second largest cluster in our study consists of flagellar biosynthesis and chemotaxis genes (Fig. 3; ‘flagellar cluster’) and comprises -fliA clones. The flagellar sigma factor FliA is annotated as regulating many of these genes in RegulonDB^1^. By juxtaposing differential expressions from flagellar cluster clones onto the RegulonDB network, one can see that the direct partners of *fliA* are upregulated, as expected (Supplementary Fig. 4).

To obtain a ‘flagellar score’ for each rewired network, we calculated the sum of log2 fold-expression of ∼50 differentially expressed flagellar genes, excluding *fliA* (flagellar cluster; Supplementary Data 1). This score correlated better with *fliA* expression than with any other transcription regulator (*R*=0.61; *P*<0.0001 (ANOVA F-test)). Thus, the flagellar state agrees with current network annotations and a simple correlation analysis is sufficient to identify the cluster master regulator, FliA.

As mentioned above, the ribosomal state appears to be antagonistic to the flagellar state, because ribosomal cluster clones with higher ribosomal scores have increasingly ‘anti-flagellar’ states (Fig. 3). A clue to the basis of this antagonism came from our pBAD-ORF expression analysis, where expressing *ompC* (or upstream *fis*) is sufficient to repress *fliA* (Fig. 4c,d). This antagonism can also be inferred from the amino acid addition experiment (Fig. 5a), in which *fliA* is downregulated as *ompC* levels rise (linear regression of *fliA* versus *ompC* in araC-0 reveals a strong negative correlation: *R*=−0.79, *P*=0.00002 (ANOVA F-test). Thus, these two alternative states of the system are linked. This has a potential biological explanation as the flagellar machinery is required to move towards new nutrient sources in starvation, whereas the ribosomal state represents a high-nutrient metabolic state where synthesizing flagellar machinery is not required.

In summary, clustering and correlation approaches can identify major common states of the system in response to different rewiring perturbations. These states can then be dissected individually to identify key molecular players and control mechanisms. Extending this, we decided to define objectively all the statistically significant clusters (potential states) ultimately to discover new underlying connections in the system.

### Statistics reveal around 20 underlying network clusters

An open question was how many underlying states or clusters would be sufficient to describe the system. Several methods exist to estimate such system properties, including biclustering and principal component analysis (PCA).

Biclustering (see Methods) allows genes to appear in more than one cluster, such that smaller clusters are not overwhelmed by larger ones (for example, ribosomal). We obtained 23 statistically significant biclusters (Supplementary Data 2) and found groups corresponding to the known clusters (for example, flagellar, subclusters 7, 15; ribosomal, subcluster 20), as well as new groups (for example, subcluster 5, peptidoglycan metabolic process—cell wall synthesis).

We also carried out a PCA of the cluster matrix (see Methods). Twenty-one principal components explain 78% of the variance (Supplementary Fig. 5). The components contain groups of genes that are clustered in Fig. 3 and in the biclustering (for example, PC1: ribosomal -arcA, -rpoD, -fnr clones 17%; PC2: *crl* cluster as well as the neighbouring clusters with *osmBCEFY* and *ompAX* downregulation 7%; PC3: -fis 5%; PC4: -fecI 5%; PC5: -fliA 5%; and so on.).

The complementary ways of analysing the microarrays are consistent with the view that most of the rewiring perturbation data can be reduced to around 20 groups of underlying gene expression states, representing key biological features of the system.

### Reverse engineering the network with rewired perturbations

Rewiring the *E. coli* network can potentially reveal underlying regulatory interactions and we searched for these by reverse engineering a transcriptional network from the gene expression microarray data. For this purpose, we used a Gaussian graphical modelling technique implemented in the R/Bioconductor package qpgraph^30^. This method is based on higher-order conditioning to try to distinguish direct associations from indirect ones. The latter often occur owing to technical biases^31^ and strong genetic and molecular interactions that propagate through the network^32^.

**Figure 6:**
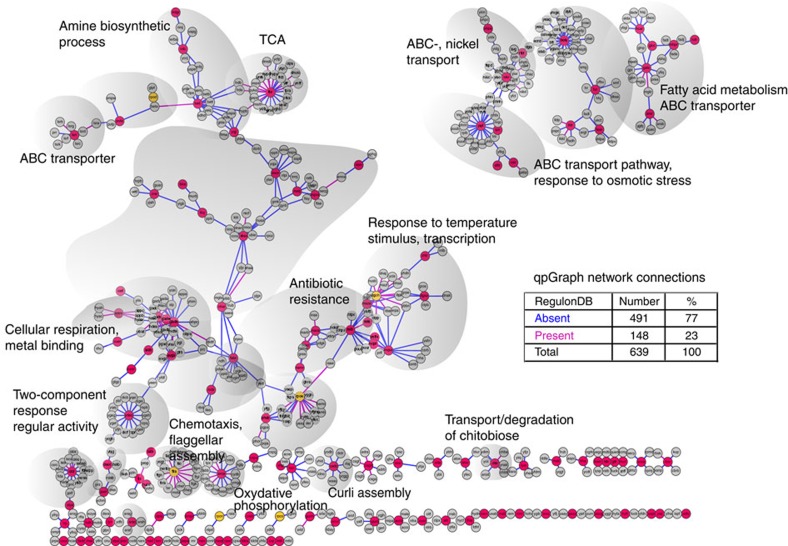
Reverse engineering network connections with qpgraph. Connected nodes are shown as circles (red indicates transcription factor (TF); yellow is σ-factor; grey indicates regulated gene). Edges (direct predicted connections) are shown by lines (blue indicates absent in RegulonDB^1^; magenta indicates present in RegulonDB). A precision of 30% was chosen to maximize the recall rate of RegulonDB interactions (Precision = Percentage number of true positives per number of predicted edges whose genes belong to at least one RegulonDB interaction). The inset table outlines the number of interactions recovered by qpgraph^30^, divided into interactions that are present or absent in RegulonDB. The network is annotated with Gene Ontology (GO) terms.

The large coverage of transcriptional regulatory interactions of *E. coli* in the literature is summarized in RegulonDB^1^. This gold standard network allows for reverse engineering estimates of that network at nominal values of precision or recall. Concretely, using qpgraph, we obtained a first estimate of the network at 30% precision (Precision = Percentage number of true positives per number of predicted edges whose genes belong to at least one RegulonDB interaction; Fig. 6). The network is formed by 643 nodes (genes) and 639 edges (interactions), organized into 64 connected components (modules). The network provided 491 new potential connections (Supplementary Data 4). These have 3% overlap with new (non-RegulonDB) interactions suggested by an independent DREAM project community prediction that uses multiple reverse engineering methods^33^ (Supplementary Fig. 6a). We also calculated a smaller, higher precision network (50% precision; Supplementary Fig. 7). The resulting network has 270 genes, 224 interactions and 56 modules; here there are 130 new potential interactions (Supplementary Data 5), which overlap 2% with new DREAM predictions (Supplementary Fig. 6b). The overlap with the DREAM study is relatively low, which shows that rewired networks are an alternative resource for discovering regulatory interactions by reverse engineering approaches.

We set out to validate some of the predicted interactions using publicly available data derived from binding assays, such as ChIP-chip and ChIP-seq, for nine transcription factors (GlnG^34^, Fnr^35^, ArgR, Lrp, TrpR^36^, Fur^37^, ArcA^38^, RpoD and RpoS^21^). We found that qpgraph gave target gene predictions, at 30% precision, for seven of them (Fnr, ArgR, Lrp, Fur, ArcA, RpoD, RpoS; Supplementary Data 3). Among these, we assessed the enrichment of targets reported in the binding assays among the targets predicted with qpgraph. In four of these seven cases, there were fewer than four predicted targets, and therefore, enrichment could not be reliably verified. However, the other three (Fnr, Fur and ArcA) had 29, 10 and 18 predicted target genes, respectively, and in the case of Fnr and ArcA, their predicted targets were significantly enriched by targets reported in the corresponding binding assays (one-tailed Fisher’s exact *P*<0.001; Supplementary Data 5). When restricting the analysis to predictions and targets outside RegulonDB (version 8), only a few predictions could be considered, which again were enriched in reported targets albeit only significantly in the case of Fnr (one-tailed Fisher’s exact *P*<0.001). Interestingly, no Fur-predicted target genes overlapped the targets reported in the binding assay nor targets reported in RegulonDB (version 8) (Supplementary Data 5). This further indicates that rewirings create complementary perturbations to environmental changes.

We next chose one qpgraph-predicted subnetwork to explore by DNA footprinting, potentially linking the transcription factor ExuR to DNA replication and the SOS response, via new connections to *gmk* and *mazEF* (Supplementary Fig. 8a). To guide our search, we used the motif-prediction tool MEME^39^ and identified a potential motif logo for the transcription factor in annotated promoter regions (Supplementary Fig. 8b). MEME found potential ExuR sites in several promoters, most of which are already annotated in regulonDB (Supplementary Fig. 8c). Interestingly, MEME revealed a novel site in *gmk*, which supported the qpgraph prediction, although no site was found in *mazEF*. This ambiguity was explored experimentally using DNA footprinting to search for potential binding sites in the promoters. To verify the method, we first confirmed the RegulonDB binding sites exuT1 and exuT2 (Supplementary Fig. 8d). We found potential, but weak, ExuR sites in *gmk*, partly supporting the new interaction suggested by our reverse engineering and MEME. No footprinting evidence was found for the ExuR binding directly to mazEF, in agreement with MEME.

Validating predicted interactions requires using multiple methods, and is currently laborious. Nonetheless, these pilot attempts, using ChIP-seq or ChIP-chip analysis and footprinting, suggest that rewired networks can indeed act as a resource for discovering new regulatory interactions. Future work should attempt to validate these predictions in a higher-throughput manner.

## Discussion

In this study, we explored for the first time how the perturbations from promoter-ORF rewirings spread across the *E. coli* transcriptome, using a resource of 255 microarrays that represent 85 rewired networks. We found a wide range of perturbation sizes with many common patterns of genes differentially expressed between different rewirings.

The main aim was to identify the major common states of the system and to account for how the rewirings relate to those states. Overall, there were around 20 reoccurring gene expression clusters that contained patterns of related operon expression.

We found that a few master sensors and regulators (*sspAB*, *fliA*) and the outer membrane porin (*ompC*) account for the two largest clusters (ribosomal and flagellar). Furthermore, these states are linked: *ompC* upregulation is associated with the ribosomal state and ultimately leads to repression of *fliA* and the flagellar state. Complex and variable relationships between ribosomal and flagellar gene expression have been observed before in several contexts^19^,^40^,^41^,^42^,^43^,^44^,^45^,^46^,^47^, but have not been studied with respect to promoter–ORF rewirings.

Our data set allows to make some general predictions, which may be useful to synthetic biologists expressing TFs in bacteria. First, more highly connected ORFs and stronger promoters make larger perturbations. For example, rewiring ArcA, RpoD, CRP, Fis, Lrp, IHFAB ORFs, especially with stronger promoters such as araC- or malT-, is likely to yield the largest perturbations. Second, these larger perturbations (especially via -ArcA and -RpoD rewirings) are more likely to reach a ribosomal state via upregulating sspB, while downregulating FliA and flagellar machinery.

Although making large perturbations may sometimes be desirable when engineering new properties into bacteria^48^, it is usually more common to desire the opposite (that is, to keep the metabolic load to the bacteria low^49^,^50^) and thus avoid unexpected cell behaviour. To minimize perturbations, our results indicate that weaker promoters and less connected ORFs should be utilized. Furthermore, our previous results^9^ indicate that integrating promoter–ORF constructs into the *E. coli* genome, as single copies, reduces their expression by ∼150-fold on average, while maintaining phenotypes^9^. It is thus likely that genomic integrations will reduce the magnitude of the promoter–ORF perturbations that we study here with plasmids. Nonetheless, the situation that our plasmid system models is a very common one in genetic engineering, where recombinant plasmids often use a bacterial promoter to drive an effector gene, and the whole expression system is added on top of the existing genetic background of the *E. coli* cell.

A key question is how do new rewirings, in combinations never seen or optimized by evolution, signal information across the network and change gene expression in a coordinated way. There is a growing body of evidence that bacterial cells may not always need evolved signalling machinery to achieve the appropriate gene expression state and can switch stochastically^51^, but consistently, if the change provides a fitness advantage. Faster growing bacteria express genes at a higher average level^24^, reinforcing the expression of genes required for survival under particular conditions. Over time, the cells in a population thus naturally select the appropriate ‘attractor state’ expression levels and growth rates^22^,^26^,^48^,^52^,^53^. The rationale is that bacteria encounter too many different combinations of environmental stimuli to evolve signalling pathways for them all and thus selection ‘according to need’ could compensate for new conditions. This idea is intriguing in the context of rewired gene networks, which provide perturbations that have not been encountered before and yet give consistent outputs. A few examples of our rewired clones have measurable growth advantages, concurrent with large transcriptome perturbations that provide a source of variation (for example, araC–crp and pheS–arcA ribosomal clones). It is therefore possible that a combination of rewiring perturbations and fitness pressures contributed to some of the gene expression patterns that we observed.

Despite it being important to consider fitness pressures in regulating gene expression, it is clear that much of *E. coli* gene expression is coordinated by signalling and direct hard-wired interactions between factors^54^. Here, our set of rewired networks provides a different and potentially rich resource for reverse engineering such interactions. The rewirings generate new perturbations that complement those observed by changing growth conditions, inducing knockouts or simple overexpression. Expression profiling of rewired networks and reverse engineering from such data can then help to infer novel regulatory interactions. Further in depth analysis of this resource is likely to confirm many new interactions as well as verifying known ones.

In this study, we have acquired a data set showing what happens when new promoter–TF combinations are added on top of an existing bacterial network, revealing that around 20 underlying potential states of the system (differential gene expression clusters) account for most of the observed changes. Our previous rewiring data sets have provided material for meta-analyses^55^,^56^ and we hope that these new data will provide the systems and synthetic biology communities with a useful resource for studying *E. coli* gene expression in response to rewiring.

## Methods

### Affymetrix *E. coli* Genome 2.0 microarrays

The 85 chosen gene network rewiring plasmids (from ref. 9) were transformed into *E. coli* TOP10 cells and grown under standardized conditions: bacteria were freshly plated onto LB Agar plates (supplemented with 100 μg ml^−1^ ampicillin and 50 μg ml^−1^ streptomycin) and incubated overnight at 37 °C to form colonies. For each biological replicate, single colonies (maximum 3 days old) were used to inoculate separate 2 ml overnight cultures in LB containing 100 μg ml^−1^ ampicillin and 50 μg ml^−1^ streptomycin, in 14ml culture tubes. Constructs were grown for 37 °C, at 220 r.p.m. in an orbital shaker. The pre-cultures were diluted to an D600 of 0.0015 (B1:800 dilution) in 2ml of the same medium, in 14ml culture tubes. Constructs were grown for 16h at 37C, at 220 r.p.m. in an orbital shaker. These conditions were chosen to match previous work^9^. RNA was extracted with RNeasy Protect Bacteria Mini Kit (Qiagen, Cat. 74106) and 10 μg of extracted total bacterial RNA (integrity number >7.0) was used with Affymetrix GeneChip *E. coli* Genome 2.0 Arrays. Additional details are provided in Supplementary Methods.

For differential expression analysis, we extracted microarray data for the relative RNA expression levels of 3,891 annotated *E. coli* genes with unique Entrez IDs^57^. Data analysis was performed using the R programming language and the Bioconductor software packages^58^. Each Affymetrix chip was background adjusted, normalized and log2 transformed using the Robust Multichip Averaging algorithm^59^. Differential expression analysis was performed by using the Bioconductor package limma^60^. All network constructs (biological triplicates from different colonies) were compared with five biological replicates of a ‘wild-type’ reference standard (*E. coli* transformed with an empty promoterless GFP plasmid; denoted ‘Co’^9^). The genes which were differentially expressed (<5% false discovery rate^12^, >1.2-fold-change) were extracted and used to analyse the scale of network perturbations. MIAME compliant microarray data files are available at EBI ArrayExpress (ID: E-MTAB-3233).

### Clustering

EPCLUST (http://www.bioinf.ebc.ee/EP/EP/EPCLUST/)^14^ was used for clustering the array data of fold changes in mRNA levels. Linear regression correlation analysis was carried out on microarray data with StatPlus:mac.LE.2009 ANOVA F-test to calculate *P* values.

### Induced expression and qRT–PCR

ORFS were obtained via PCR from *E. coli* TOP10 genomic DNA, and were cloned using the pBAD202 Directional TOPO Expression Kit (Invitrogen, Cat. K4202-01). We grew verified clones in LB medium, with 30 μg ml^−1^ kanamycin and 0.002% arabinose, to induce the pBAD promoter. RNA was extracted for qRT–PCR with an RNeasy Protect Bacteria Mini Kit (Qiagen, Cat. 74106).

Reverse transcription was carried out with SuperScript II Reverse Transcriptase (Invitrogen, Cat. 18064-014), or the QuantiTect Reverse Transcription Kit (QIAGEN, Cat. 205314), according to the manufacturer’s instructions. RNA transcripts were quantified in a 10 μl RT–qPCR reaction, using the LightCycler 480 system (Roche). Primer sets were tested by melting curve analysis with *E. coli* genomic DNA. All the samples were normalized against levels of *gnd* housekeeping gene and expression fold change was calculated relative to *gnd*-normalized Co control.

### Biclustering and Gene Ontology Enrichment Analysis

Biclustering was done with the Iterative Signature Algorithm^61^, in the R Bioconductor *eisa* package (v. 1.4.1; ref. 55). The gene (features) and sample thresholds were set to 2.1 and 1.5, respectively. Gene filter cutoffs: genes were used with log2 fold changes >0.25 or <−0.25 in at least three points.

Gene ontology analysis (Supplementary Data 2) was performed using the R Bioconductor package GOstats (v. 2.18; ref. 59), for biological process, molecular functions and cellular components. Results were filtered using an adjusted *P* value cutoff of 0.05 (ref. 12).

### Principal component analysis

PCA analysis that finds linear combinations of the genes expressed that are both uncorrelated and account for as much variance as possible^63^. We used the Kaiser rule (to retain components with eigen values >1, such that a factor extracts at least as much variance as the equivalent of one original variable^64^). Thus, 21 PCs are obtained.

### Reverse engineering of transcriptional regulatory networks

We reverse engineered transcriptional regulatory networks by using Gaussian graphical modelling techniques. Since, in the context of microarray data, the number of random variables (*p*) representing genes is much larger than the number of observations (*n*) representing samples, classical Gaussian graphical model theory cannot be directly applied. Therefore, we used the Bioconductor package qpgraph^30^, the which is tailor-made for data with *p* >> *n* dimensions. We estimated the structure of the network by calculating a quantity called the non-rejection rate (NRR), which is based on partial correlations of order *q* < (*n*−2) (ref. 65). The NRR gave an estimate of the strength of a direct interaction between two genes and can be understood as a linear measure of association over all marginal distributions of size *q* that is calculated for every gene pair. We calculated NRRs for every possible value of *q* and obtained an average NRR^30^. We used the average NRR to rank all regulator–target associations and estimate precision-recall curves based on RegulonDB 7.2 (ref. 1). Precision = Percentage number of true positives per number of predicted edges whose genes belong to at least one RegulonDB interaction. Guided by these curves and knowledge of the underlying system, we finally selected 30 and 50% precision networks.

## Additional information

**Accession codes:** All underlying microarray data are freely available at EBI ArrayExpress, ID: E-MTAB-3233 (www.ebi.ac.uk/arrayexpress).

**How to cite this article:** Baumstark, R. *et al*. The propagation of perturbations in rewired bacterial gene networks. *Nat. Commun.* 6:10105 doi: 10.1038/ncomms10105 (2015).

## Supplementary Material

Supplementary Information Supplementary Figures 1-8, Supplementary Tables 1-2, Supplementary Methods and Supplementary References

Supplementary Data 1 Clusters of processed gene expression microarray data for the 85 rewired gene networks. Data are fold-expression relative to control (Co) of normalised log2-transformed raw data. The data are organised into tabs. Tab 1 contains a key to annotations. Tab 2 contains the full annotated matrix of expression data for 3891 genes × 85 networks, clustered using EPCLUST, using a correlation based measure to cluster similar rows (genes) and columns (rewired networks) together. Tab 3 contains the underlying data for main Figure 3. Tab 4 contains expression data for 125 ribosomal, ATP synthetase and tRNA genes, associated with the ribosome cluster. Tab 4 contains expression data for 50 flagellar and chemotaxis genes.

Supplementary Data 2 Biclusters of gene expression for 85 rewired gene networks. The rewired constructs are listed along the bottom of each heatmap (individual bicluster) and the genes in the subcluster are listed to the right. A color key of the log2 fold change of each sample is provided for each heatmap. A Gene ontology Enrichment analysis of each bicluster is provided to the left of each heatmap (where statistically significant).

Supplementary Data 3 Edges forming a qp-graph 30% precision network. 639 interactions are analysed.

Supplementary Data 4 Edges forming a qp-graph 50% precision network. 224 interactions are analysed.

Supplementary Data 5 Analysis comparing qpgraph predictions with experimental binding data analysis. ChIP-seq or ChIP-chip data are analysed for GlnG, Fnr, ArgR, Lrp, TrpR, Fur, ArcA, RpoD and RpoS datasets.

## Figures and Tables

**Figure 1:**
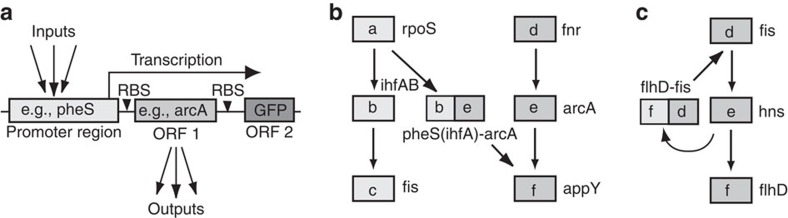
Rewiring the network by adding shuffled promoter–ORF combinations. (**a**) Plasmid constructs containing a promoter regulatory region, linked to a different ORF (transcription regulator or sigma factor), create new network links. A GFP ORF with an independent ribosome binding site (RBS) acts as a reporter. (**b**) Parallel pathways (‘a–b–c’ and ‘d–e–f’) can be linked together with a rewiring construct, b–e. The inputs to the promoter ‘b’ now output through the ORF ‘e’. Examples of actual gene regions used in this study are written next to the gene boxes. (**c**) Promoter regions downstream of their new partner ORF can create new feedback loops. The linear ‘d–e–f’ pathway is converted to a feedback loop, by adding the ‘f–d’ construct, such that the inputs into ‘f’ now output through ‘d’.
