# Supplementary material for: The propagation of perturbations in rewired bacterial gene networks
Source: Nat Commun. 2015 Dec 16;6:10105. doi: 10.1038/ncomms10105 (PMC4703840; doi:10.1038/ncomms10105)
Supplement: Supplementary Information — Supplementary Figures 1-8, Supplementary Tables 1-2, Supplementary Methods and Supplementary References [file ncomms10105-s1.pdf]

**Supplementary Figures**

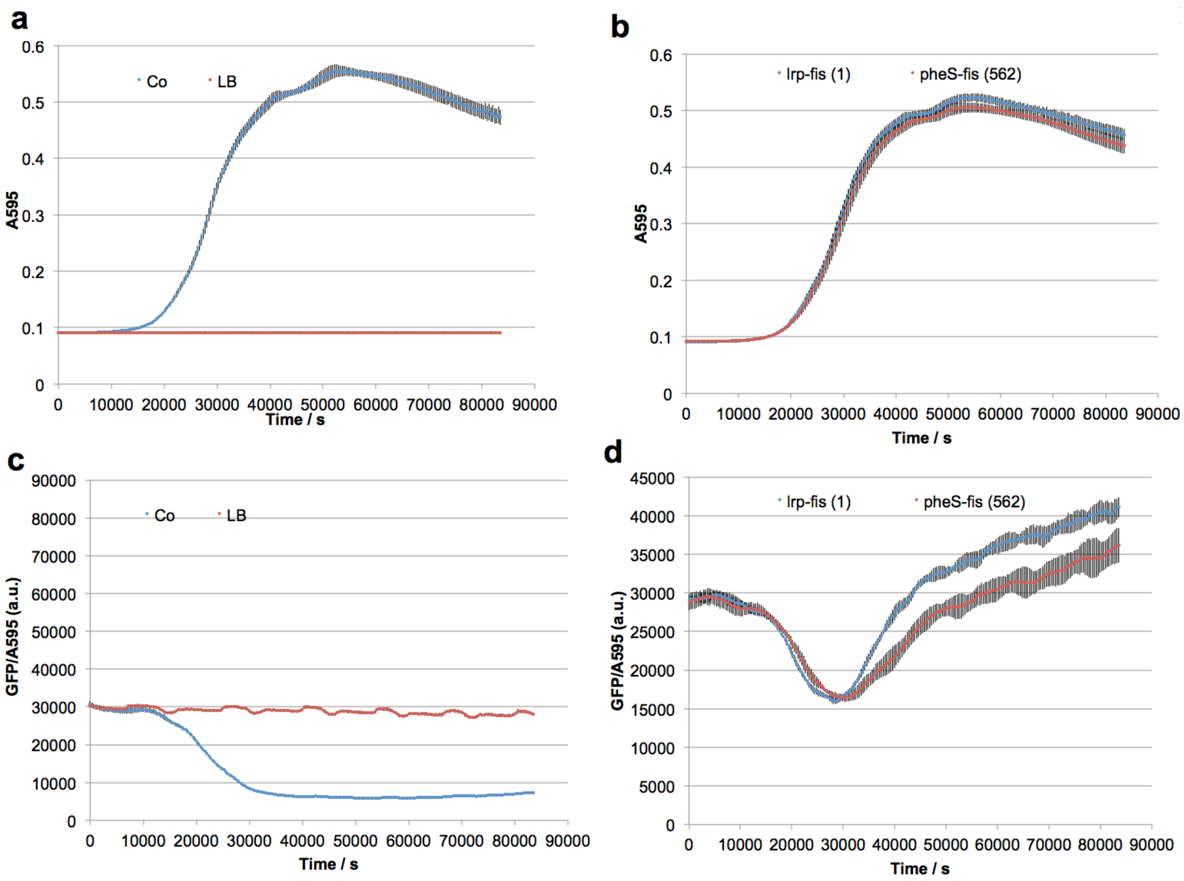


**Supplementary Figure 1. Growth and GFP-output timecourses for two -fis clones with very similar RNA levels in microarrays (~17-fold upregulation) but with very different transcriptome perturbations.** Bacterial colonies containing different plasmids were innoculated in 120 □l LB medium in 96-well plates (with 100 □g ml$^{-1}$ ampicillin and 50 □g ml$^{-1}$ streptomycin), and grown in a Tecan Infinite M200 platereader as described in the Supplementary Information of Isalan et al.[6], except that 5 ml overnight cultures were diluted to $OD_{600}$ of 0.0015 (around 1:800 dilution). Measurements of absorbance ($A_{595}$, measured at 595 nm) and GFP fluorescence were taken every 360 s. Blank LB medium was used as a control. The mean growth curve of 10 colonies was calculated after aligning all data on $A_{595}$=0.15. GFP fluorescence was normalised for $A_{595}$ to get a value of relative expression per unit number of cells. (**a**), Mean growth of control wells containing blank medium (LB; red) or Co promoterless GFP plasmid (Co; blue). (**b**), Mean growth of wells containing pheS-fis (562 transcriptome perturbations; red) or lrp-fis (1 perturbation; blue). (**c**), Mean GFP/$A_{595}$ of control wells containing blank medium (LB; red) or Co plasmid (Co; blue; no GFP). (**d**), Mean GFP/$A_{595}$ of wells containing pheS-fis (red) or lrp-fis (blue). Although the dynamics of growth and expression are only slightly different, pheS-fis grows slightly more slowly than lrp-fis, and has upregulation of stationary phase factors *rpoS* and *crl*, thus accounting for the larger perturbation (see main Figure 3). Notably, pheS-rpoS and several other rewirings also upregulate *rpoS* and *crl* and are in a similar transcriptome state. Error bars are 1 s.e.m. for 10 replicates (colonies) throughout.

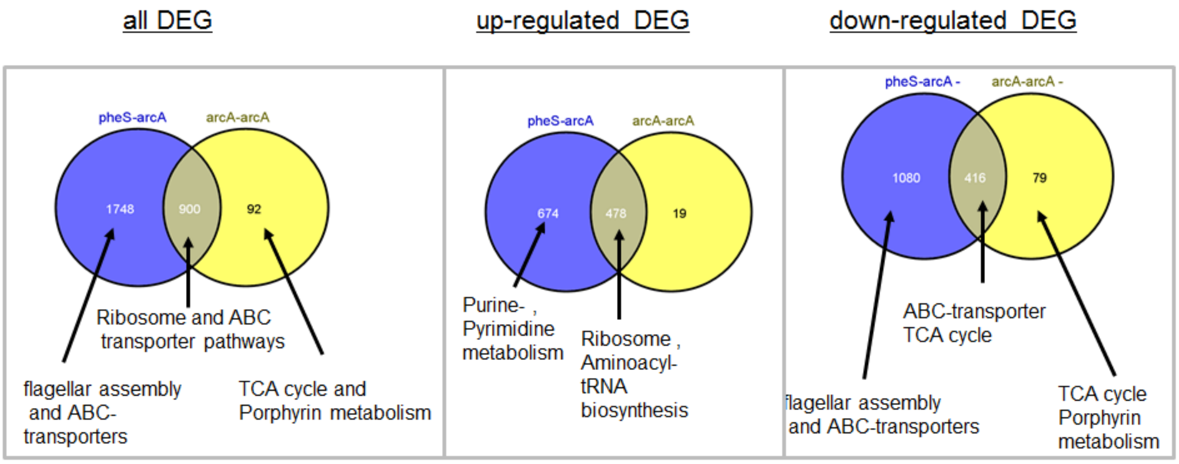


**Supplementary Figure 2. Comparison of the two constructs pheS-arcA and arcA-arcA to distill the effects of rewiring versus overexpression.** Systematic differences in differentially-expressed genes (DEG) between the constructs cannot be simply explained by overexpression of ArcA. The differences caused by the different promoter rewirings were analysed by functional KEGG pathway annotation clustering with the DAVID online tool[1, 2]. The 900 genes perturbed in both pheS-arcA and arcA-arcA are involved in the ribosome and ABC transporter pathways (Enrichment scores: 62.62; 62.23 and 11.03 respectively). By contrast, the genes that are differentially expressed only in pheS-arcA are enriched for flagellar assembly and ABC-transporters (scores 19.56; 16.96 and 15.09). Those only in arcA-arcA are involved in TCA cycle and Porphyrin metabolism (Enrichment scores 3.4 and 2.28). Upregulated genes only in pheS-arcA, are involved in Purine- and Pyrimidine metabolism (scores 12.62 and 8.94) Upregulated genes only in arcA-arcA show no annotated enrichment. Upregulated genes common to both are part of the ribosome and Aminoacyl-tRNA biosynthesis pathways (scores 75.15 and 9.98). The genes downregulated only in pheS-arcA are enriched for flagellar assembly (Enrichment score 22.7 and ABC transporters (score 14.49). Downregulated genes only in arcA-arcA show enrichment for Two-component systems (score 2.6) and porphyrin metabolism (score 2.3). Downregulated genes common to both are in the ABC-transporter pathway (score 17.7) and TCA cycle (score 6.78).

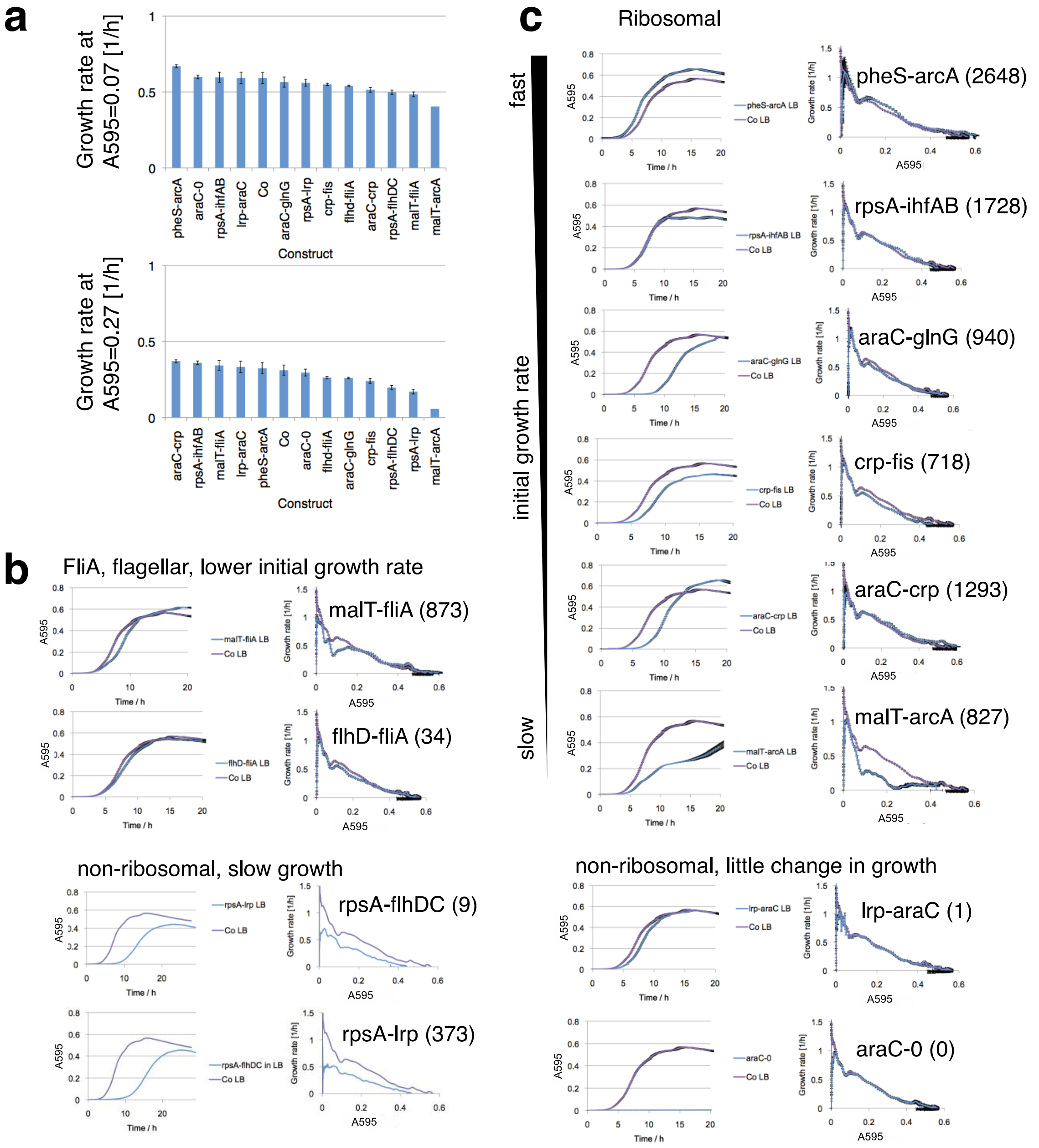


**Supplementary Figure 3. Growth timecourses and growth rates for selected rewired constructs.** 5 ml precultures were grown in LB medium for 16 hours, and diluting to an $OD_{600}$ of 0.0015 (around 1/800 dilution), in 120 □l LB medium on a 96-well plate. Absorbance was then measured on a platereader every 360 s, at 595 nm ($A_{595}$). Growth rates were calculated as the slope of the natural logarithm of $A_{595}$, with respect to time (a moving average over 6 consecutive readings). To aid comparison, growth rates were plotted against $A_{595}$. (**a**), Growth rates at $A_{595}$ = 0.07 (initial growth, as soon as can be detected by the platereader) and $A_{595}$ = 0.27 (midlog growth). (**b**), Growth timecourses (left) and growth rates versus $A_{595}$ (right) for selected constructs. The identity of each construct (together with number of differentially-expressed genes in brackets) is shown to the top-right of each dataset. The control construct (Co) is used as a reference throughout (purple). (**c**), Growth timecourses (left) and growth rates versus $A_{595}$ (right) for ribosome cluster constructs. Note the greater growth of pheS-arcA, relative to the control, Co (easiest to see on the $A_{595}$ growth curve). Other ribosomal constructs have various combinations of faster growth, slower growth, or delayed growth, relative to the control Co. Error bars are 1 s.e.m. for 10 replicates (colonies) throughout.

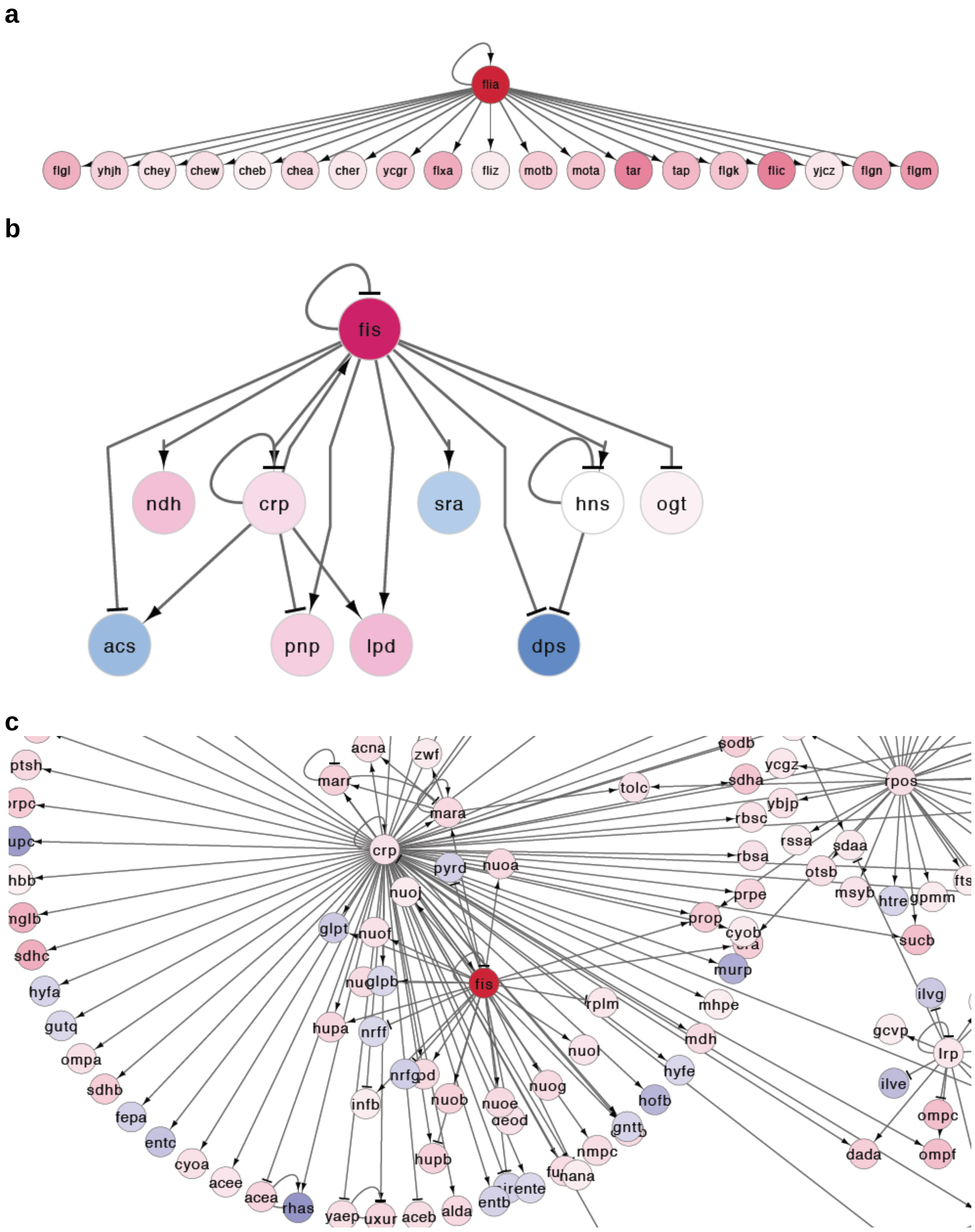


**Supplementary Figure 4. Network view of the perturbations caused by rewired constructs.** Colours: more red indicates more upregulation, more blue indicates more downregulation, relative to Co control cells. (**a**), Selected network view around *fliA* in flhD-fliA. Many directly-connected factors of FliA are upregulated (red circles), including genes involved in flagellar biosynthesis and chemotaxis. (**b**), Part of the network around the *fis* node, in the ribosomal clone rpoH-fis. (**c**), View of part of the (weakly ribosomal) pheS-fis network. Despite being implicated in regulating *rrn* P1 ribosomal gene promoters, most direct partners genes of *fis* are unchanged.

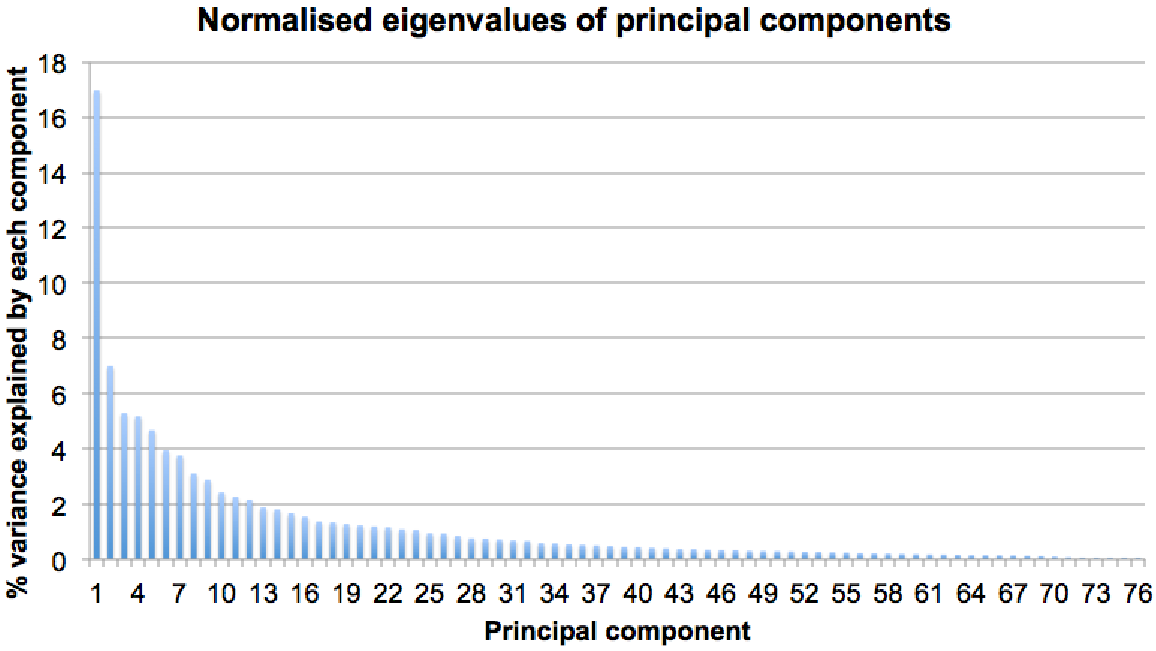


**Supplementary Figure 5. Principle component (PC) analysis of the 85 sample x 3891 gene matrix in Supplemental Data 1.** The normalized eigenvalues are shown. These represent the percentage of variance explained by each component. By selecting only PCs that explain at least as much variance as expected by chance (that is 100/80 % = 1.25%), the result is 21 PCs. Together these 21 PCs explain 77.6% of the variance. The gene and sample loadings per component are available from the authors on request.

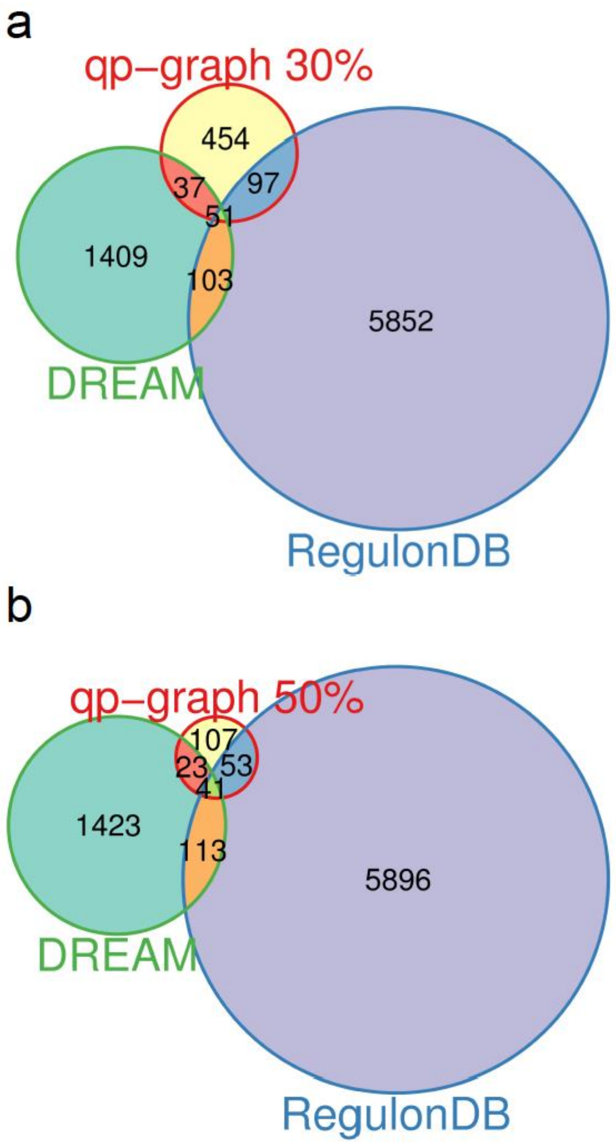


**Supplementary Figure 6. Comparison of qpgraph network inference in this study with the RegulonDB 7.2 database[3] and the Dialogue on Reverse Engineering Assessment and Methods (DREAM) project community prediction[4].** (**a**), Comparison of the reverse engineered graph in Figure 6 (qp-graph 30% precision) with the two public databases. 51 network interactions are common to all 3 databases. (**b**), A comparison of the 50% precision graph in Figure S7 (qp-graph 50%) with two public databases. 41 network interactions are common to all 3 databases.

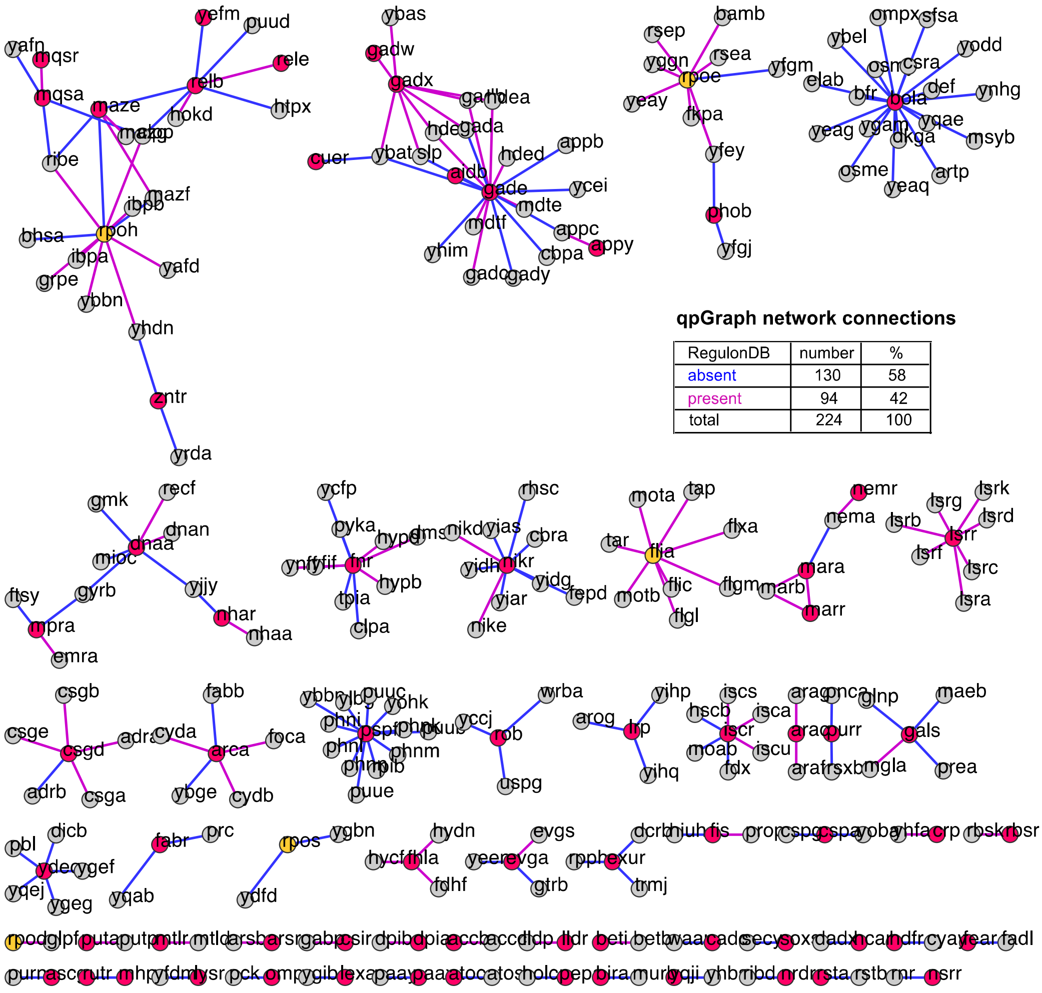

**qpGraph network connections**

| RegulonDB | number | % |
|---|---|---|
| absent | 130 | 58 |
| present | 94 | 42 |
| total | 224 | 100 |

**Supplementary Figure 7. Reversed-engineered network using qpgraph at 50% precision (0.5qpgraph).** Connected nodes are shown as circles (red=transcription factor (TF); yellow=□-factor; grey=regulated gene). Edges (direct predicted connections) are shown by lines (blue=absent in RegulonDB[3]; pink=present in RegulonDB). The inset table outlines the number of interactions recovered by qpgraph, divided into interactions that are present or absent in regulonDB.

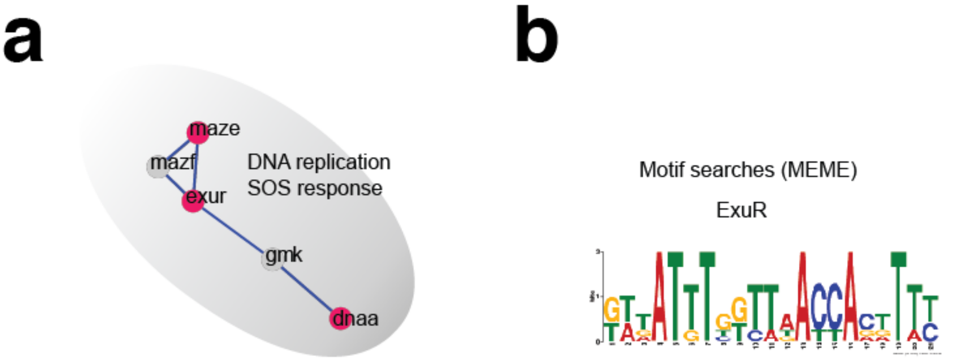


c

| Name | Strand | Start | *p*-value | Sites found by MEME | | | Regulon |
|---|---|---|---|---|---|---|---|
| uxuR | + | 326 | 1.09E-10 | CAATCAAACT | GATATTTGGTTGACCAGTTTT | CGTTTTTTTG | ✓ |
| exuR | - | 453 | 5.43E-10 | TACGGCAGAC | TATATTTGTTATACCACTTTT | GCGAGTTTAG | ✓ |
| uxuA | + | 319 | 7.99E-10 | TGAAATTCTT | GTGATGTGGTTAACCAATTTC | AGAATTCGGG | ✓ |
| exuT | - | 246 | 2.71E-09 | CACTTTGAGA | GTAATTTTTTTAACTACGTTT | ATTGATCTAA | ✓ |
| gmk | + | 469 | 1.35E-08 | GTATGTAGGC | TTTATTTCGCTAATCACATAC | GAAAGATACT | x |

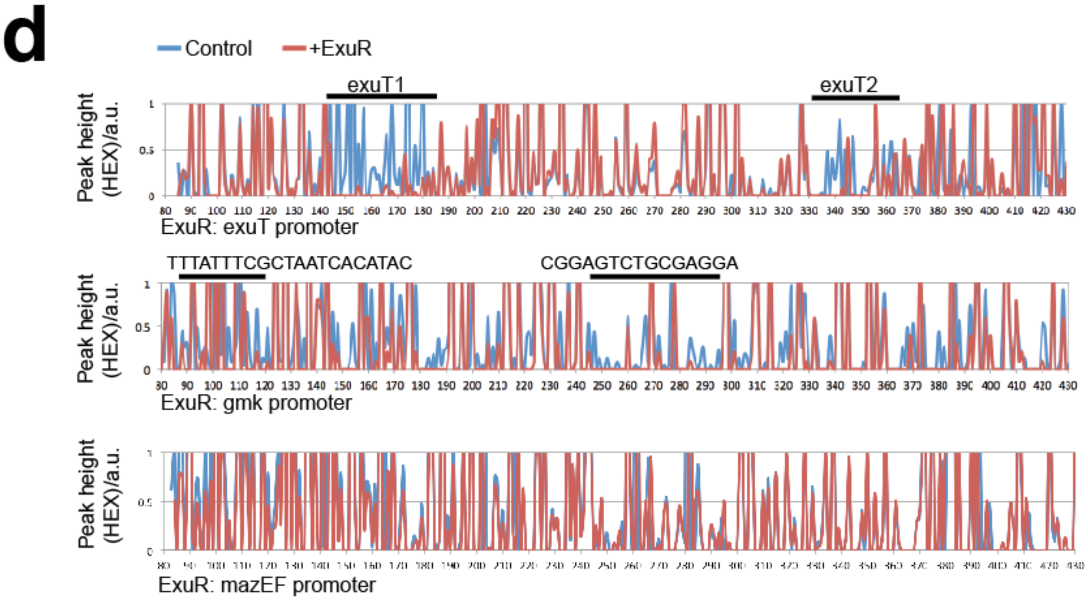


**Supplementary Figure 8. Testing new interactions in a subnetwork predicted by reverse engineering.** (**a**), The ExuR 50% precision subnetwork predicted by qpgraph contains potential new regulatory connections to *gmk* and *mazEF* that are absent in RegulonDB. Connected nodes are shown as circles (red=transcription factor (TF); grey=regulated gene). Edges (direct predicted connections) are shown by lines (blue=absent in RegulonDB[3]) (**b**), MEME[5] was used to predict a sequence logo for ExuR DNA binding. (**c**), MEME analysis of promoter regions described as being bound by ExuR in RegulonDB or in our qpgraph prediction. MEME finds putative ExuR sites on the *gmk* promoter region but not the *mazEF* promoter. (**d**), DNaseI footprinting analysis to test potential binding sites of ExuR. Sequences that are protected from DNaseI cleavage in the red ExuR sample, reveal the blue control traces. Footprint sites, and their approximate positions and sequences, are highlighted on the traces for the exuT and gmk promoters; no footprints are found in the mazEF promoter, in agreement with the MEME analysis. a.u., arbitrary units.

## Supplementary Tables

| ORF | ORF outdegree | mean transcription perturbations (TP) |
|---|---|---|
| arcA | 157 | 950 |
| fis | 222 | 456 |
| rpoD | 2135 | 1125 |
| ihfAB | 216 | 722 |
| crp | 446 | 459 |
| rpoN | 177 | 511 |
| rpoE | 543 | 350 |
| glnG | 44 | 494 |
| fecI | 5 | 208 |
| rpoH | 391 | 429 |
| appY | 10 | 343 |
| csgD | 22 | 639 |
| fnr | 286 | 443 |
| rpoS | 192 | 282 |
| lrp | 97 | 388 |
| hns | 166 | 108 |
| malT | 10 | 313 |
| fhlA | 29 | 140 |
| ompR | 17 | 37 |
| araC | 10 | 41 |
| fliA | 196 | 27 |
| flhDC | 80 | 10 |
| p-GFP | 0 | 143 |
| Linear regression | R square | 0.371 |
| | ANOVA F | 12.4 |
| | ANOVA P-level | 0.002 |

**Supplementary Table 1.** Linear regression to test correlation between ORF outdegree and mean transcription perturbations.

| | Promoter activity mesured by: | | |
|---|---|---|---|
| Promoter | Western blot | qRT-PCR | mean transcription perturbations (TP) |
| appY | 187299 | 1.2 | 159 |
| araC | 853058 | 3.09 | 380 |
| arcA | 400726 | 1.96 | 992 |
| crp | 411765 | 2.73 | 774 |
| csgD | 582385 | 1.55 | 323 |
| fecI | 246340 | 1.34 | 891 |
| flhD | 0 | 1.35 | 108 |
| fliA | 0 | 0.87 | 65 |
| hns | 932360 | 5.5 | 1256 |
| hypA | 0 | 0.73 | 215 |
| ihfB | 114120 | 2.56 | 398 |
| lrp | 107529 | 0.78 | 12 |
| malT | 437653 | 2.7 | 380 |
| nlpD | 0 | 0.54 | 33 |
| pheS | 68001 | 1.73 | 472 |
| rpoD | 64255 | 0.55 | 3 |
| rpoE | 19078 | 2.58 | 19 |
| rpoH | 374547 | 1.27 | 1034 |
| rpoS | 702063 | 2.25 | 114 |
| rpsA | 346422 | 4.59 | 703 |
| yhdG | 54502 | 1.05 | 1 |
| Linear regression | R square | 0.334 | 0.307 |
| | ANOVA F | 9.513 | 8.425 |
| | ANOVA P-level | 0.006 | 0.009 |

**Supplementary Table 2.** Linear regression to test correlation between promoter activity and mean transcription perturbations.

## Supplementary Methods

### Affymetrix E. coli Genome 2.0 microarrays

The 85 chosen gene network rewiring plasmids (from[6]) were transformed into *E. coli* TOP10 cells and grown under standardised conditions: bacteria were freshly plated onto LB Agar plates (supplemented with 100 µg $ml^{-1}$ Ampicillin and 50 µg $ml^{-1}$ Streptomycin) and incubated overnight at 37°C to form colonies. For each biological replicate, single colonies (maximum 3 days old) were used to inoculate separate 5 ml overnight pre-cultures in LB containing 100 µg $ml^{-1}$ Ampicillin and 50 µg $ml^{-1}$ Streptomycin. Constructs were grown for 37°C, at 220 rpm in an orbital shaker. The pre-cultures were diluted to an $OD_{600}$ of 0.0015 (approximately 1:800 dilution) in 2 ml of the same medium, in 14 ml culture tubes. Constructs were grown for 16h at 37°C, at 220 rpm in an orbital shaker. These conditions were chosen to match previous work[6].

Total bacterial RNA was extracted with a RNeasy Protect Bacteria Mini Kit (Qiagen, Cat. 74106). RNAprotect Bacteria Reagent (Qiagen, Cat. 76506) was added following the manufacturer's instructions (2 volumes reagent: 1 volume bacterial culture). RNA concentration was measured with a NanoDrop 2000 spectrophotometer. For microarray analysis, RNA integrity number was analysed with an Agilent 2100 Bioanalyzer. 10 µg of total bacterial RNA with a RNA integrity number > 7.0 was used for transcriptome analysis with Affymetrix GeneChip *E. coli* Genome 2.0 Arrays. MIAME compliant microarray data files are available at EBI ArrayExpress (ID: E-MTAB-3233). There are 255 raw array files for the 85 networks in biological triplicate samples (representing different colonies) and 5 files for the Co control[6].

### Microarray differential expression analysis

The microarrays probed for the relative RNA expression levels of 3891 annotated *E. coli* genes with unique Entrez IDs. All network constructs (biological triplicates from different colonies) were compared to 5 biological replicates of a "wild-type" reference standard (*E. coli* transformed with an empty promoterless GFP plasmid; denoted "Co"[6]). Data analysis was performed using the R programming language and the Bioconductor software packages. Each Affymetrix chip was background adjusted, normalized and log2 transformed using the Robust Multichip Averaging (RMA) algorithm[7]. Differential expression analysis was performed by using the Bioconductor package limma[8]. The genes which were differentially expressed (<5% False Discovery Rate[9], >1.2-fold-change) were extracted and used to analyse the scale of network perturbations.

EPCLUST (http://www.bioinf.ebc.ee/EP/EP/EPCLUST/)[10] was used for clustering the array data of fold-changes in mRNA levels. The chosen options for clustering provided on the webserver are annotated in Supplemental Data 1.

### pBAD arabinose-inducible constructs

ORFS were obtained via PCR, from 15 ng *E. coli* TOP10 genomic DNA, and were cloned using the pBAD202 Directional TOPO Expression Kit (Invitrogen, Cat. K4202-01). After sequence verification, positive clones in TOP10 cells were grown in LB medium, supplemented with 30 µg $ml^{-1}$ Kanamycin and 0.002% arabinose, to induce the pBAD promoter. RNA was extracted as described above.

*Quantitative real-time PCR (qRT-PCR)*

*E. coli* TOP10 cells containing the different promoter-gene constructs were grown for RNA extraction as described above, except that they were grown in 500 µl LB

(ribosome experiments; Figure 4), in 2.2 ml 96 well culture plates (PEQLAB Cat. 82-0932-A). The plates were sealed with Breathe-Easy™ sealing membrane (Sigma, Cat. Z380059).

Reverse transcription (pBAD clones) was carried out with SuperScript II Reverse Transcriptase (Invitrogen, Cat. 18064-014**)**, according to the manufacturer's instructions (total reaction volume of 20 µl; 500 ng of RNA; Oligo(dT)12–18 Primer (Invitrogen, Cat. 18418-012)). Samples were diluted 1:10 in water. 2 µl of the cDNA sample were used per RT-qPCR reaction. Reverse transcription for arabinose and ribosome state experiments was similar except for using the QuantiTect Reverse Transcription Kit (QIAGEN, Cat. 205314; genomic DNA wipe out buffer and mixed oligoDT-random primers).

RNA transcripts were quantified in a 10 µl RT-qPCR reaction, containing 2 µl of the cDNA sample, 0.25 µM final concentration of forward and reverse primers and 5 µl of LightCycler® 480 SYBR Green I Master (Roche, Cat. 04 707 516 001). RT-qPCR reactions were run on a LightCycler 480 System (Roche) in 384 well plates: (95˚C, 8 min, 4.8˚C $s^{-1}$; [95˚C, 10 s, 4.8˚C $s^{-1}$; 55˚C, 30 s, 2.5˚C $s^{-1}$; 72˚C, 10 s, 4.8˚C $s^{-1}$] x 40 cycles; [95˚C, 30 s, 4.8˚C $s^{-1}$; 65˚C, 10 min, 2.5˚C $s^{-1}$; 98˚C, 10 s, 0.11˚C $s^{-1}$]; [55˚C, 10 s, 0.11˚C $s^{-1}$; 72˚C, 2 min, 4.8˚C $s^{-1}$]). All primer sets were tested for specificity by melting curve analysis standard curves with *E. coli* genomic DNA. All samples were normalized against levels of *gnd* (a housekeeping gene) in the same sample. Fold-change of expression over the Co control sample was calculated by dividing the *gnd*-normalized concentration of the sample by the *gnd*-normalized concentration of the Co sample.

## Statistics

Linear regression correlation analysis was carried out on microarray data with StatPlus:mac.LE.2009 ANOVA F test to calculate p-values. 95% confidence intervals of growth curves (fluorescence and A595) were estimated using ± 1.96 × standard error mean for each time point.

## Biclustering

Biclustering was implemented by using the Iterative Signature Algorithm (ISA)[11], as implemented in the R Bioconductor *eisa* package (v. 1.4.1)[12]. The gene (features) and sample thresholds were set to 2.1 and 1.5, respectively. Gene filter cutoffs: genes with log2 fold-changes >0.25 or <-0.25 in at least three points were used.

## Gene Ontology Enrichment Analysis

Gene ontology[13] analysis was performed using R Bioconductor package GOstats (v. 2.18)[13]. Affymetrix IDs were converted into Entrez Gene identifiers and then mapped against the GO database (R Bioconductor annotation packages ecoli2.db and GO.db). All tests were done on the Biological Process (BP), Molecular Functions (MF) and Cellular Components (CC) ontology. Enrichment analysis used hypergeometric-based tests with a 'universe' of 4070 annotated MG1655 E. coli genes. p-value threshold was set to 0.01 and significance was determined with the FDR-adjusted p-value cutoff at 0.05[9].

## Reverse-engineering with qpgraph

The Bioconductor package qpgraph[14] was used to estimate the structure of the network from the 255 microarrays. Non-rejection rates (NRR) were calculated, which are based on partial correlations of order $q < (n-2)$[15]. The NRR gives an estimate of the strength of a direct interaction between two genes and can be understood as a linear measure of association over all marginal distributions of size q that is calculated for every gene pair. We calculated NRRs for every possible value of q and calculated an average NRR[14] which was used to rank all regulator-target associations and to estimate precision-recall curves

based on RegulonDB 7[3]. 30%-precision (% number of true positives per number of predicted edges whose genes belong to at least 1 RegulonDB interaction) was selected to generate the full network .

## Network visualization

Network visualization was done with Cytoscape Version 2.8.2[16]. To calculate topological parameters of the network we used the Cytoscape plug-in NetworkAnalyzer v2.7[17]. ClueGo v1.4 plug-in was used for gene set enrichment analysis with GO Biological process annotations[18].

## De-novo detection of overrepresented motifs

Upstream DNA regions of co-expressed genes were obtained from Ecogene 3.0[19] and the MEME online Motif Search tool was used to screen the -1000 upstream regions (-1000bp from transcription start sites) for overrepresented motifs[5].

## In vitro transcription-translation and DNaseI footprinting

The ORF for *exuR* was PCR amplified from *E. coli* Top10 genomic DNA with KOD Hot Start Polymerase (Novagen) and was cloned via NdeI-BamHI into the control plasmid from the PURExpress *in vitro* protein synthesis kit (NEB)(primers: GCGCGCCATATGGAAATCACTGAA; CCCGGGATCCTCATCATTTACTGCCGCT). The -1000 upstream regions was also obtained by genomic PCR, with primers containing 5'-complementary sequence to universal fluorescent (HEX) labelled primers, for generating fluorescent end-labelled promoter fragments. Primer sets for DnaseI footprinting:

```
>EG12738 exuT Hexuronate permease, for glucuronate and galacturonate
TCGAGGTCGACGGTATCGATCAGTCATAAACGGAGTCAT
TGACGAGTAGACGCTGGTAGTTTCGTGCTAATTCGGCT
>EG10965 gmk Guanylate kinase
TCGAGGTCGACGGTATCGATGACAAAAACGCCCAGAGA
TGACGAGTAGACGCTGGTAGATAAAGCCTACATACGAGC
>EG10571 mazE MazE antitoxin, toxin is MazF
TCGAGGTCGACGGTATCGATAAACAGGACCGTGACAAAA
TGACGAGTAGACGCTGGTAGCATTAACGTAGCCGGGAT
>universal_footprint_forward_FAM
TCGAGGTCGACGGTATCGAT
>universal_footprint_reverse_HEX
TGACGAGTAGACGCTGGTAG
```

ExuR plasmid or negative control plasmid was added to the protein synthesis kit and expression was verified by SDS-PAGE. After expression the reactions were incubated with 1 µg poly-dIdC (Sigma-Aldrich #81349) (on ice, 10 min), labelled probe (37˚C, 20 min) and 0.05-0.1 units DNaseI (26˚C, 5 min) in Valmeekam ExuR Binding Buffer: 7.5 mM Tris-HCl pH 7.4, 0.5 mM EDTA, 5 mM KCl, 2.5% glycerol, 0.5 mM DTT, 0.4 µg BSA. Samples were heat inactivated (75˚C, 10 min), column purified, and mixed 1:6 with HiDi Formamide and 0.4 µl ROX 1000 GeneScan size standard. Fragment analysis was performed on a GeneScan ABI3130xl DNA analyzer. Samples were analysed with GeneMapper Version 4.0. Sequences were obtained in parallel with Thermo Sequenase Dye Primer Manual Cycle Sequencing Kit. Sequences and fragments were aligned using the ROX 1000 internal size standard.

**Supplementary References**


1. Huang da W, Sherman BT, Lempicki RA. Systematic and integrative analysis of large gene lists using DAVID bioinformatics resources. *Nature protocols* **4**, 44-57 (2009).

2. Huang da W, Sherman BT, Lempicki RA. Bioinformatics enrichment tools: paths toward the comprehensive functional analysis of large gene lists. *Nucleic Acids Res* **37**, 1-13 (2009).

3. Gama-Castro S*, et al.* RegulonDB version 7.0: transcriptional regulation of Escherichia coli K-12 integrated within genetic sensory response units (Gensor Units). *Nucleic Acids Res* **39**, D98-105 (2011).

4. Marbach D*, et al.* Wisdom of crowds for robust gene network inference. *Nat Methods* **9**, 796-804 (2012).

5. Bailey TL*, et al.* MEME SUITE: tools for motif discovery and searching. *Nucleic Acids Res* **37**, W202-208 (2009).

6. Isalan M*, et al.* Evolvability and hierarchy in rewired bacterial gene networks. *Nature* **452**, 840-845 (2008).

7. Irizarry RA*, et al.* Exploration, normalization, and summaries of high density oligonucleotide array probe level data. *Biostatistics* **4**, 249-264 (2003).

8. Smyth GK. Linear models and empirical bayes methods for assessing differential expression in microarray experiments. *Stat Appl Genet Mol Biol* **3**, Article3 (2004).

9. Benjamini Y, Hochberg Y. Controlling the false discovery rate: a practical and powerful approach to multiple testing. *J Roy Statist Soc Ser B* **57**, 289-300 (1995).

10. Vilo J, Kapushesky M, Kemmeren P, Sarkans U, Brazma A. *The Analysis of Gene Expression Data: Methods and Software*. Springer Verlag (2003).

11. Bergmann S, Ihmels J, Barkai N. Iterative signature algorithm for the analysis of large-scale gene expression data. *Phys Rev E Stat Nonlin Soft Matter Phys* **67**, 031902 (2003).

12. Gentleman RC*, et al.* Bioconductor: open software development for computational biology and bioinformatics. *Genome Biol* **5**, R80 (2004).

13. Falcon S, Gentleman R. Using GOstats to test gene lists for GO term association. *Bioinformatics* **23**, 257-258 (2007).

14. Castelo R, Roverato A. Reverse engineering molecular regulatory networks from microarray data with qp-graphs. *J Comput Biol* **16**, 213-227 (2009).

15. Castelo R, Roverato A. A Robust Procedure For Gaussian Graphical Model Search From Microarray DataWith p Larger Than n. *Journal of Machine Learning Research* **7**, 2621-2650 (2006).

16. Smoot ME, Ono K, Ruscheinski J, Wang PL, Ideker T. Cytoscape 2.8: new features for data integration and network visualization. *Bioinformatics* **27**, 431-432 (2011).

17. Assenov Y, Ramirez F, Schelhorn SE, Lengauer T, Albrecht M. Computing topological parameters of biological networks. *Bioinformatics* **24**, 282-284 (2008).

18. Bindea G, *et al.* ClueGO: a Cytoscape plug-in to decipher functionally grouped gene ontology and pathway annotation networks. *Bioinformatics* **25**, 1091-1093 (2009).

19. Zhou J, Rudd KE. EcoGene 3.0. *Nucleic Acids Res* **41**, D613-624 (2013).
