# Supplementary material for: The propagation of perturbations in rewired bacterial gene networks
Source: Nat Commun. 2015 Dec 16;6:10105. doi: 10.1038/ncomms10105 (PMC4703840; doi:10.1038/ncomms10105)
Supplement: Supplementary Data 2 — Biclusters of gene expression for 85 rewired gene networks. The rewired constructs are listed along the bottom of each heatmap (individual bicluster) and the genes in the subcluster are listed to the right. A color key of the log2 fold change of each sample is provided for each heatmap. A Gene ontology Enrichment analysis of each bicluster is provided to the left of each heatmap (where statistically significant). [file ncomms10105-s3.pdf]

Supplementary Data 2 - Biclusters

Each of the following pages contains one heatmap for one bicluster. The rewired constructs are listed along the bottom of the heatmaps and the genes in the subcluster are listed to the right. A color key of the log2 fold change of each sample is provided for each heatmap. A Gene ontology Enrichment analysis of each bicluster is provided to the left of each heatmap (where statistically significant).

Summary

Subcluster 1 - carbohydrate
Subcluster 2 - starvation-respiration
Subcluster 3 - stress downregulation
Subcluster 4 - metabolic
Subcluster 5 - peptidoglycan - cell wall
Subcluster 6 - polysaccharide
Subcluster 7 - flagellum - fliA
Subcluster 8 - -rpoE
Subcluster 9 - -fis/-fecI
Subcluster 10 - hypA-
Subcluster 11 - stress-carbohydrate
Subcluster 12 - biosynthesis-catabolic
Subcluster 13 - RNA-nucleotide
Subcluster 14 - metabolic
Subcluster 15 - flagellum - flhDC
Subcluster 16 - pmrD
Subcluster 17 - stress-carbohydrate
Subcluster 18 - catabolic-carbohydrate
Subcluster 19 - lipopolysaccharide
Subcluster 20 - ribosomal-ATP
Subcluster 21 - htpX
Subcluster 22 - purine biosynthesis
Subcluster 23 - DNA metabolism-recombination

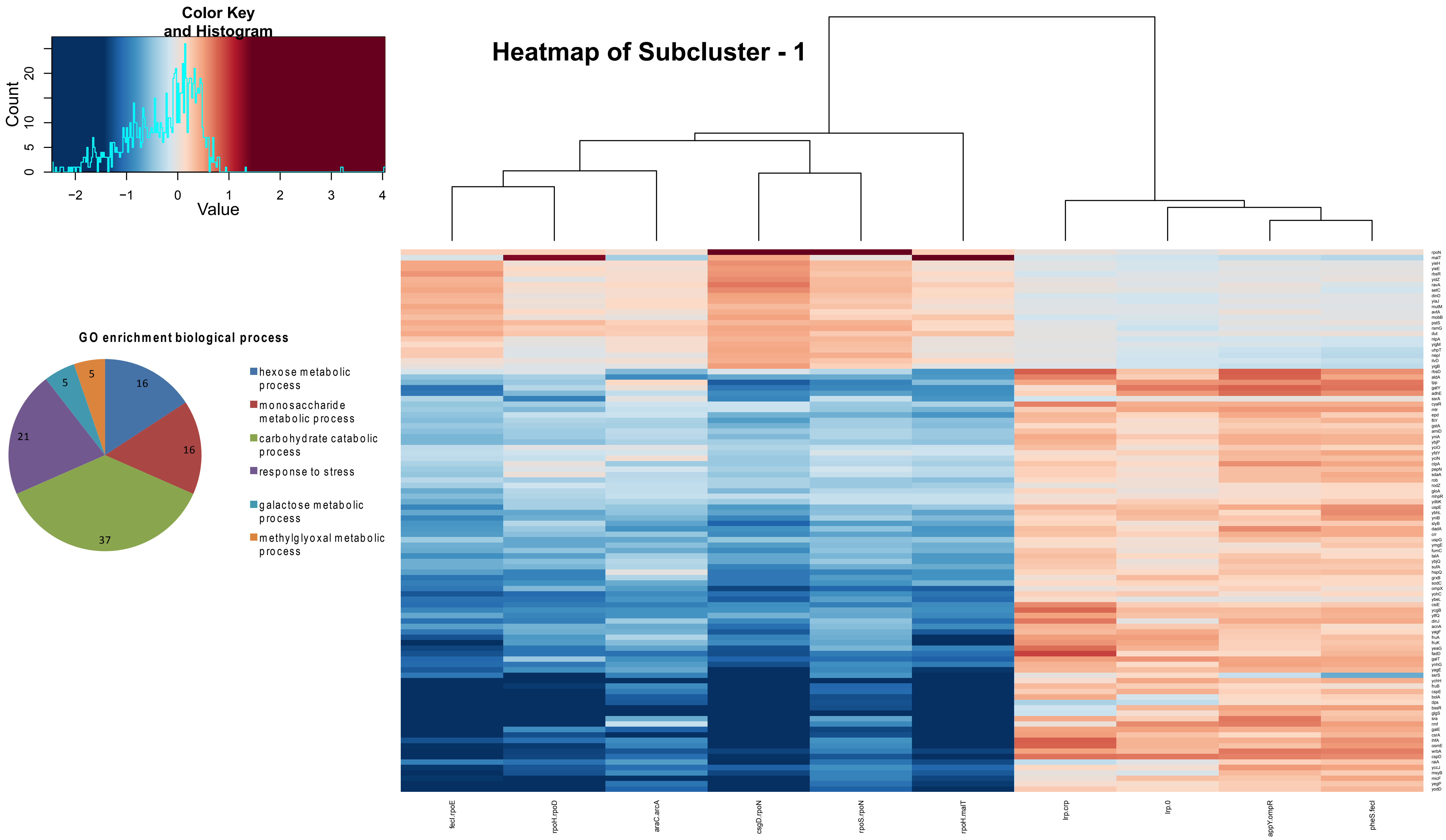
Color Key
and Histogram
Count
Value
Heatmap of Subcluster - 1
GO enrichment biological process
hexose metabolic process
monosaccharide metabolic process
carbohydrate catabolic process
response to stress
galactose metabolic process
methylglyoxal metabolic process
16
16
37
21
5
5
fecI.rpoE
rpoH.rpoD
araC.arcA
csgD.rpoN
rpoS.rpoN
rpoH.malT
lrp.crp
lrp.0
appY.ompR
pheS.fecI

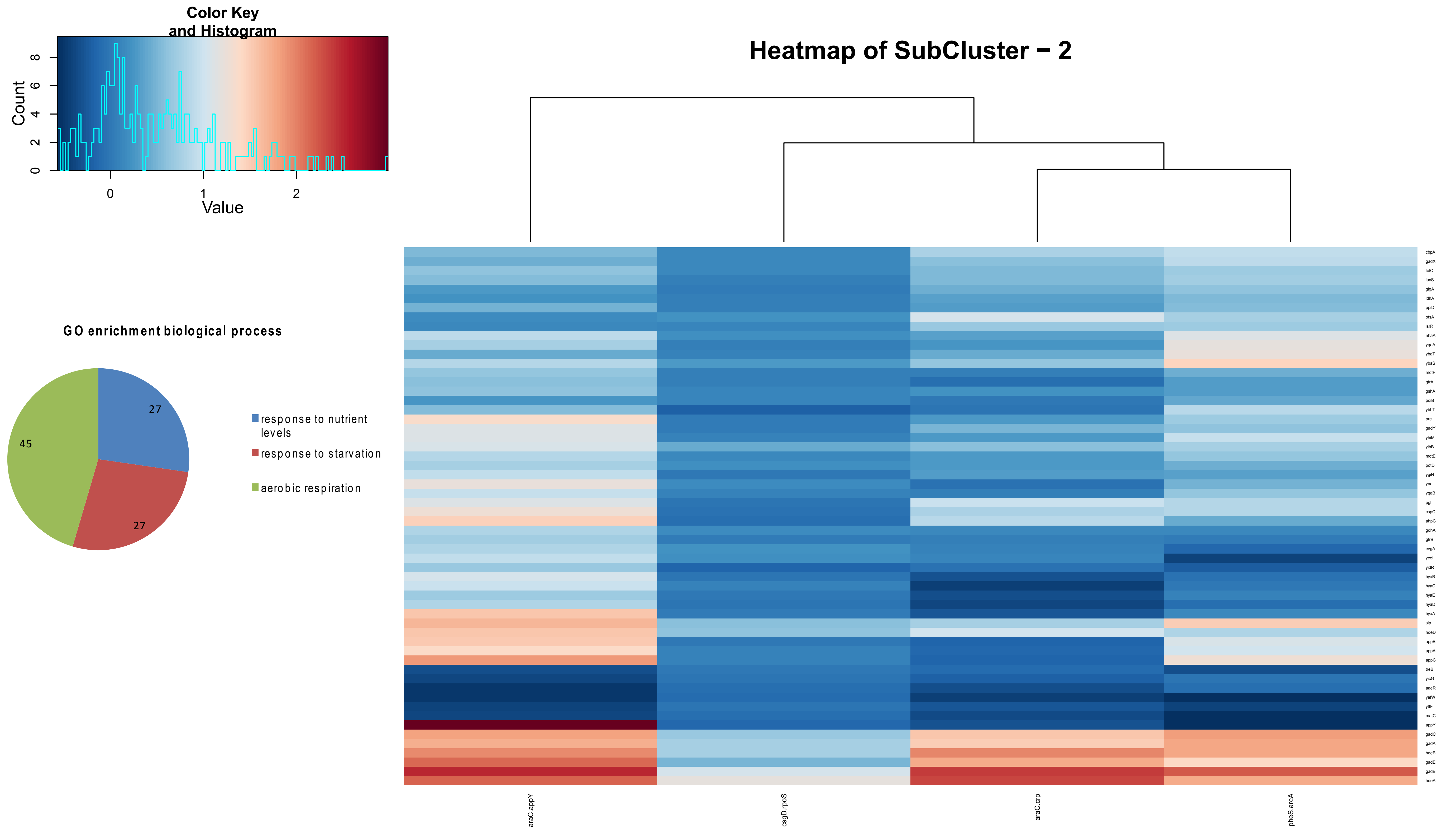
Color Key
and Histogram
Count
0
2
4
6
8
0
1
2
Value
Heatmap of SubCluster − 2
cbpA
gadX
tolC
luxS
glgA
ldhA
ppiD
otsA
lsrR
nhaA
yqaA
ybaT
ybaS
mdtF
gtrA
gshA
pqiB
ybhT
prc
gadY
yhiM
yibB
mdtE
potD
ygiN
ynaI
yqaB
pgl
cspC
ahpC
gdhA
gtrB
evgA
yceI
yidR
hyaB
hyaC
hyaE
hyaD
hyaA
slp
hdeD
appB
appA
appC
treB
yicG
aaeR
yafW
ytfF
matC
appY
gadC
gadA
hdeB
gadE
gadB
hdeA
araC.appY
csgD.rpoS
araC.crp
pheS.arcA
GO enrichment biological process
27
27
45
response to nutrient levels
response to starvation
aerobic respiration

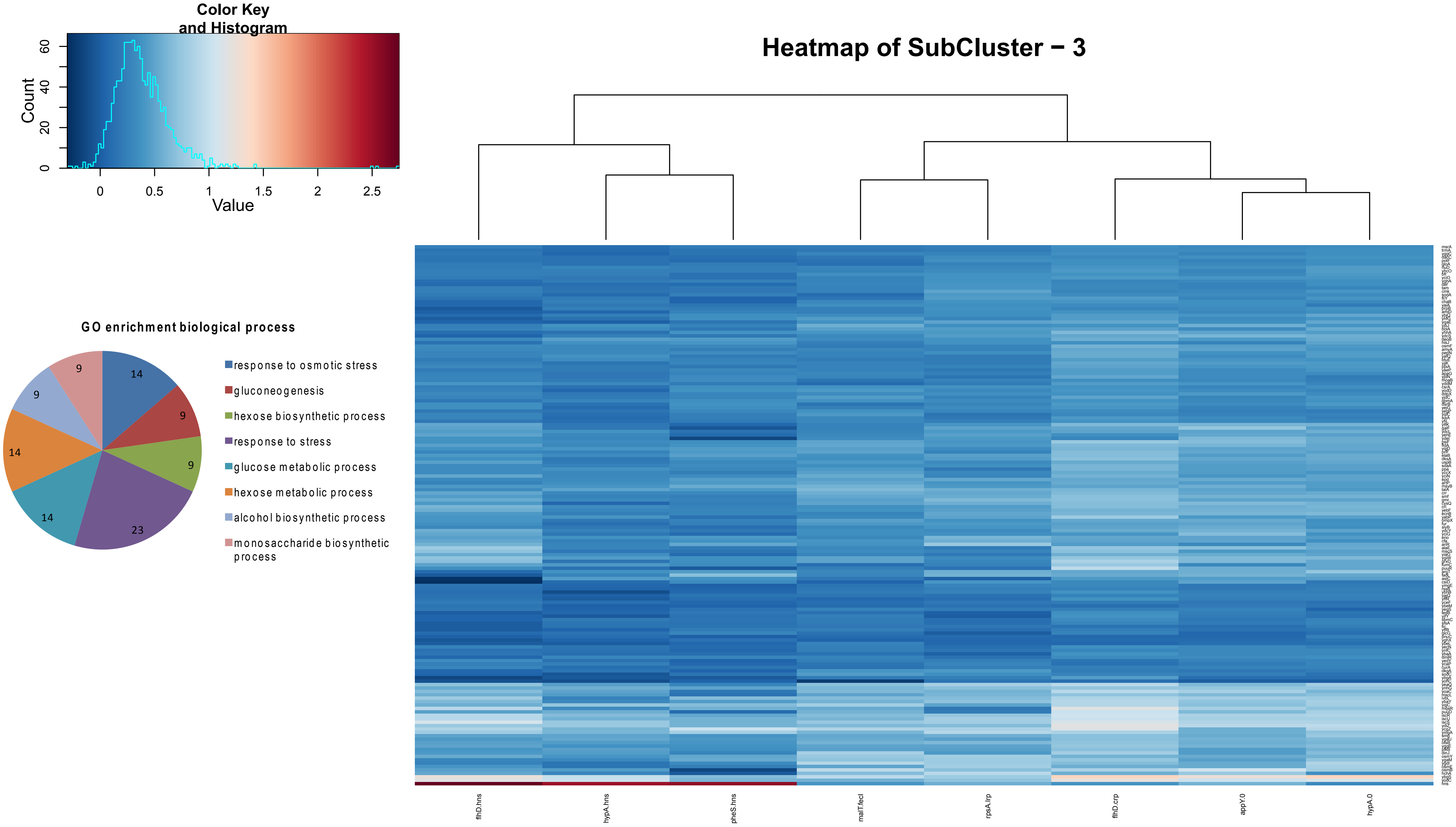

Color Key
and Histogram
Count
Value
0
20
40
60
0
0.5
1
1.5
2
2.5
Heatmap of SubCluster − 3
GO enrichment biological process
response to osmotic stress
gluconeogenesis
hexose biosynthetic process
response to stress
glucose metabolic process
hexose metabolic process
alcohol biosynthetic process
monosaccharide biosynthetic process
14
9
9
23
14
14
9
9
flhD.hns
hypA.hns
pheS.hns
malT.fecI
rpsA.lrp
flhD.crp
appY.0
hypA.0

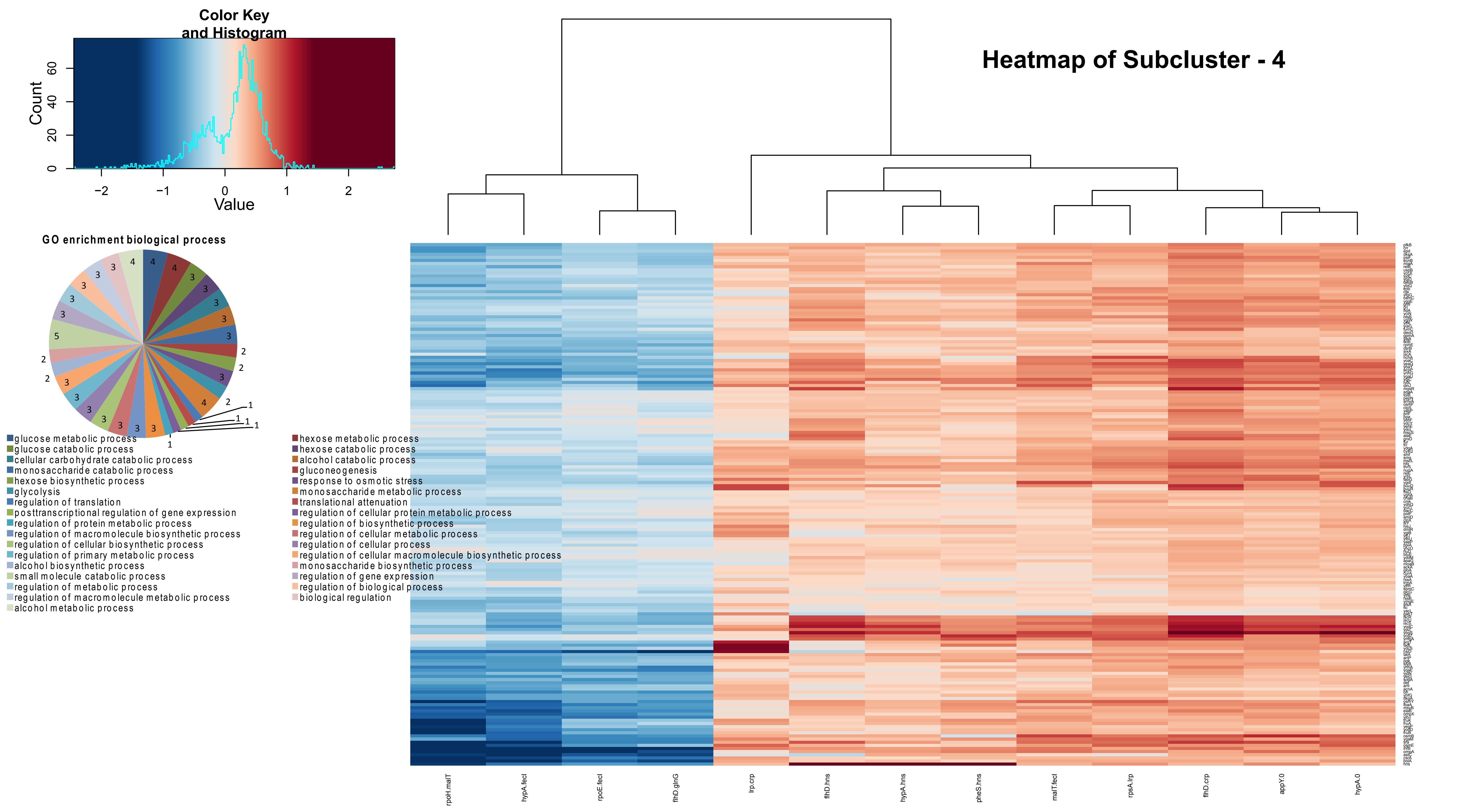
Color Key and Histogram
Count
Value
Heatmap of Subcluster - 4
GO enrichment biological process
glucose metabolic process
glucose catabolic process
cellular carbohydrate catabolic process
monosaccharide catabolic process
hexose biosynthetic process
glycolysis
regulation of translation
posttranscriptional regulation of gene expression
regulation of protein metabolic process
regulation of macromolecule biosynthetic process
regulation of cellular biosynthetic process
regulation of primary metabolic process
alcohol biosynthetic process
small molecule catabolic process
regulation of metabolic process
regulation of macromolecule metabolic process
alcohol metabolic process
hexose metabolic process
hexose catabolic process
alcohol catabolic process
gluconeogenesis
response to osmotic stress
monosaccharide metabolic process
translational attenuation
regulation of cellular protein metabolic process
regulation of biosynthetic process
regulation of cellular metabolic process
regulation of cellular process
regulation of cellular macromolecule biosynthetic process
monosaccharide biosynthetic process
regulation of gene expression
regulation of biological process
biological regulation
rpoH.malT
hypA.fecI
rpoE.fecI
flhD.glnG
lrp.crp
flhD.hns
hypA.hns
pheS.hns
malT.fecI
rpsA.lrp
flhD.crp
appY.0
hypA.0

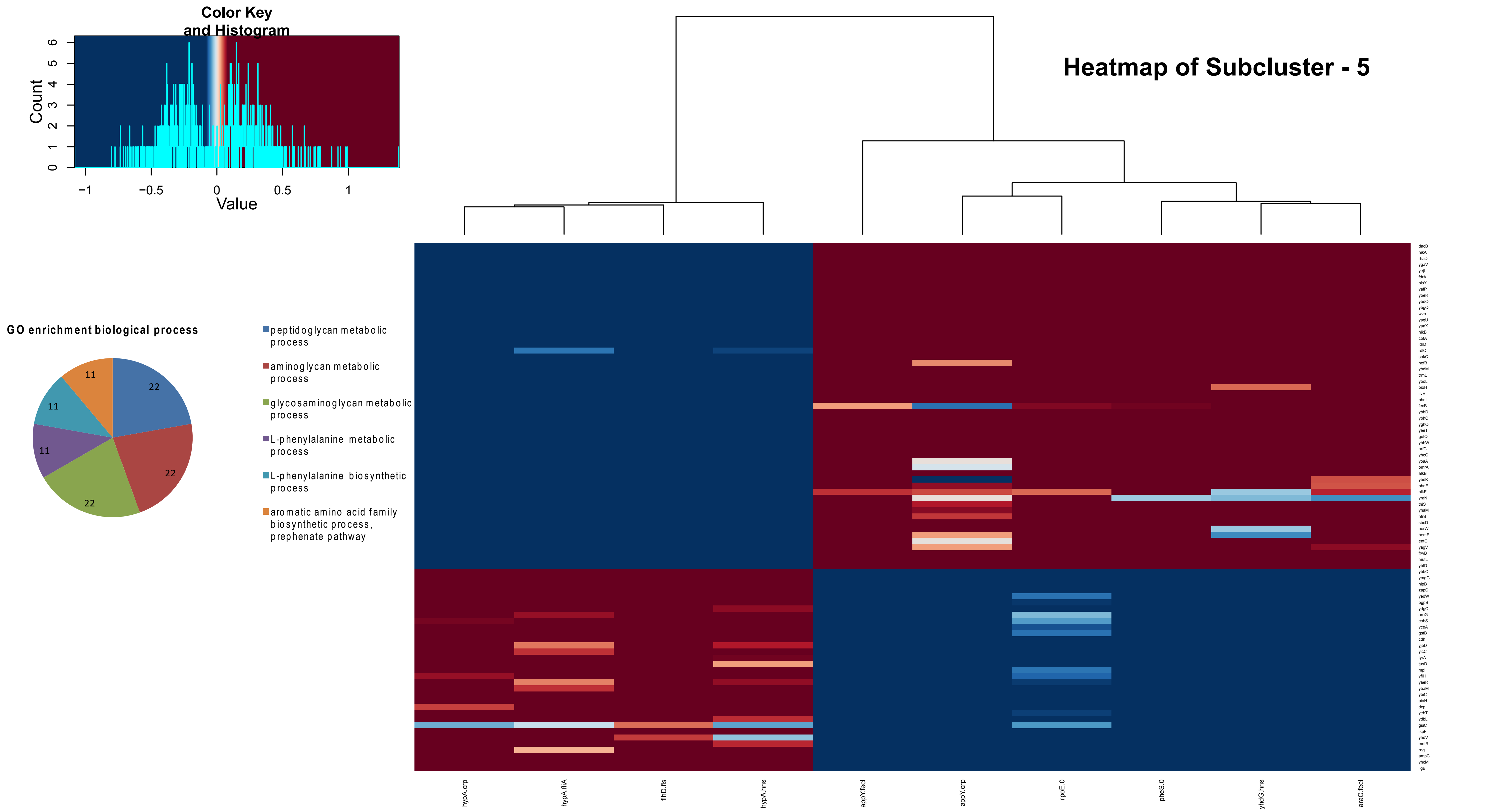

Color Key and Histogram
Count
Value
−1
−0.5
0
0.5
1
0
1
2
3
4
5
6
Heatmap of Subcluster - 5
hypA.crp
hypA.fliA
flhD.fis
hypA.hns
appY.fecI
appY.crp
rpoE.0
pheS.0
yhdG.hns
araC.fecI
GO enrichment biological process
22
22
22
11
11
11
peptidoglycan metabolic process
aminoglycan metabolic process
glycosaminoglycan metabolic process
L-phenylalanine metabolic process
L-phenylalanine biosynthetic process
aromatic amino acid family biosynthetic process, prephenate pathway

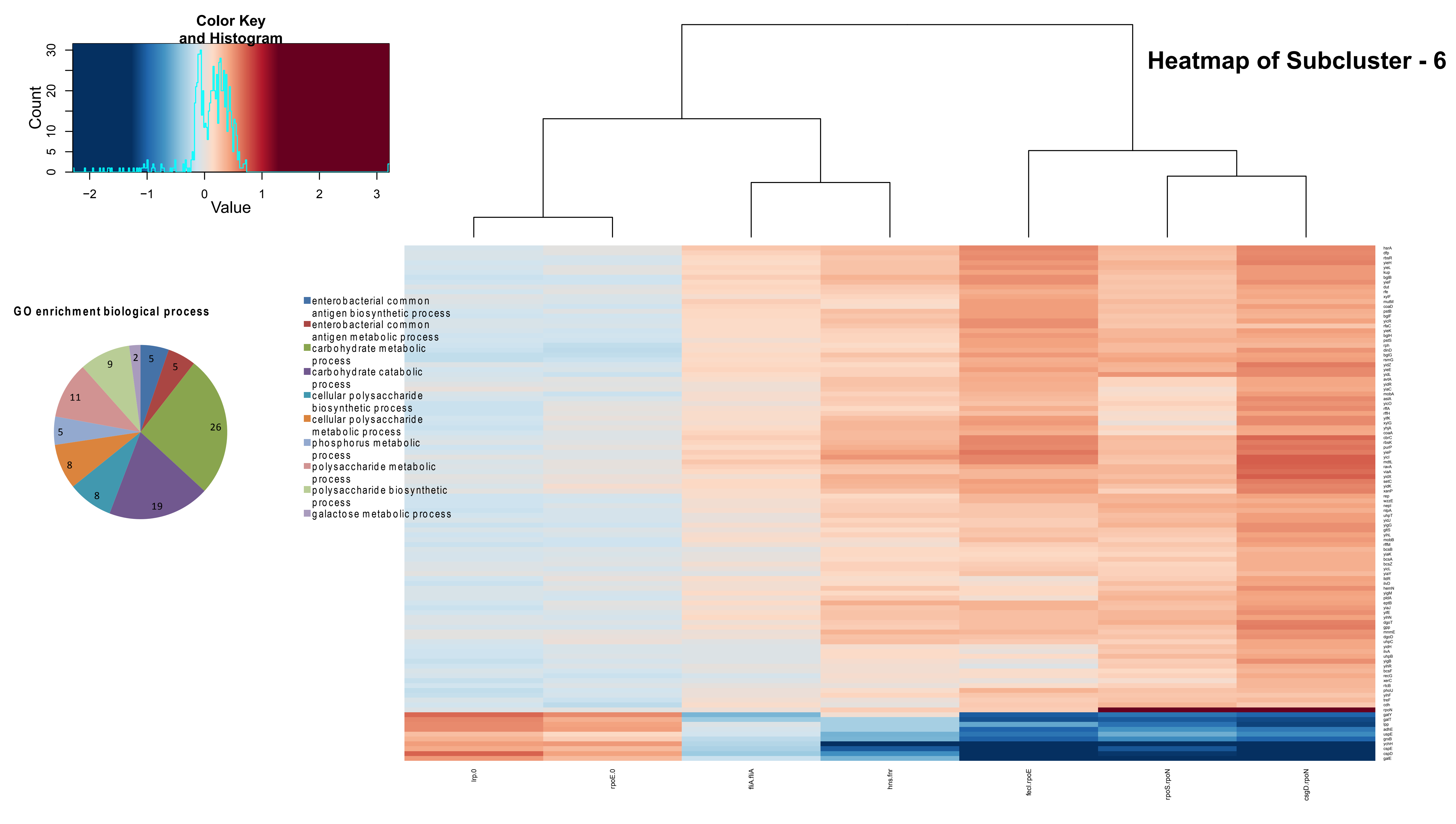

Heatmap of Subcluster - 6
Color Key and Histogram
Count
Value
GO enrichment biological process
enterobacterial common antigen biosynthetic process
enterobacterial common antigen metabolic process
carbohydrate metabolic process
carbohydrate catabolic process
cellular polysaccharide biosynthetic process
cellular polysaccharide metabolic process
phosphorus metabolic process
polysaccharide metabolic process
polysaccharide biosynthetic process
galactose metabolic process
lrp.0
rpoE.0
fliA.fliA
hns.fnr
fecI.rpoE
rpoS.rpoN
csgD.rpoN

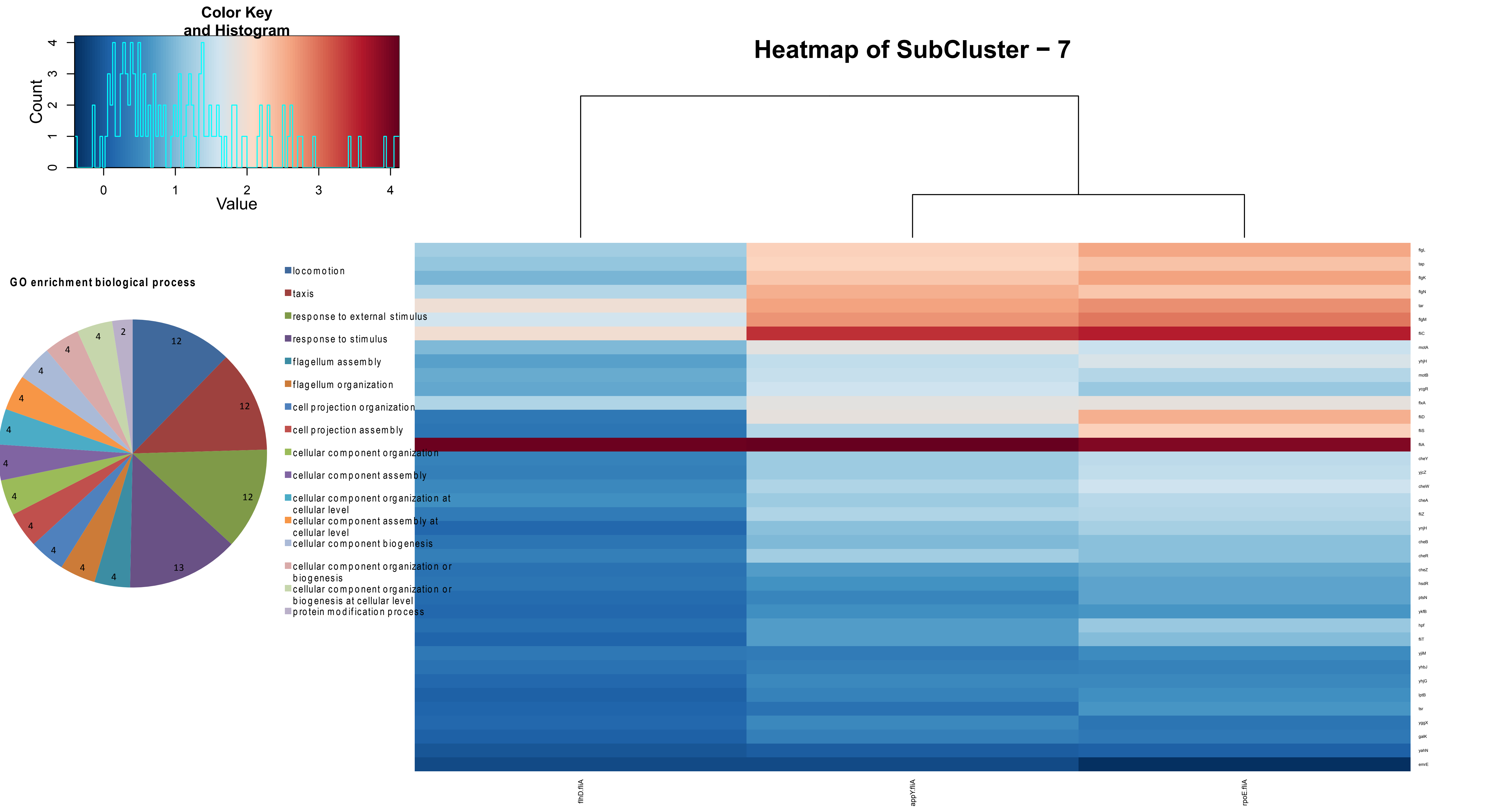

Color Key
and Histogram
Count
Value
0
1
2
3
4
Heatmap of SubCluster − 7
GO enrichment biological process
locomotion
taxis
response to external stimulus
response to stimulus
flagellum assembly
flagellum organization
cell projection organization
cell projection assembly
cellular component organization
cellular component assembly
cellular component organization at cellular level
cellular component assembly at cellular level
cellular component biogenesis
cellular component organization or biogenesis
cellular component organization or biogenesis at cellular level
protein modification process
12
12
12
13
4
4
4
4
4
4
4
4
4
4
2
flgL
tap
flgK
flgN
tar
flgM
fliC
motA
yhjH
motB
ycgR
flxA
fliD
fliS
fliA
cheY
yjcZ
cheW
cheA
fliZ
ynjH
cheB
cheR
cheZ
hsdR
ptsN
ykfB
hpf
fliT
yjiM
yhbJ
yhjG
lptB
tsr
yggX
galK
yahN
emrE
flhD.fliA
appY.fliA
rpoE.fliA

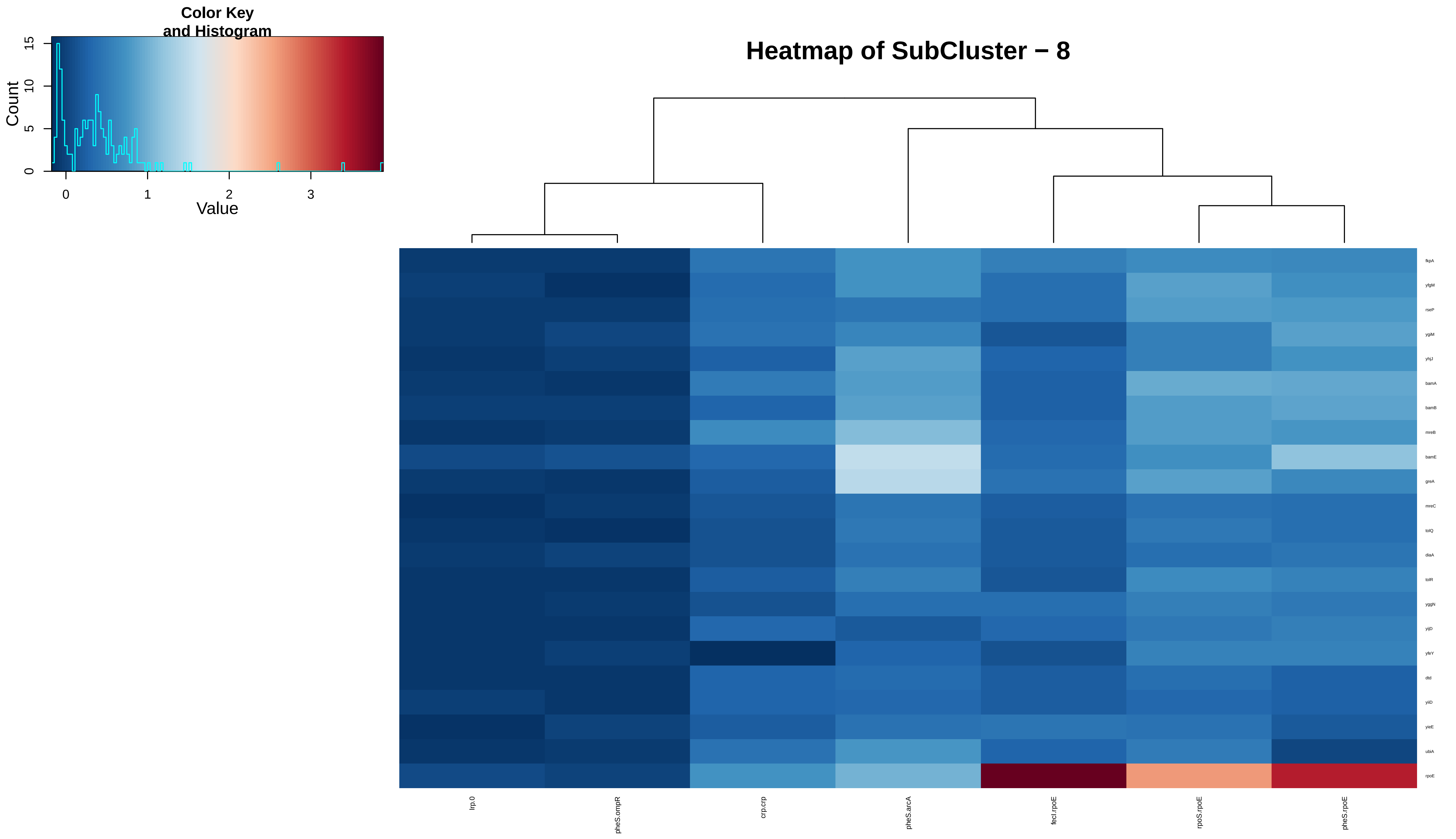
Color Key
and Histogram
Count
Value
0
5
10
15
0
1
2
3
Heatmap of SubCluster − 8
fkpA
yfgM
rseP
ygiM
yhjJ
bamA
bamB
mreB
bamE
greA
mreC
tolQ
diaA
tolR
yggN
yijD
yfeY
dtd
yiiD
yieE
ubiA
rpoE
lrp.0
pheS.ompR
crp.crp
pheS.arcA
fecI.rpoE
rpoS.rpoE
pheS.rpoE

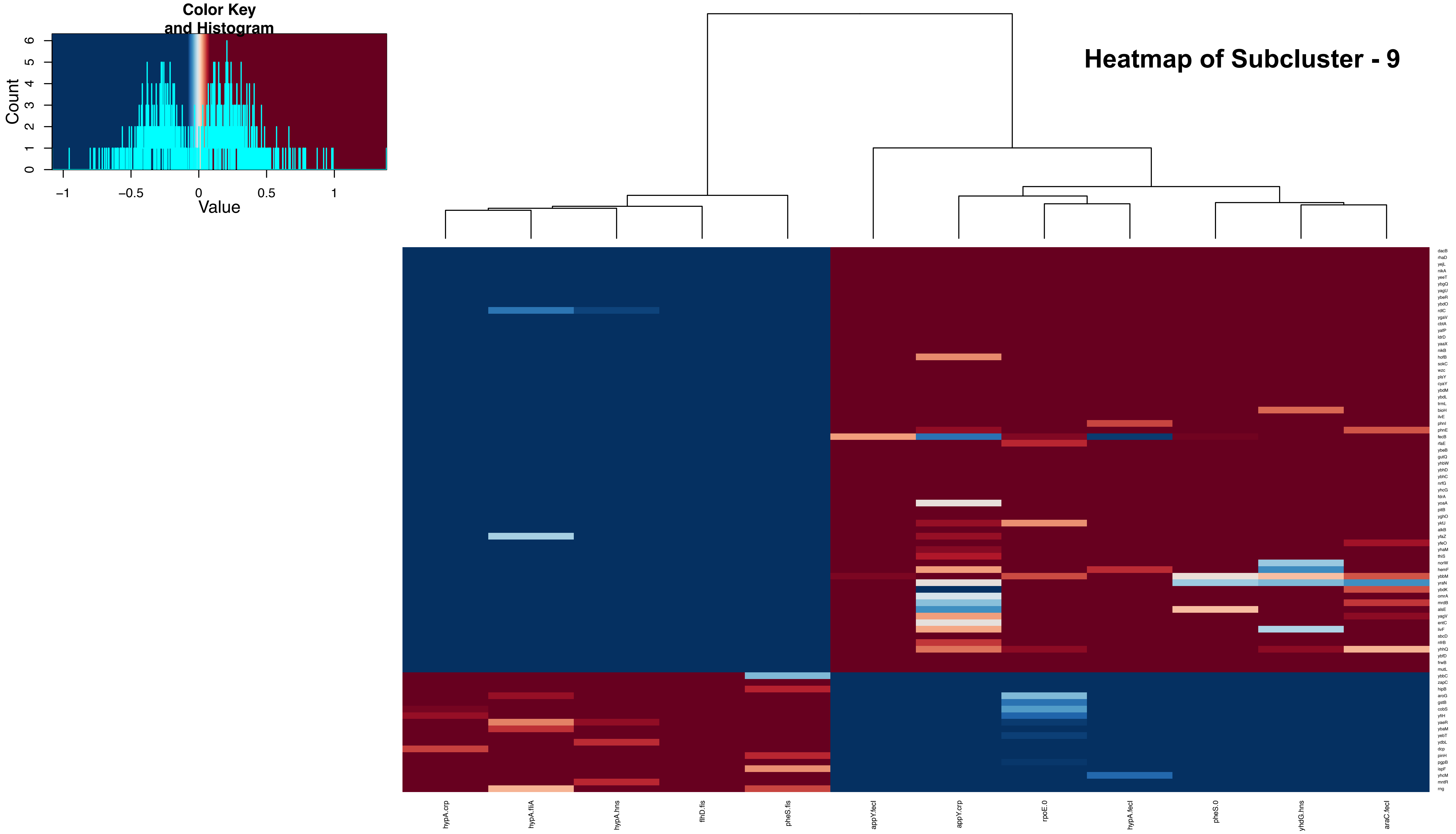
Color Key
and Histogram
Count
0
1
2
3
4
5
6
−1
−0.5
0
0.5
1
Value
Heatmap of Subcluster - 9
hypA.crp
hypA.fliA
hypA.hns
flhD.fis
pheS.fis
appY.fecI
appY.crp
rpoE.0
hypA.fecI
pheS.0
yhdG.hns
araC.fecI

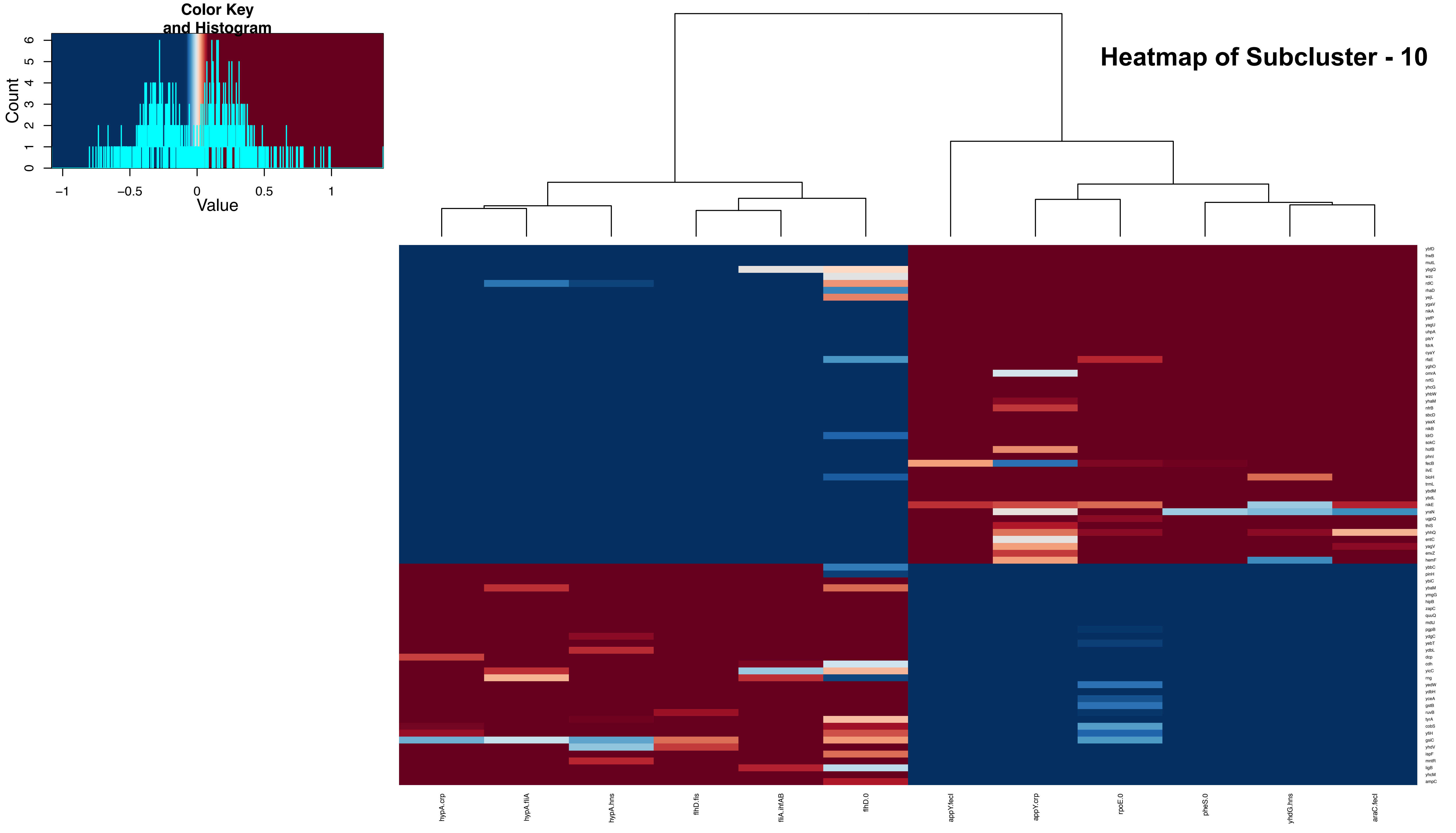

Heatmap of Subcluster - 10
Color Key
and Histogram
Count
0
1
2
3
4
5
6
Value
−1
−0.5
0
0.5
1
hypA.crp
hypA.fliA
hypA.hns
flhD.fis
fliA.ihfAB
flhD.0
appY.fecI
appY.crp
rpoE.0
pheS.0
yhdG.hns
araC.fecI
ybfD
frwB
mutL
ybgQ
wzc
rdlC
rhaD
yejL
ygaV
nikA
yafP
yagU
uhpA
plsY
fdrA
cyaY
rfaE
yghO
omrA
nrfG
yhcG
yhbW
yhaM
nfrB
sbcD
yaaX
nikB
ldrD
sokC
hofB
phnI
fecB
ilvE
bioH
trmL
ybdM
ybdL
nikE
yraN
ugpQ
thiS
yhhQ
entC
yagV
envZ
hemF
ybbC
pinH
ybiC
ybaM
ymgG
hipB
zapC
quuQ
mdtJ
pgpB
ydgC
yebT
ydbL
dcp
cdh
yicC
rng
yedW
ydbH
yceA
gstB
ruvB
tyrA
cobS
yfiH
gsiC
yhdV
ispF
mntR
ligB
yhcM
ampC

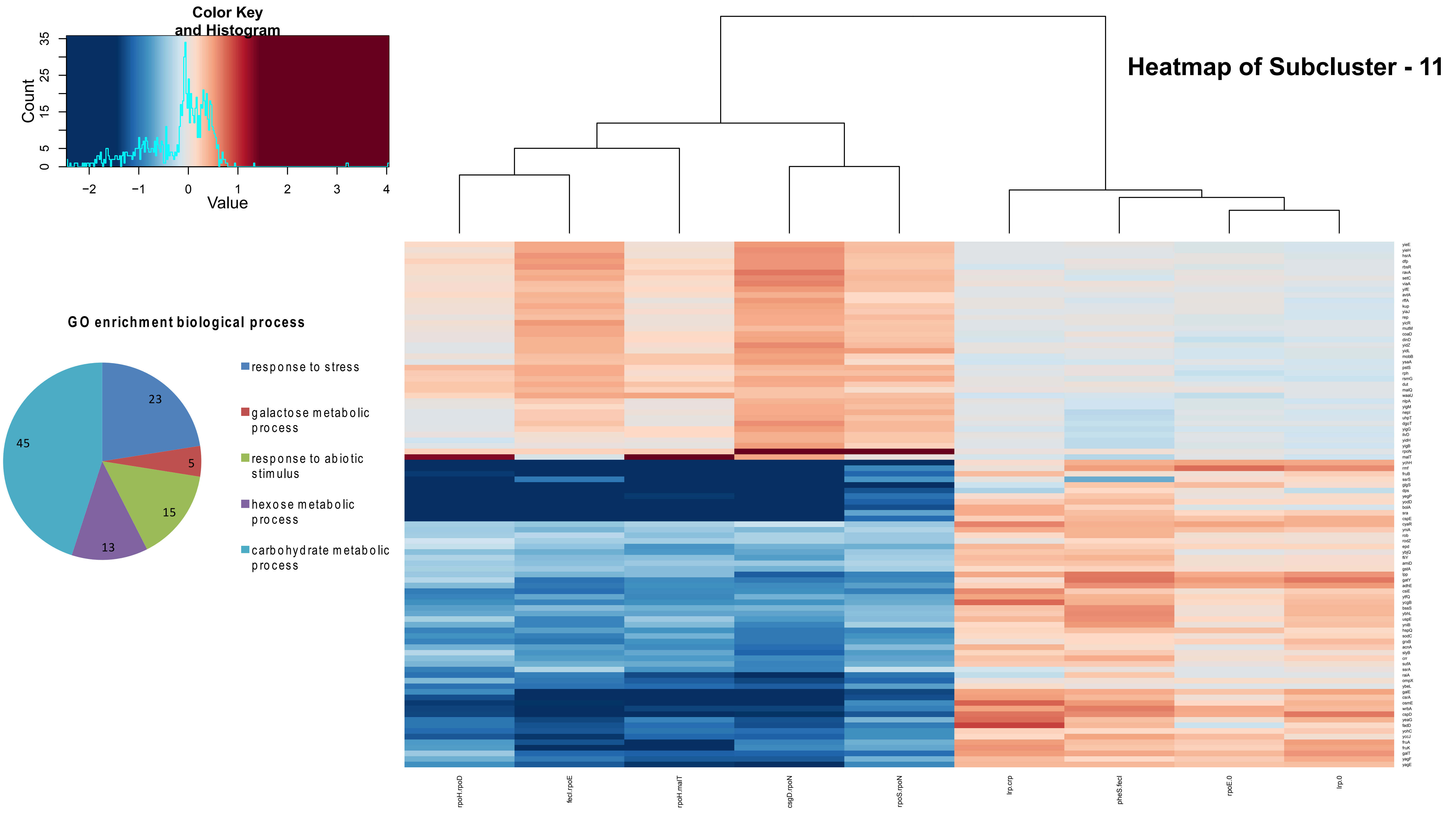

Color Key
and Histogram
Count
Value
−2
−1
0
1
2
3
4
0
5
15
25
35
Heatmap of Subcluster - 11
GO enrichment biological process
23
5
15
13
45
response to stress
galactose metabolic process
response to abiotic stimulus
hexose metabolic process
carbohydrate metabolic process
rpoH.rpoD
fecI.rpoE
rpoH.malT
csgD.rpoN
rpoS.rpoN
lrp.crp
pheS.fecI
rpoE.0
lrp.0

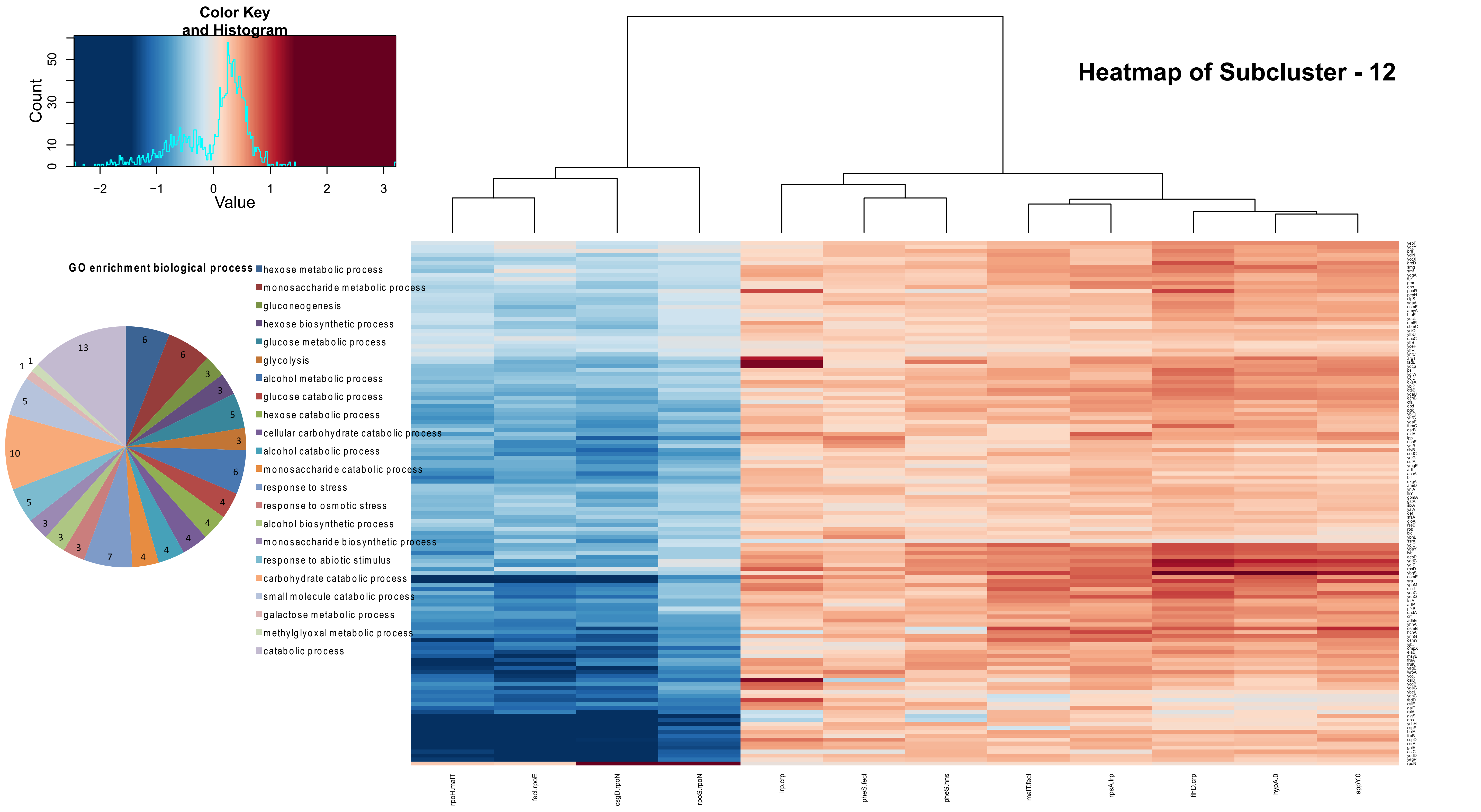

Color Key
and Histogram
Count
Value
Heatmap of Subcluster - 12
GO enrichment biological process
hexose metabolic process
monosaccharide metabolic process
gluconeogenesis
hexose biosynthetic process
glucose metabolic process
glycolysis
alcohol metabolic process
glucose catabolic process
hexose catabolic process
cellular carbohydrate catabolic process
alcohol catabolic process
monosaccharide catabolic process
response to stress
response to osmotic stress
alcohol biosynthetic process
monosaccharide biosynthetic process
response to abiotic stimulus
carbohydrate catabolic process
small molecule catabolic process
galactose metabolic process
methylglyoxal metabolic process
catabolic process
rpoH.malT
fecI.rpoE
csgD.rpoN
rpoS.rpoN
lrp.crp
pheS.fecI
pheS.hns
malT.fecI
rpsA.lrp
flhD.crp
hypA.0
appY.0

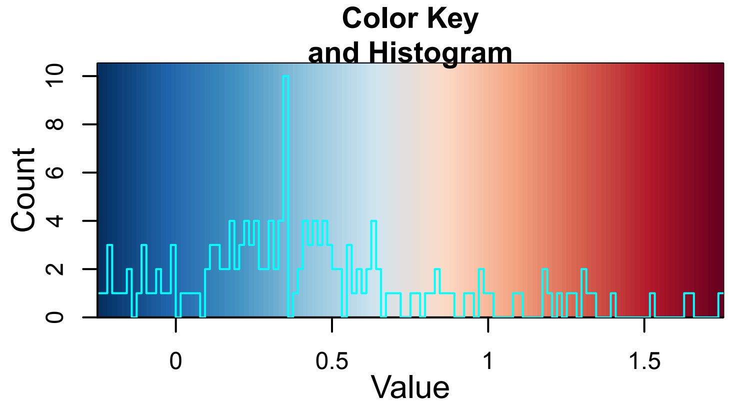

Heatmap of SubCluster − 13

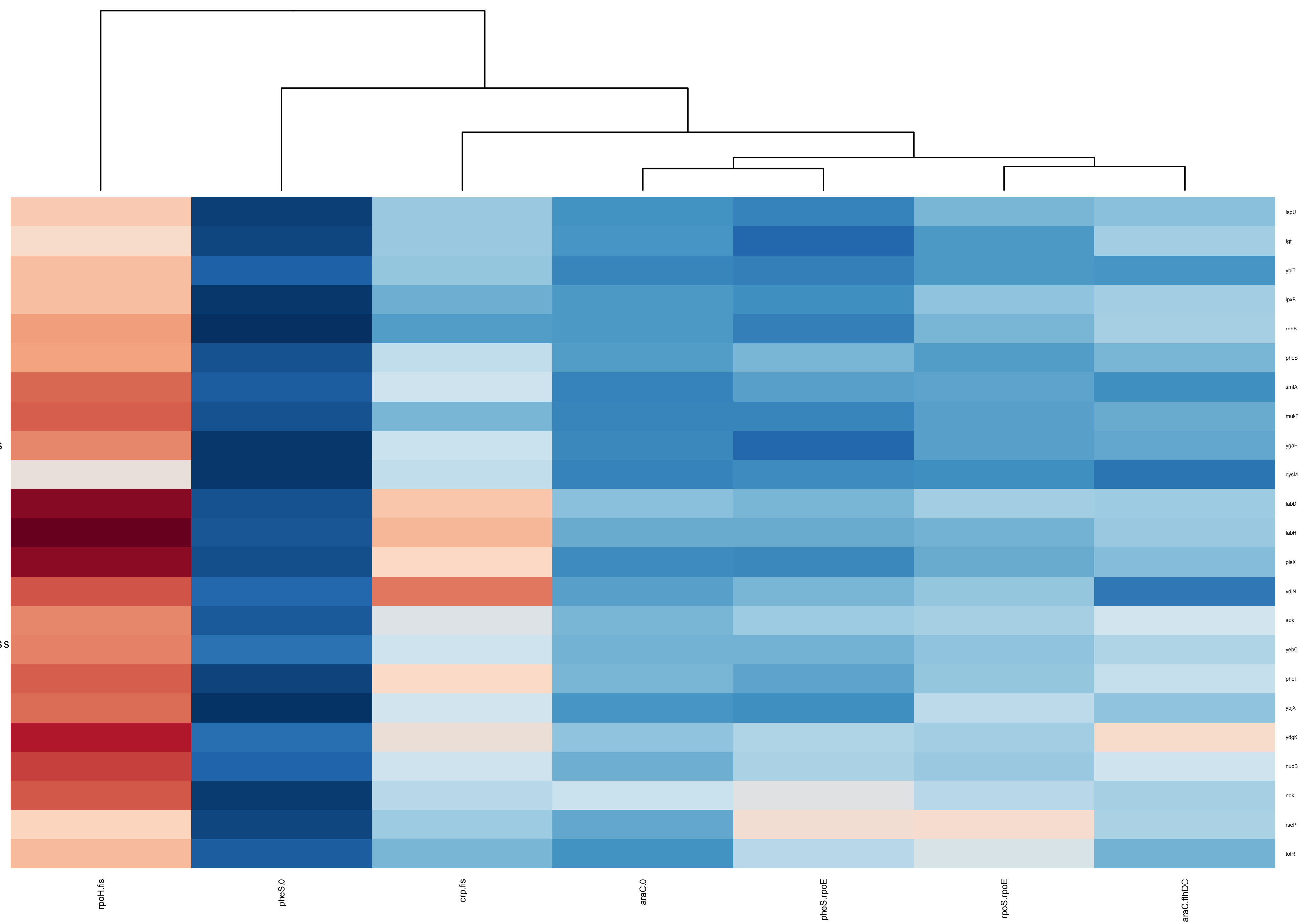

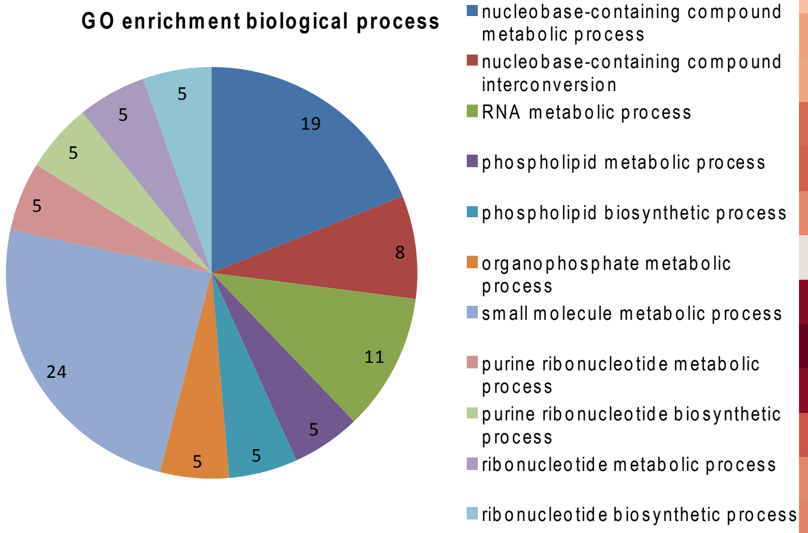

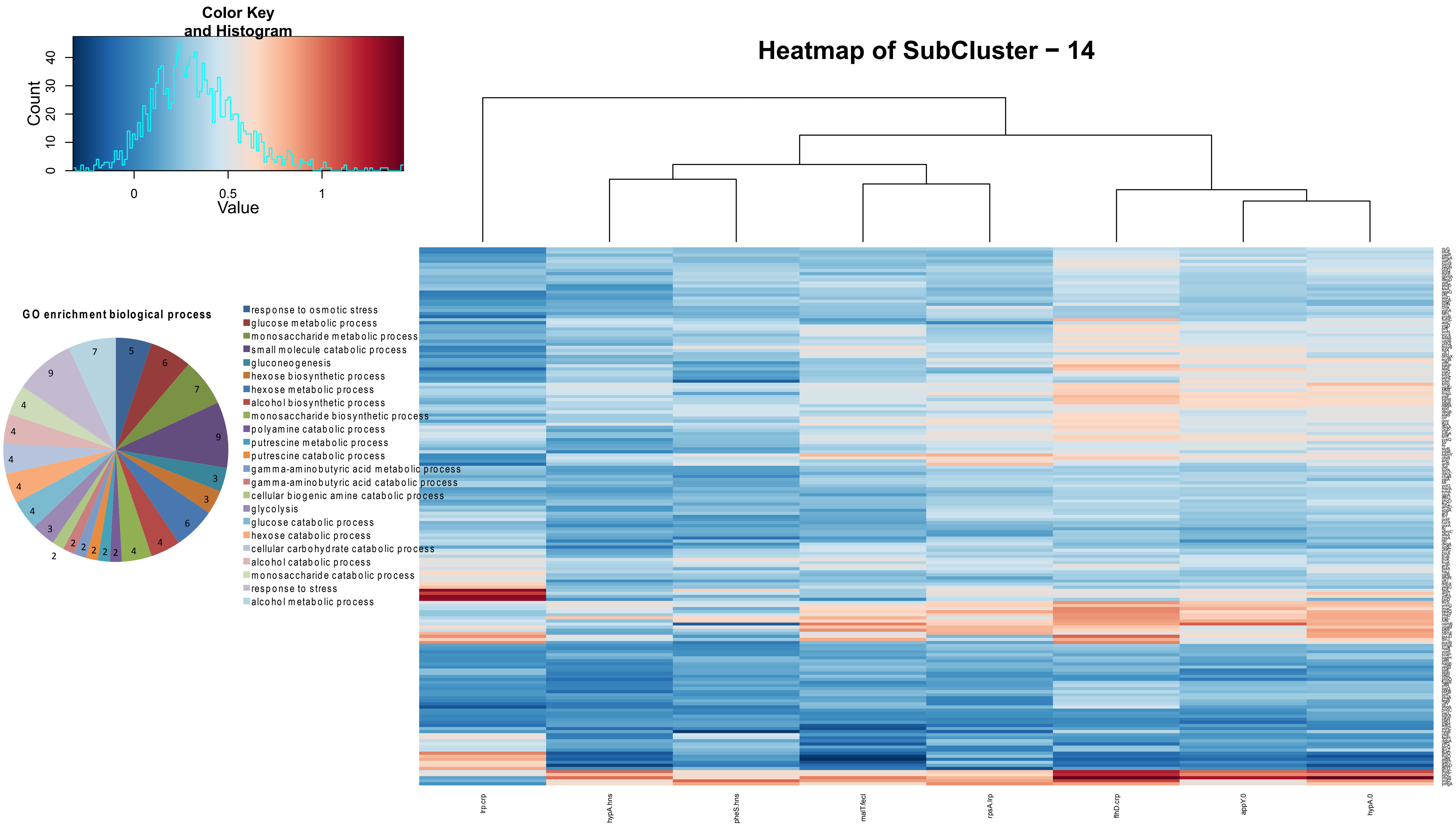
Color Key
and Histogram
Count
Value
0
0.5
1
0
10
20
30
40
Heatmap of SubCluster − 14
GO enrichment biological process
response to osmotic stress
glucose metabolic process
monosaccharide metabolic process
small molecule catabolic process
gluconeogenesis
hexose biosynthetic process
hexose metabolic process
alcohol biosynthetic process
monosaccharide biosynthetic process
polyamine catabolic process
putrescine metabolic process
putrescine catabolic process
gamma-aminobutyric acid metabolic process
gamma-aminobutyric acid catabolic process
cellular biogenic amine catabolic process
glycolysis
glucose catabolic process
hexose catabolic process
cellular carbohydrate catabolic process
alcohol catabolic process
monosaccharide catabolic process
response to stress
alcohol metabolic process
lrp.crp
hypA.hns
pheS.hns
malT.fecl
rpsA.lrp
flhD.crp
appY.0
hypA.0

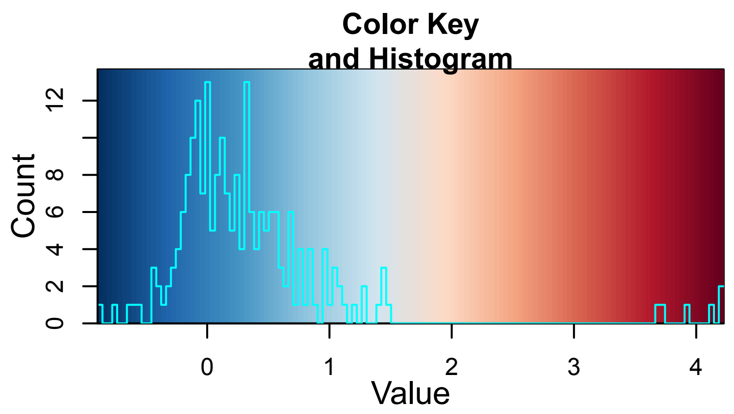
Color Key
and Histogram
Count
0
2
4
6
8
12
0
1
2
3
4
Value

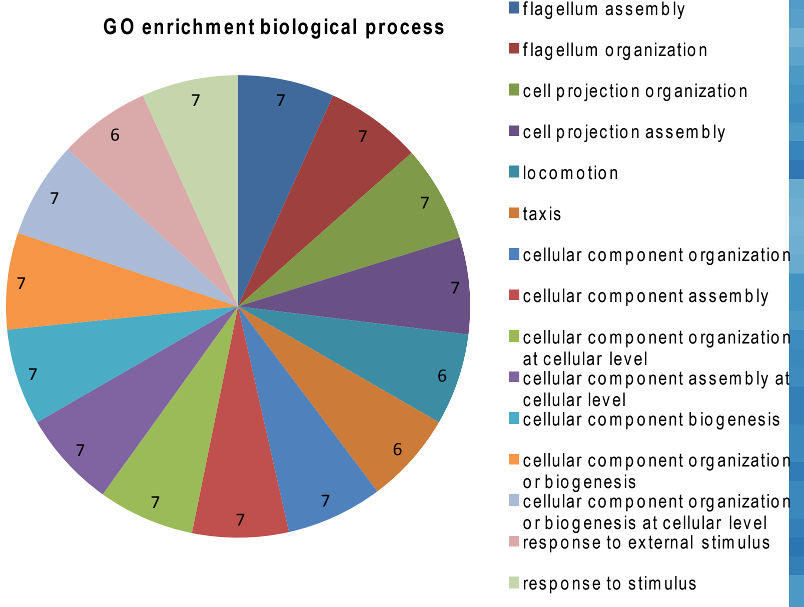
GO enrichment biological process
flagellum assembly
flagellum organization
cell projection organization
cell projection assembly
locomotion
taxis
cellular component organization
cellular component assembly
cellular component organization at cellular level
cellular component assembly at cellular level
cellular component biogenesis
cellular component organization or biogenesis
cellular component organization or biogenesis at cellular level
response to external stimulus
response to stimulus

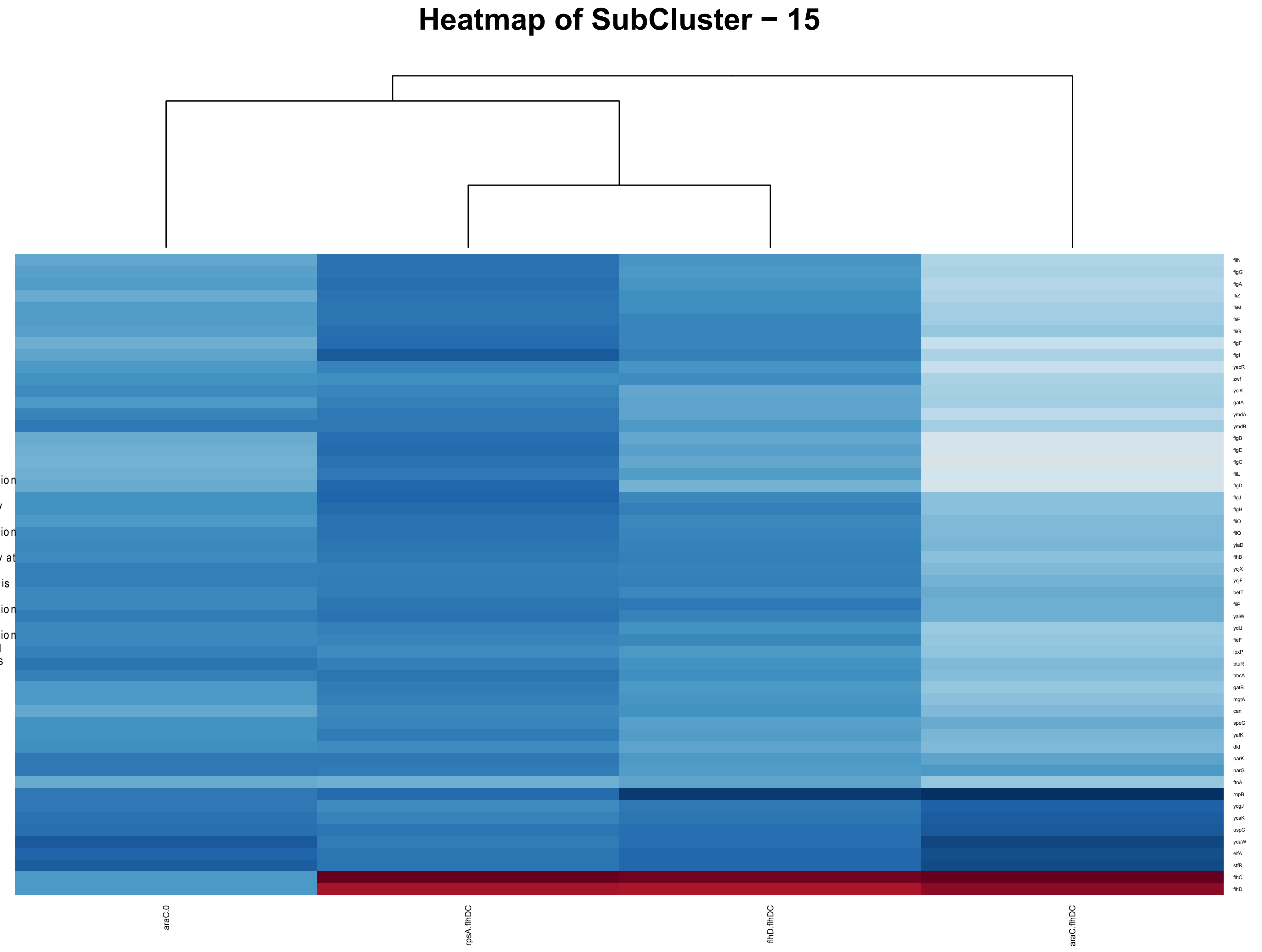
Heatmap of SubCluster − 15
fliN
flgG
flgA
fliZ
fliM
fliF
fliG
flgF
flgI
yecR
zwf
yciK
gatA
ymdA
ymdB
flgB
flgE
flgC
fliL
flgD
flgJ
flgH
fliO
fliQ
yiaD
flhB
ycjX
ycjF
betT
fliP
yaiW
ydiJ
fieF
lpxP
btuR
tmcA
gatB
mgtA
can
speG
yafK
dld
narK
narG
ftnA
rnpB
ycgJ
ycaK
uspC
ydaW
elfA
stfR
flhC
flhD
araC.0
rpsA.flhDC
flhD.flhDC
araC.flhDC

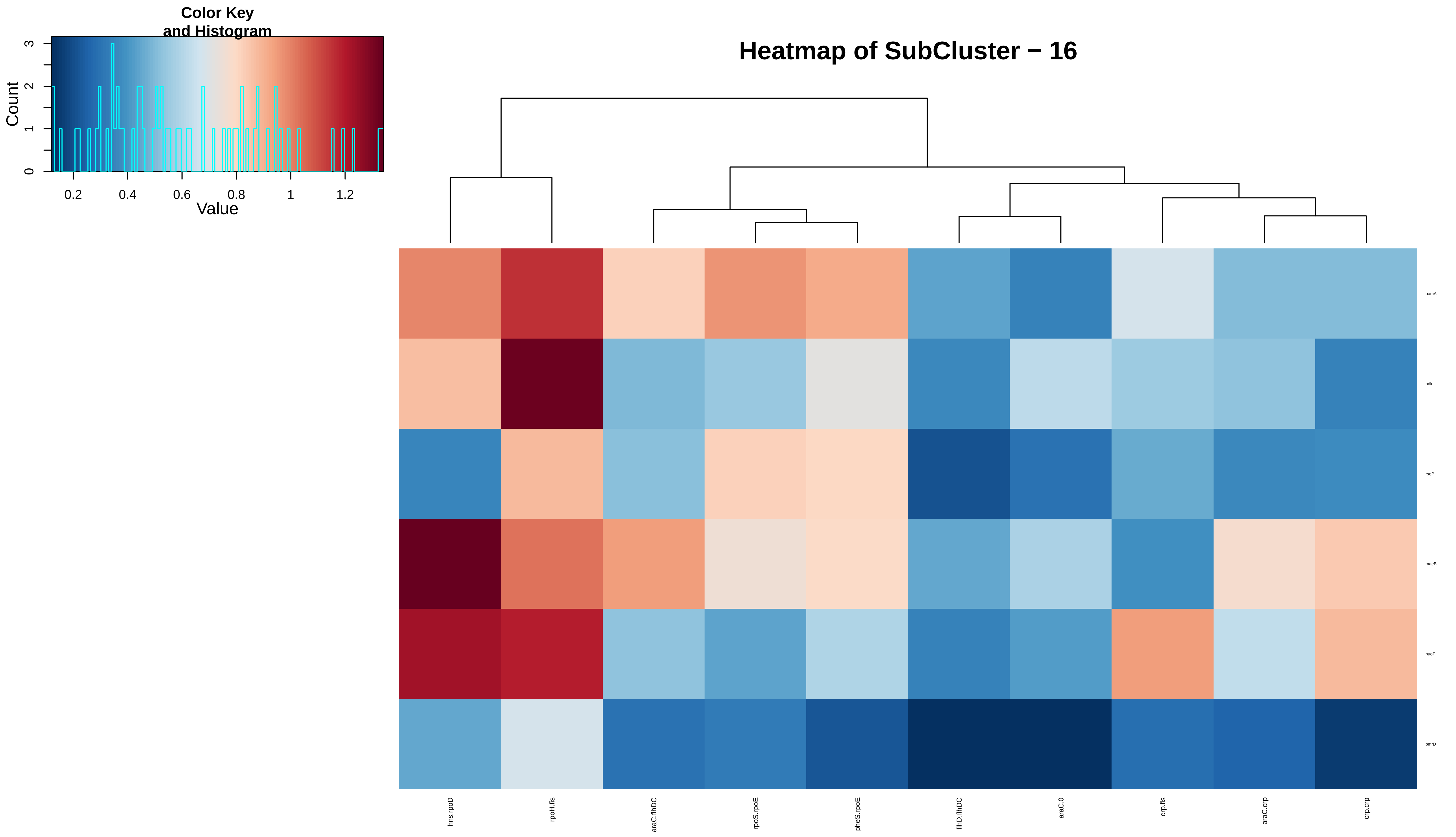
Color Key
and Histogram
Count
0
1
2
3
0.2
0.4
0.6
0.8
1
1.2
Value
Heatmap of SubCluster − 16
bamA
ndk
rseP
maeB
nuoF
pmrD
hns.rpoD
rpoH.fis
araC.flhDC
rpoS.rpoE
pheS.rpoE
flhD.flhDC
araC.0
crp.fis
araC.crp
crp.crp

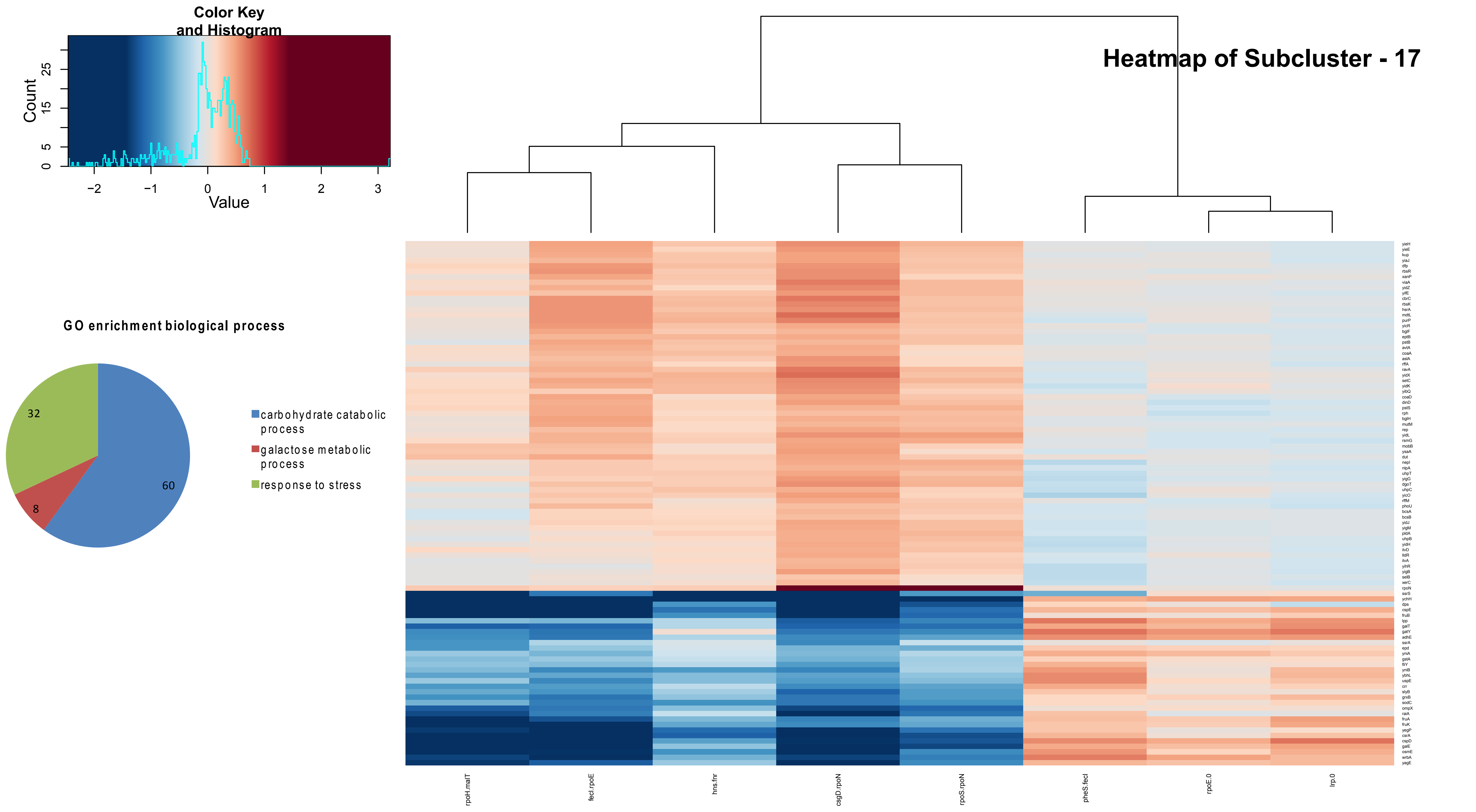
Color Key
and Histogram
Count
Value
0
5
15
25
−2
−1
0
1
2
3
Heatmap of Subcluster - 17
GO enrichment biological process
32
60
8
carbohydrate catabolic process
galactose metabolic process
response to stress
rpoH.malT
fecI.rpoE
hns.fnr
csgD.rpoN
rpoS.rpoN
pheS.fecI
rpoE.0
lrp.0

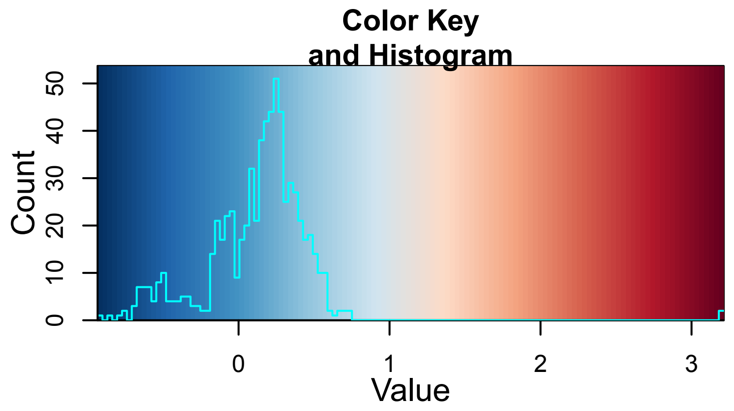
Color Key
and Histogram
Count
0
10
20
30
40
50
0
1
2
3
Value

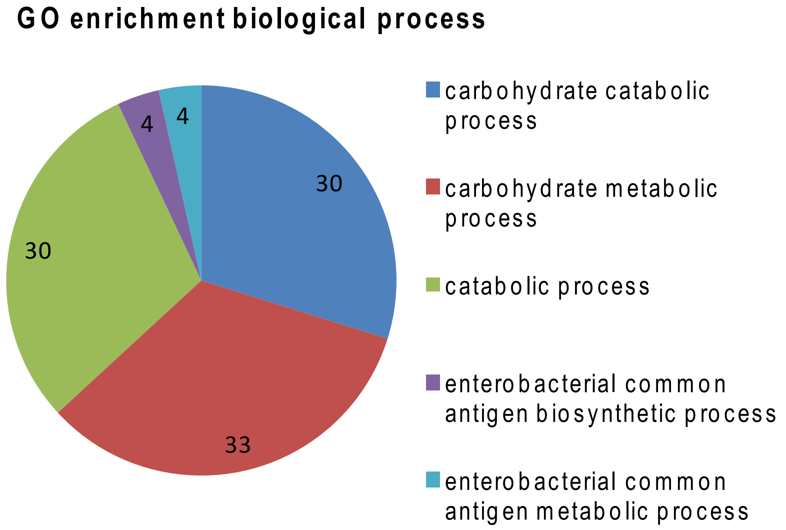
GO enrichment biological process
30
33
30
4
4
carbohydrate catabolic process
carbohydrate metabolic process
catabolic process
enterobacterial common antigen biosynthetic process
enterobacterial common antigen metabolic process

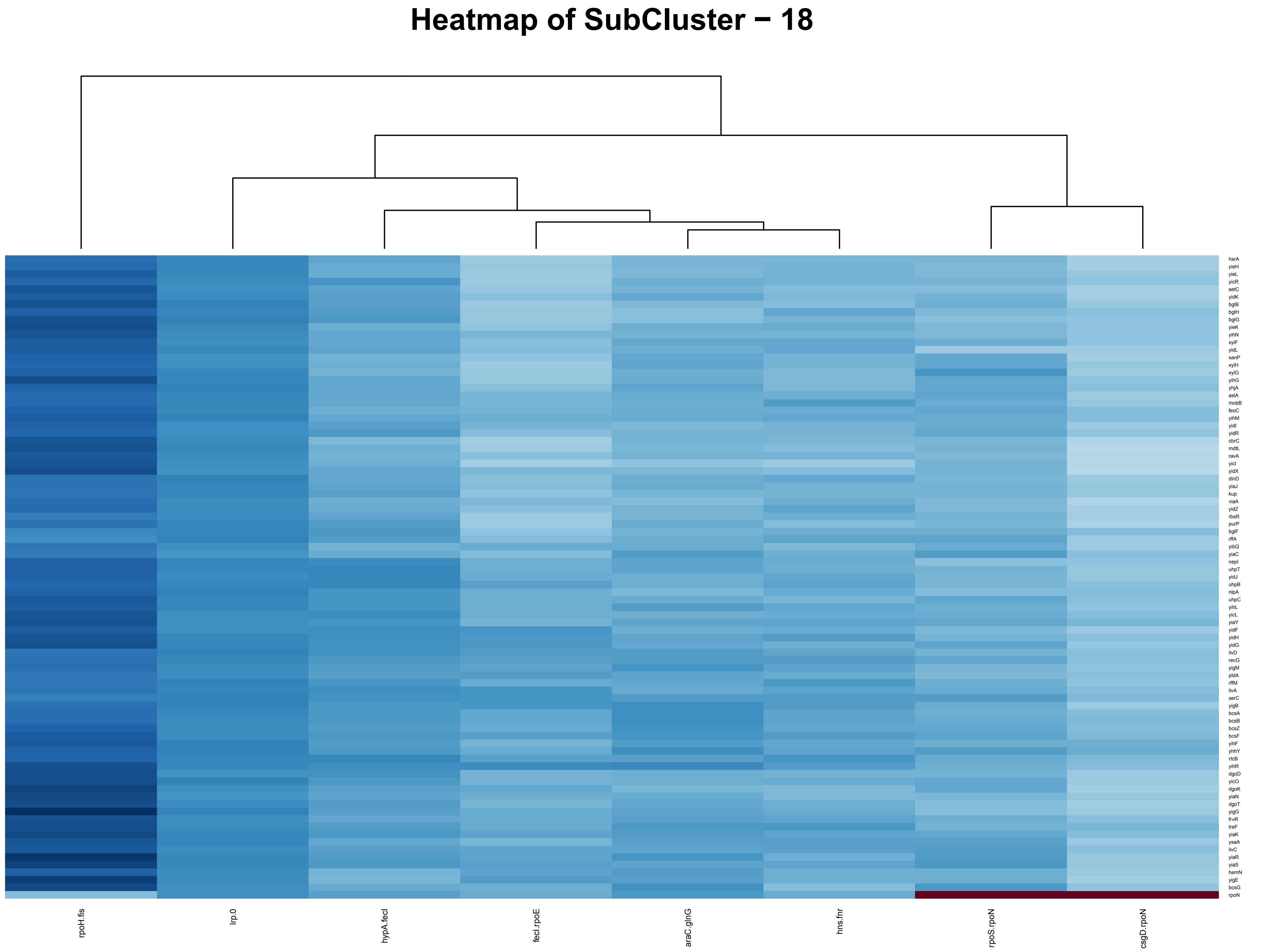
Heatmap of SubCluster − 18
hsrA
yieH
yieL
yicR
setC
yidK
bglB
bglH
bglG
yieK
yihN
xylF
yidL
xanP
xylH
xylG
yihG
yhjA
aslA
mobB
feoC
yihM
yidI
yidR
cbrC
mdtL
ravA
yicI
yidX
dinD
yiaJ
kup
viaA
yidZ
rbsR
purP
bglF
rffA
yibQ
yiaC
nepI
uhpT
yidJ
uhpB
nlpA
uhpC
yihL
yicL
yiaY
yidF
yidH
yidG
ilvD
recG
yigM
pldA
rffM
ilvA
xerC
yigB
bcsA
bcsB
bcsZ
bcsF
yihF
yhhY
rtcB
yihR
dgoD
yicO
dgoK
yiaN
dgoT
yigG
frvR
treF
yiaK
ysaA
ilvC
yiaR
yiaS
hemN
yigE
bcsG
rpoN
rpoH.fis
lrp.0
hypA.fecI
fecI.rpoE
araC.glnG
hns.fnr
rpoS.rpoN
csgD.rpoN

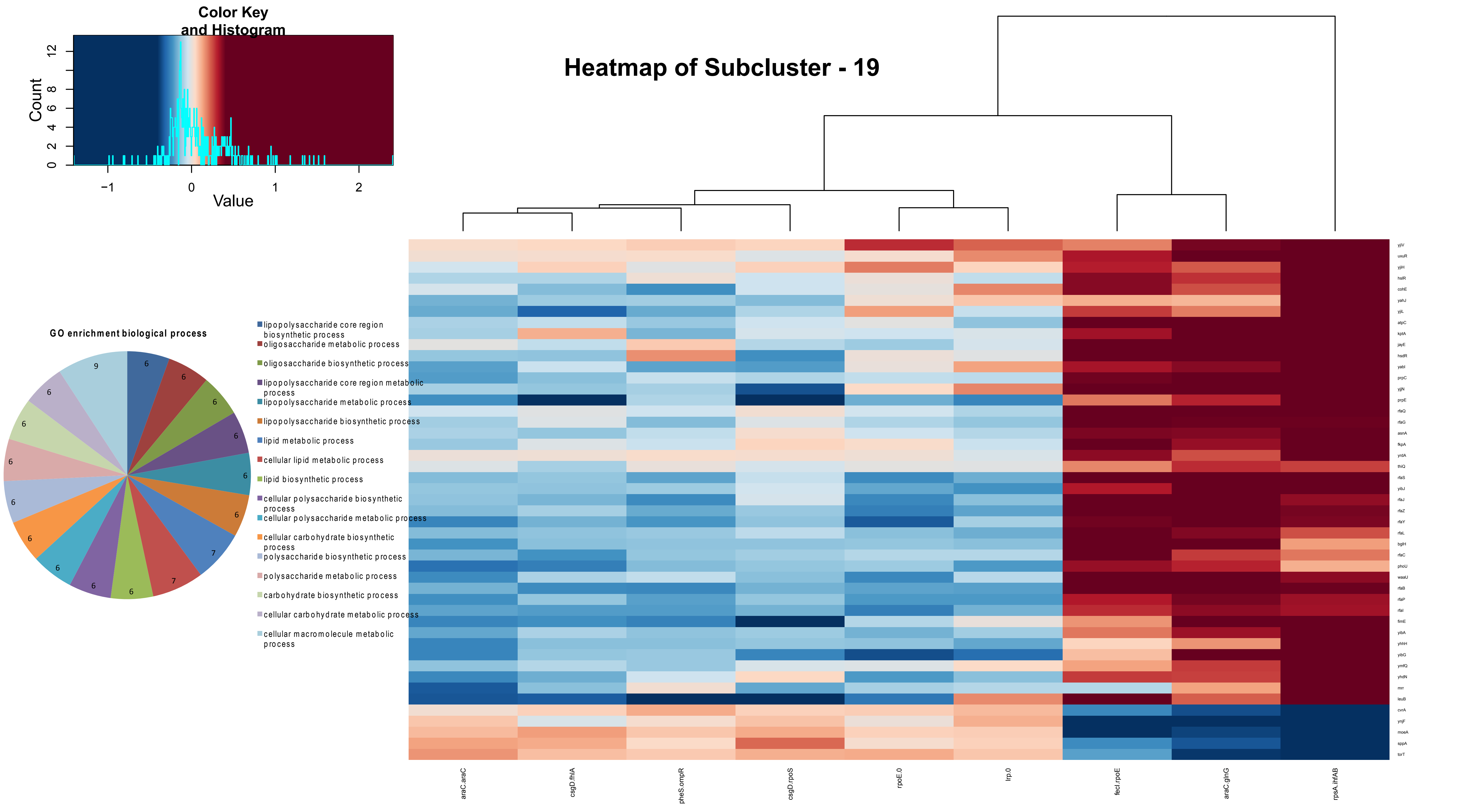

Color Key
and Histogram
Count
12
8
6
4
2
0
−1
0
1
2
Value
Heatmap of Subcluster - 19
GO enrichment biological process
9
6
6
6
6
6
6
7
7
6
6
6
6
6
6
6
lipopolysaccharide core region biosynthetic process
oligosaccharide metabolic process
oligosaccharide biosynthetic process
lipopolysaccharide core region metabolic process
lipopolysaccharide metabolic process
lipopolysaccharide biosynthetic process
lipid metabolic process
cellular lipid metabolic process
lipid biosynthetic process
cellular polysaccharide biosynthetic process
cellular polysaccharide metabolic process
cellular carbohydrate biosynthetic process
polysaccharide biosynthetic process
polysaccharide metabolic process
carbohydrate biosynthetic process
cellular carbohydrate metabolic process
cellular macromolecule metabolic process
yjiV
uxuR
yjiH
hslR
cohE
yahJ
yjiL
atpC
kptA
jayE
hsdR
yabI
prpC
yjjN
prpE
rfaQ
rfaG
asnA
fkpA
yrdA
thiQ
rfaS
yibJ
rfaJ
rfaZ
rfaY
rfaL
bglH
rfaC
phoU
waaU
rfaB
rfaP
rfaI
fimE
yibA
yhhH
yibG
ymfQ
yhdN
mrr
leuB
cvrA
ynjF
moeA
sppA
torT
araC.araC
csgD.fhlA
pheS.ompR
csgD.rpoS
rpoE.0
lrp.0
fecI.rpoE
araC.glnG
rpsA.infAB

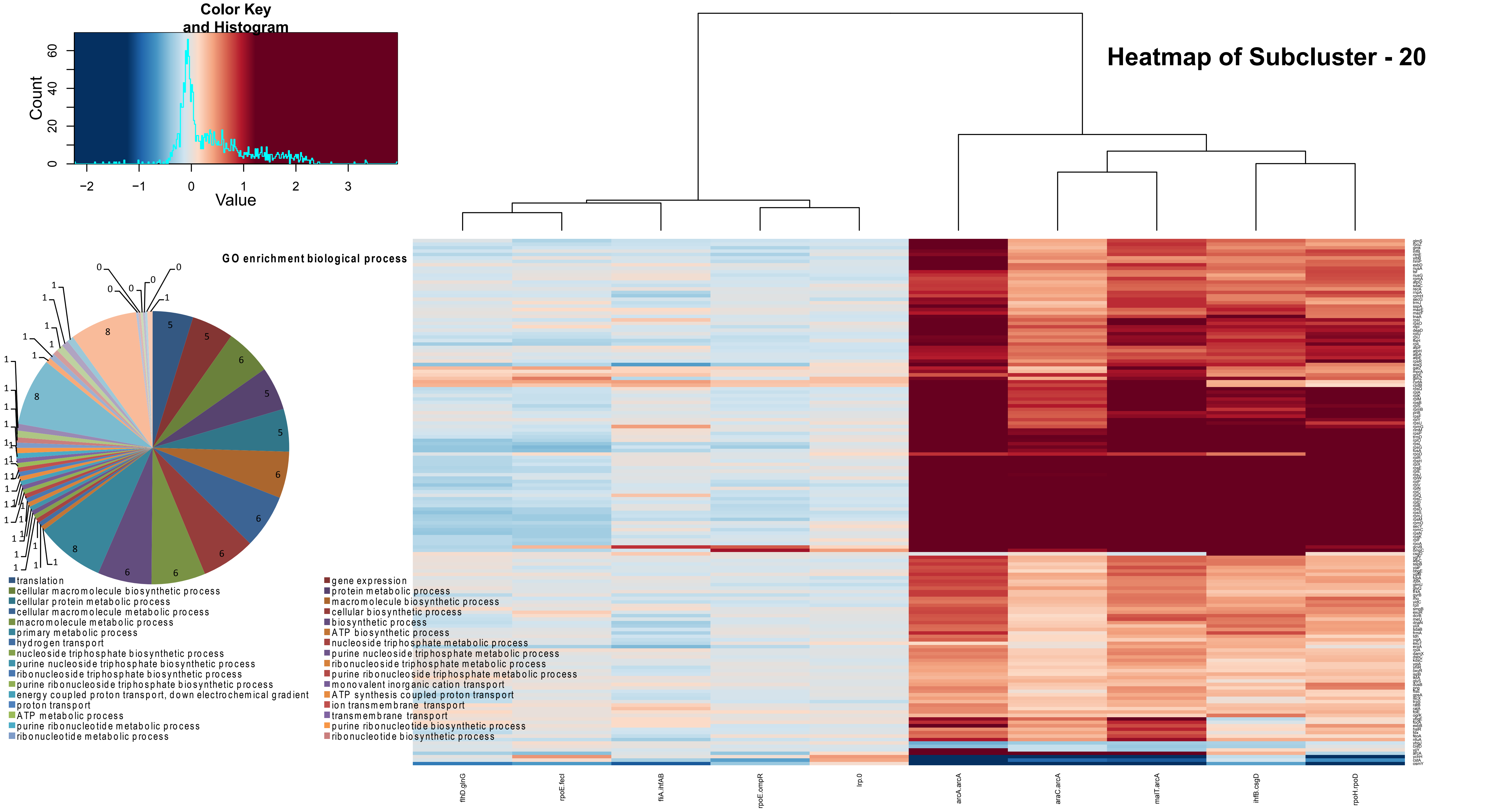
Heatmap of Subcluster - 20
Color Key
and Histogram
Count
Value
GO enrichment biological process
translation
cellular macromolecule biosynthetic process
cellular protein metabolic process
cellular macromolecule metabolic process
macromolecule metabolic process
primary metabolic process
hydrogen transport
nucleoside triphosphate biosynthetic process
purine nucleoside triphosphate biosynthetic process
ribonucleoside triphosphate biosynthetic process
purine ribonucleoside triphosphate biosynthetic process
energy coupled proton transport, down electrochemical gradient
proton transport
ATP metabolic process
purine ribonucleotide metabolic process
ribonucleotide metabolic process
gene expression
protein metabolic process
macromolecule biosynthetic process
cellular biosynthetic process
biosynthetic process
ATP biosynthetic process
nucleoside triphosphate metabolic process
purine nucleoside triphosphate metabolic process
ribonucleoside triphosphate metabolic process
purine ribonucleoside triphosphate metabolic process
monovalent inorganic cation transport
ATP synthesis coupled proton transport
ion transmembrane transport
transmembrane transport
purine ribonucleotide biosynthetic process
ribonucleotide biosynthetic process
flhD.glnG
rpoE.fecI
fliA.iraAB
rpoE.ompR
lrp.0
arcA.arcA
araC.arcA
malT.arcA
ihfB.csgD
rpoH.rpoD

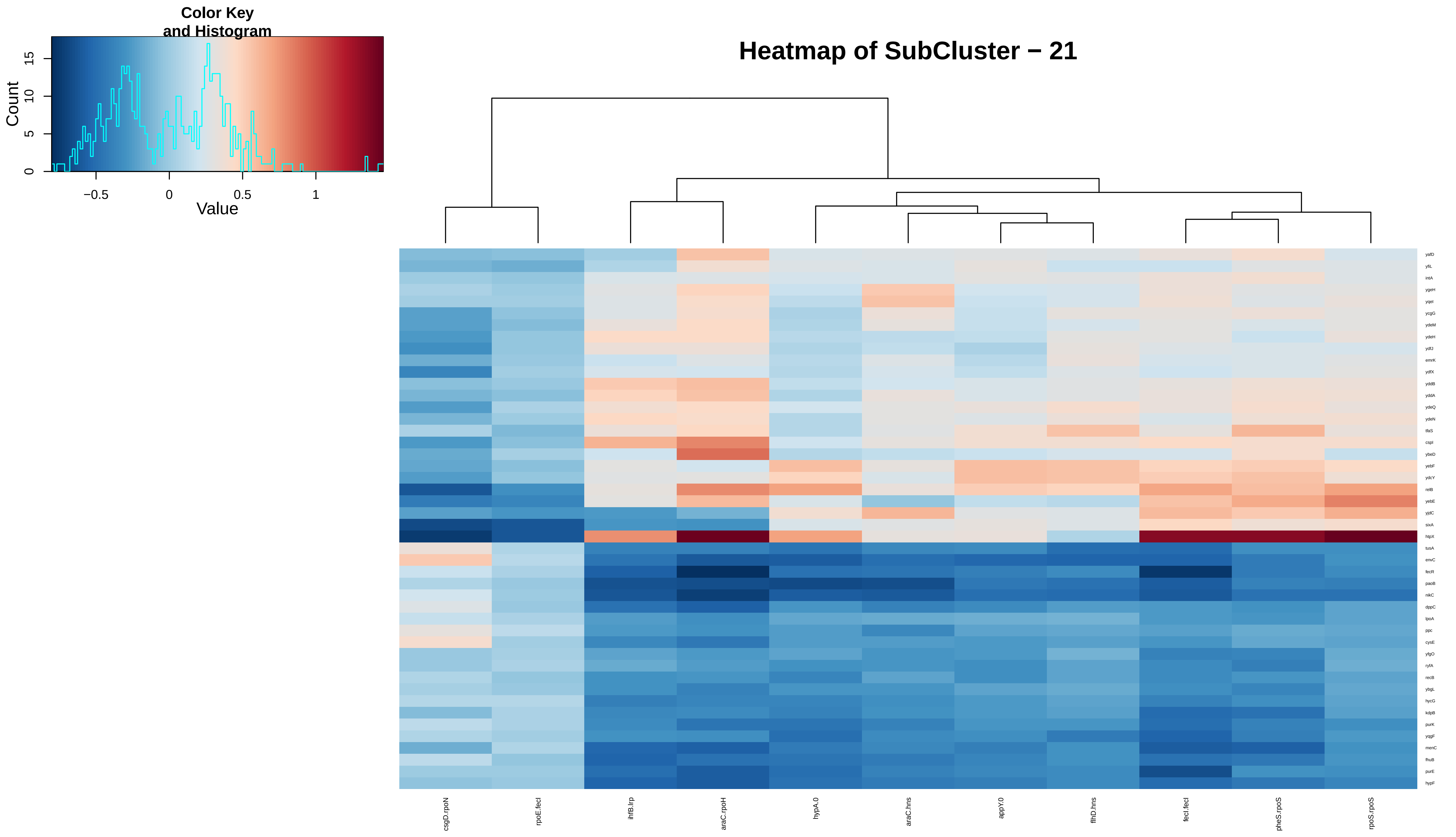

Color Key
and Histogram
Count
0
5
10
15
−0.5
0
0.5
1
Value
Heatmap of SubCluster − 21
yafD
yfiL
intA
ygeH
yqeI
ycgG
ydeM
ydeH
ydfJ
emrK
ydfX
yddB
yddA
ydeQ
ydeN
tfaS
cspI
ybeD
yebF
ydcY
relB
yebE
yjdC
sixA
htpX
tusA
envC
fecR
paoB
nikC
dppC
lpoA
ppc
cysE
yfgO
ryfA
recB
ybgL
hycG
kdpB
purK
yqgF
menC
fhuB
purE
hypF
csgD.rpoN
rpoE.fecI
ihfB.lrp
araC.rpoH
hypA.0
araC.hns
appY.0
flhD.hns
fecI.fecI
pheS.rpoS
rpoS.rpoS

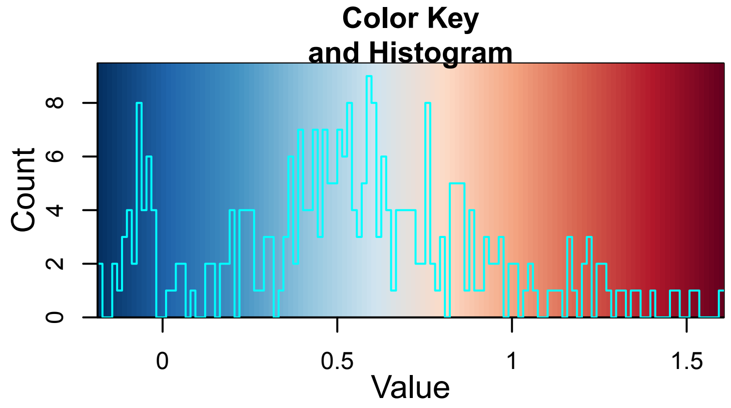

Heatmap of SubCluster − 22

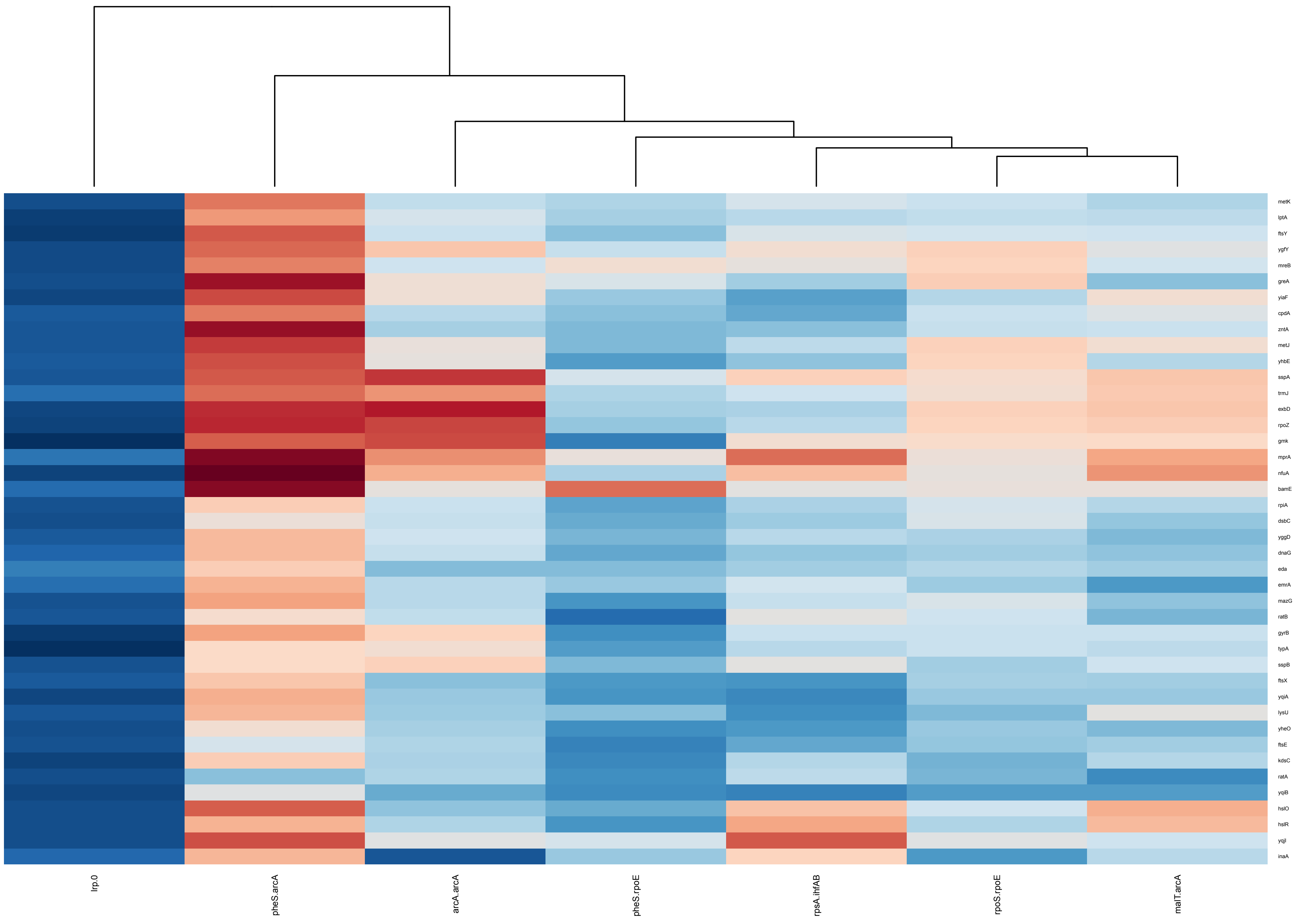

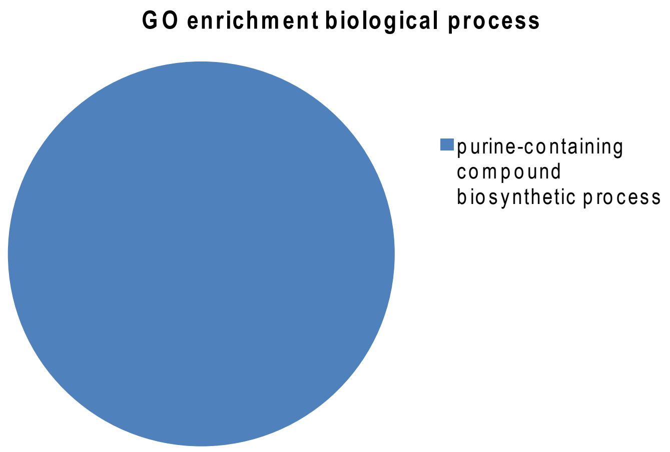

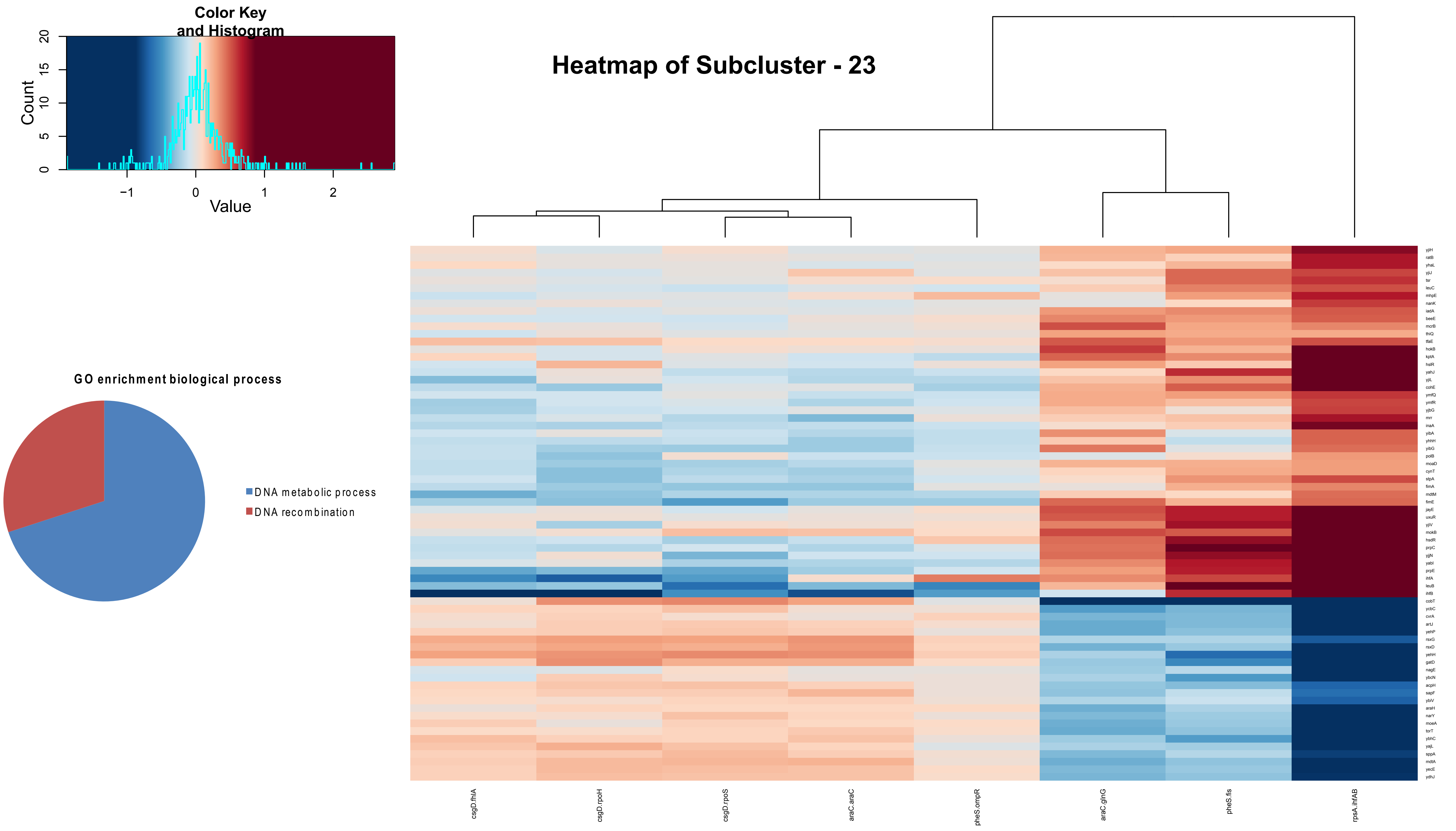

Color Key
and Histogram
Count
Value
Heatmap of Subcluster - 23
GO enrichment biological process
DNA metabolic process
DNA recombination
csgD.fhlA
csgD.rpoH
csgD.rpoS
araC.araC
pheS.ompR
araC.glnG
pheS.fis
rpsA.ihfAB
